# A Modified Oxazolone Dye Dedicated to Spectroscopy and Optoelectronics

**DOI:** 10.1021/acs.joc.2c00500

**Published:** 2022-05-19

**Authors:** Adam Szukalski, Przemysław Krawczyk, Bouchta Sahraoui, Faustyna Rosińska, Beata Jędrzejewska

**Affiliations:** † Faculty of Chemistry, Wroclaw University of Science and Technology, Wyb. Wyspiańskiego 27, Wrocław 50-370, Poland; ‡ Faculty of Pharmacy, Nicolaus Copernicus University, Collegium Medicum, Kurpińskiego 5, Bydgoszcz 85-950, Poland; § Laboratoire MOLTECH-Anjou, Université d’Angers, UFR Sciences, UMR 6200, CNRS, 2 Bd. Lavoisier, Angers Cedex 49045, France; ∥ Faculty of Chemical Technology and Engineering, Bydgoszcz University of Science and Technology, Seminaryjna 3, Bydgoszcz 85-326, Poland

## Abstract

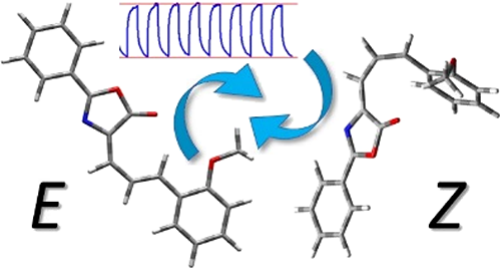

Here we present a newly synthesized bifunctional organic chromophore with appealing spectroscopic and nonlinear optical features. The positions of absorption and emission maxima of the dye vary with increasing solvent polarity and exhibit positive solvatochromism. The determined change in the dipole moment upon excitation based on the Bilot and Kawski theory is 5.94 D, which corresponds to the intermolecular displacement of a charge equal to 1.24 Å. An investigated organic-based system represents a significant, repeatable, and stable over time optical signal modulation in the manner of the refractive index value. Its magnitude is varied both by optical pumping intensity as well as by external frequency modulation, which indicates that such system is an alluring and alternative core unit for optoelectronic devices and complex networks. Then, the same active system, due to the nonresonant mechanism of higher harmonics of light inducement, can provide second and third harmonic signals. According to the introduced laser line spatial modifications (parallel or perpendicular polarization directions), it is resulted in output SHG signal with magnitude varied about 100%. Its magnitude is noticeably small; however, to construct sensitive optical sensors or infrared indicators, such feature may guarantee satisfying circumstances.

## Introduction

In the last few decades, there has been a particular interest in organic, multifunctional materials.^1−4^ Firstly and importantly, an organic matter is biofriendly, easy processable, and cheap. On the other hand, multifunctionality and versatility are the most desired molecular properties. Indeed, there are research fields, like photonics,^5,6^ optoelectronics,^7^ spectroscopy,^8,9^ medicine,^10^ and industrial areas, such as complex networks,^11^ optical databases,^12,13^ and logic and/or optical gates,^14^ where remote-controlled materials, especially those driven by light, are the most appealing ones. According to the reasons listed above, there are already few different approaches for designing such working organic systems, starting from organic low-molecular units embedded into branched and complex matrices^11,15^ and passing by nano-object utilization in both organic/inorganic systems^16^ up to the macromolecular, self-organizing, and functional systems.^17^ Understandably, each of them has advantages but also has drawbacks.

When considering the first mentioned low-molecular organic materials embedded in more sophisticated matrices, we need to discuss typical guest–host systems.^18−20^ An active part is typically composed of a small chromophore, which is responsible for matter vs light interaction, provides luminescence and light amplification phenomena,^18^ or modulates the output signal according to the particular mechanisms, i.e., by multiple photoinduced isomerizations.^21^ Sometimes, it is even possible to observe nonresonant interactions with strong coherent light generating in this way higher harmonics of light, such as second or third harmonic (SHG and THG, respectively).^22,23^ When focusing on the macromolecular architecture, usually it plays the role of stabilizing the whole system.^21^ In particular, the polymeric matrix is optically passive and transparent, which enables photons to penetrate its volume easily and reach active molecules. Furthermore, frequently, thanks to the particular branched structure, the polymer provides molecular hooking or hindrance for low-molecular active compounds (which depends on the available amount of free volume).^21^ Semi-intercalated and photoresponsive materials can follow various processes responsible for the aforementioned physical phenomena (light amplification, photoinduced conformational changes, etc.).

Here, we present an oxazolone derivative, abbreviated as Ox-π,π-Ph(OMe), which represents a delightful example of a multifunctional photoresponsive material. We have experimentally proved and theoretically supported the ability to generate two stable and photoinduced conformers responsible for effective refractive index changes. Such modification results in two optical features: its isotropic (initially) and anisotropic (light controlled) states. Such signal’s optical modulation realized in a range of seconds (static) and microseconds (dynamic mode) allows for easy and effective optical data manipulation. Moreover, by implementing a high-power picosecond laser system, it was able to generate significant SHG and THG signals, whose magnitude was also tunable according to the implemented various rotation angles. Thus, we believe that such material’s development can pave the way to construct optoelectronic or spectroscopic highly responsive and effective organic-based devices.

## Results and Discussion

### Synthetic Procedure and Molecular Design

During our ongoing work toward the compounds for optoelectronics, we have demonstrated that the 4-(3′-phenyl-2′-propenylidene)-phenylooxazol-5(4*H*)-one reveals the desired nonlinear optical properties to serve as a productive light amplifier, generator of higher harmonics of light, and optical modulator in the sub-microsecond time scale.^22,24^ Therefore, we decided to modify its structure to obtain an oxazolone dye with desired properties for various applications in spectroscopy, photonics, and optoelectronics. Since one of the most widely applied methods for adjusting the photophysical properties of organic compounds is to play with the nature of the substituents,^25−27^ we have synthesized an oxazolone dye containing a methoxy group at the ortho position. We hope that the modification influences the electronic distribution within the molecule since the compound reveals the D-π-A with the 5-(4*H*)-oxazolone ring constituting an electron-withdrawing moiety and the phenyl ring and methoxy substituent being an electron-donating part. Additionally, such configuration of the molecule offers the possibility of isomerization or molecular rotation.

**Figure 1:**
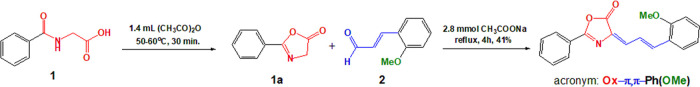
A route for the synthesis of 4-[3-(2-methoxyphenyl)prop-2-enylidene]-2-phenyl-1,3-oxazol-5-one (its acronym is Ox-π,π-Ph(OMe)) with marked significant regions: in red, oxazolone ring; in blue, two conjugated π-bonds creating stilbene groups; and in green, an electron donor group in the final product.

The 4-(3′-phenyl-2′-propenylidene)-phenylooxazol-5(4*H*)-one (Ox-π,π-Ph(OMe)) was synthesized in a moderate yield of 41% from hippuric acid and 2-methoxycinnamaldehyde as shown in Figure 1. The structure and purity of the dye were proved by NMR and IR analysis (see the Supporting Information).

**Figure 2:**
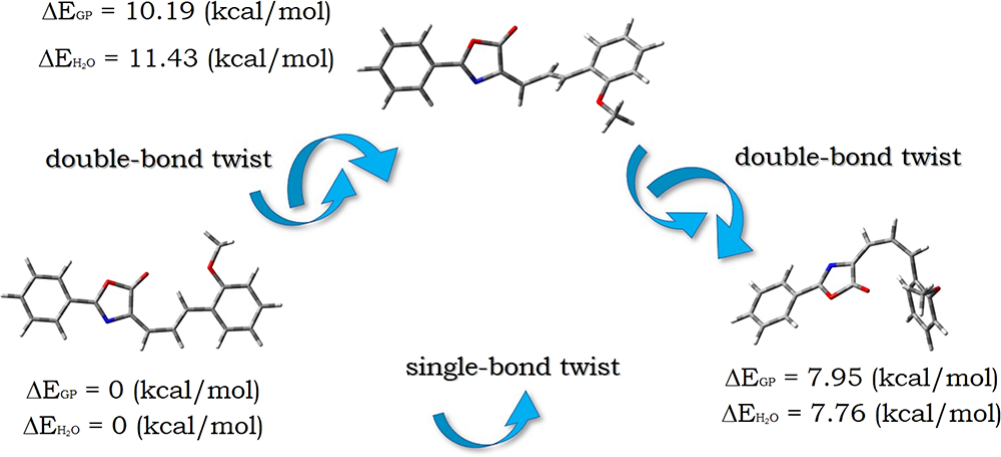
Optimized structures of the Ox-π,π-Ph(OMe) isomers.

To have an insight into the structure of the most stable *E* and *Z* isomers, the DFT equilibrium geometries of the compound were optimized in both the gas phase and water solution. Figure 2 illustrates the optimized structures of Ox-π,π-Ph(OMe), whereas all geometrical parameters are collected in Tables S2 and S3 in the Supporting Information.

Only the minima characterizing the lowest energy of *E* and *Z* isomers were considered in analyzing the linear and nonlinear optical properties of the dye. The double bond twist of the methine units leading to the *Z* conformer causes an increase in energy of 7.95 and 7.76 kcal/mol in the gas phase (GP) and water, respectively. The estimated *E* → *Z* isomerization barrier in GP is slightly more than 10 kcal/mol with a simultaneous increase to 11.43 kcal/mol in the water environment (Figure 2).

When analyzing the ground-state (GS) geometry of the dye in *E-Z* configuration, the distortion from planarity is clear. The dihedral angle between the ΘC6=C8–C9=C10 and ΘC8–C9=C10–C11 of the *E* conformer is 0.00165 and −179.99849°, respectively. During the forming of the TS structure, their values change to 91.54 and 179.98°, whereas the dihedral angles for the *Z* isomer are equal to 44.44 and 8.54°, respectively. This means that *E-Z* isomerization leads to the twisting of the molecule.

**Figure 3:**
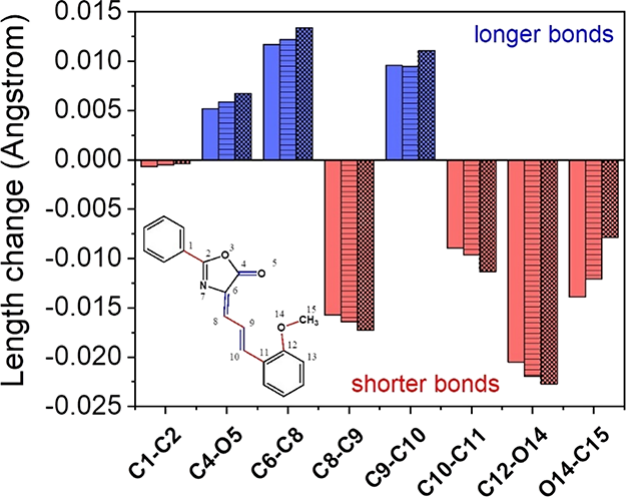
Topology of the studied structure and average changes in bond lengths during *E-Z* isomerization in GP (no pattern color), TMP (striped pattern color), and water (checkerboard pattern color). The red color illustrates bond shortening, whereas the blue one indicates bond elongation.

The analysis of structural parameters revealed a high sensitivity of bond lengths and dihedral angles of Ox-π,π-Ph(OMe) to the environmental changes, as well as to those occurring during photoexcitation to the first singlet excited state (*S*_CT_). The most important selected geometry parameters for these conformers (Figure S1) are listed in Tables S2 and S3 in the Supporting Information. Figure 3 illustrates the changes in bond lengths of *E* and *Z* conformers in GP, TMP, and water as indicators of the geometrical perturbations. In the other solvents tested, the same bonds were shorter or longer regardless of *E-Z* isomerization.

The greatest change in geometry takes place within the conjugated methine bonds connecting the phenyl ring with the oxazolone core, i.e., in the central part of the dye. The π-electron bridge is also affected by the environment polarity. A more polar environment favors the elongation of the C6=C8 and C9=C10 double bonds with simultaneous reduction of C8–C9 and C10–C11 single bonds. The length of the sternum also changes. The change in the length of these bonds is accompanied by an increase in the angles ΘC6=C8–C9 and C9=C10–C11, while the C8–C9=C10 angle is slightly shortened. Moreover, significant changes in the methoxy substituent are also found during *E-Z* isomerization. The polar solvent promotes the shortening of the C12–O14 bond, whereas at the same time it reduces the O14–C15 bond length differences between the *E* and *Z* isomers. Additionally, small changes in the C1–C2 bond length are also found for both isomers. Shortening this bond brings the phenyl ring closer to the oxazolone. However, this effect diminishes as the polarity of the solvent increases. At the same time, the C4=O5 double bond is elongated and the O5 oxygen atom approaches the O3 atom. This is the result of the decrease in the ΘO3–C4–O5 angle with the simultaneous increase in the ΘO5–C4–C6 and ΘO5–C4–C6–C8 angles.

In line with the theoretical data, the experimental results showed that the dye is not stable in a dilute solution. After 1 week of storing the dye in ethanol at room temperature, the absorption and fluorescence spectra are blue shifted by approximately 77 and 83 nm, respectively (Figure S2 in the Supporting Information). Changes in their spectral position may indicate the conversion of the *E* isomer to the *Z* one or the nonspecific interaction with the solvent, i.e., the formation of hydrogen bonds between the solute and solvent molecules.^28^

### UV–Vis Absorption and Fluorescence Spectra in Solutions

**Figure 4:**
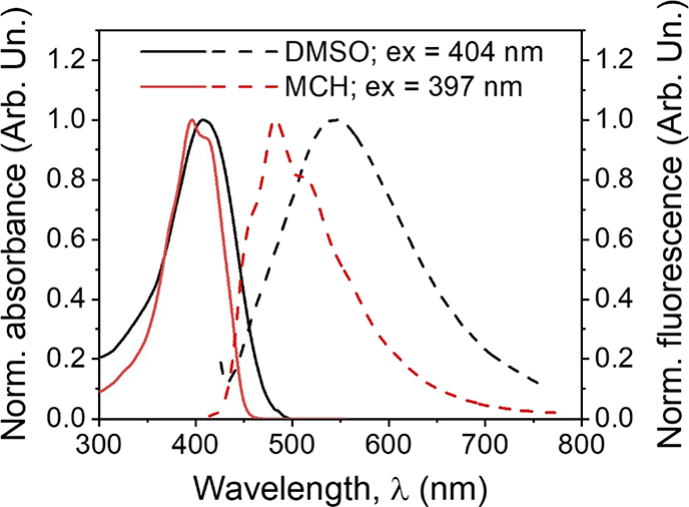
The normalized electronic absorption (solid) and fluorescence (dashed) spectra of the Ox-π,π-Ph(OMe) dye in methylcyclohexane (red) and dimethyl sulfoxide (black) organic solvents.

Figure 4 and Figure S3a in the Supporting Information illustrate the solvent effect on the shape and position of the electronic absorption and fluorescence spectra of the Ox-π,π-Ph(OMe) dye, whereas the basic photophysical data obtained for the tested dye in solvents of different polarity are collected in Table 1.

**Table 1:** The Main Photophysical parameters of the Ox-π,π-Ph(OMe) Dye, i.e., Absorption Maxima (λ_max_^Ab^, nm), Maximum Extinction Coefficient (ε, 10^4^ M^–1^ cm^–1^), Fluorescence Maxima (λ_max_^Fl^, nm), Full Width at Half-Maximum (FWHM; cm^–1^), Stokes Shift (Δν, cm^–1^), and Fluorescence Quantum Yield (ϕ_Fl_*,*%)

solvent	λ_max_^Ab^	ε	FWHM^Ab^	λ_max_^Fl^	FWHM^Fl^	Δν	ϕ_Fl_
Hex	393.0	6.25	3625 ± 28	478	4279 ± 27	4520	0.204
TMP	393.5	5.67	3678 ± 28	479	4444 ± 25	4540	0.223
MCH	396.5	5.29	3689 ± 28	481	4270 ± 27	4430	0.235
Bu_2_O	398.5	4.50	3863 ± 26	492	4682 ± 22	4770	0.218
Et_2_O	398.0	4.59	3831 ± 26	500	4281 ± 37	5130	0.053
EtOAc	400.0	3.82	3845 ± 25	515	4857 ± 30	5580	0.061
THF	404.5	4.57	3918 ± 25	524	4718 ± 29	5640	0.057
MeAc	401.0	4.50	4112 ± 23	534	5042 ± 17	6210	0.039
MeCN	399.0	4.26	4147 ± 23	537	5077 ± 25	6440	0.026
DMF	409.0	5.01	4162 ± 25	545	4975 ± 12	6100	0.038
DMSO	407.5	3.84	4361 ± 28	546	5032 ± 9	6230	0.046
1-BuOH	412.0	4.87	4028 ± 24	507	5499 ± 24	4550	0.060
2-PrOH	411.5	4.52	3942 ± 24	513	4595 ± 31	4810	0.060
1-PrOH	411.0	3.90	4066 ± 24	510	4845 ± 23	4720	0.053
EtOH	410.0	4.24	4104 ± 24	517	4802 ± 31	5050	0.035
MeOH	407.5	4.52	4065 ± 23	531	5132 ± 19	5710	0.021

The spectral behavior of the tested dye is similar to the compound described in our previous work.^24^ The main absorption band is located at ∼393–412 nm and, in nonpolar aprotic solvents, exhibits a distinct fine vibration structure, which becomes almost structureless in a polar environment. The molar extinction coefficient has a large value (ε ∼ 3.82 × 10^4^–6.25 × 10^4^ M^–1^ cm^–1^) and is lower in polar solvents, although there is no clear correlation between its values and the solvent polarity. The excitation wavelength has no effect on the dye fluorescence spectra (Figure S4b in the Supporting Information), which retain the vibronic structure in a nonpolar environment. However, as the solvent polarity increases, they become structureless and are red-shifted (Figure 4 and Figure S3b in the Supporting Information). The bathochromic shift of the fluorescence band is ca. 68 nm in aprotic solvents and only reaches 24 nm in alcohols, possibly due to the hydrogen bonding ability of the solvents, which stabilizes the electronic states of the dye in a different way. The introduction of an electron-donating substituent to the phenyl ring causes a bathochromic effect compared to the analogous unsubstituted compound, Ox-π,π-Ph. The OMe group induces shifts of the absorption and fluorescence maxima of +12–+26 and +24–+54 nm, respectively, depending on the type of solvent that has a significant influence on their position.^24^

To assign the bands observed in the experimental absorption spectra and characterize the nature of the electronic transitions, theoretical spectral properties were determined (Tables S4–S6). They are discussed in the Supporting Information.

The tested dye has a slightly lower value of the fluorescence quantum yield, ϕ_Fl_, than the unsubstituted oxazolone (Table 1). These values drop from 0.2 to 0.02% as the solvent polarity increases. The low fluorescence quantum yields can be partially attributed to the interaction of the photoinduced intramolecular charge transfer (ICT), which effectively contributes to fluorescence quenching.^29^ The CT excited state is a covalent (bonding) state with opposing charge and bond localization relative to the ground state (GS). In addition, the decrease in FQY is related to vibrations and rotations around the single and double bonds separating the oxazolone scaffold from the side phenyl rings, responsible for the effective deactivation of the excited state by nonradiative processes. Moreover, in the protic solvents according to the work of Yang et al.,^30^ the reduction in FQY is associated with a hydrogen-bonding-induced quenching of fluorescence.

Fluorescence decays of Ox-π,π-Ph(OMe) in all solvents are at least biexponential. The determined fluorescence lifetimes consist of a shorter major component of ca. 100–200 ps and a longer one on the nanosecond scale (Table S7 in the Supporting Information). The main lifetime values are slightly reduced in a more polar environment with a concomitant increase of the longer component so that the average lifetimes rise with increasing solvent polarity from 0.16 ns in *n*-hexane to 2.81 ns in DMSO (Table S7 in the Supporting Information). Hence, the rate constant of radiative *k*_r_ deactivation decreases from 12.69 × 10^6^ to 0.16 × 10^6^ s^–1^, respectively, which is caused by the more polar nature of the solvent (Table S8 in the Supporting Information). There is no clear correlation between the fluorescence lifetimes and the dielectric properties of the solvents. Nevertheless, there is a downward and upward trend when considering the picosecond and nanosecond components, respectively.

**Figure 5:**
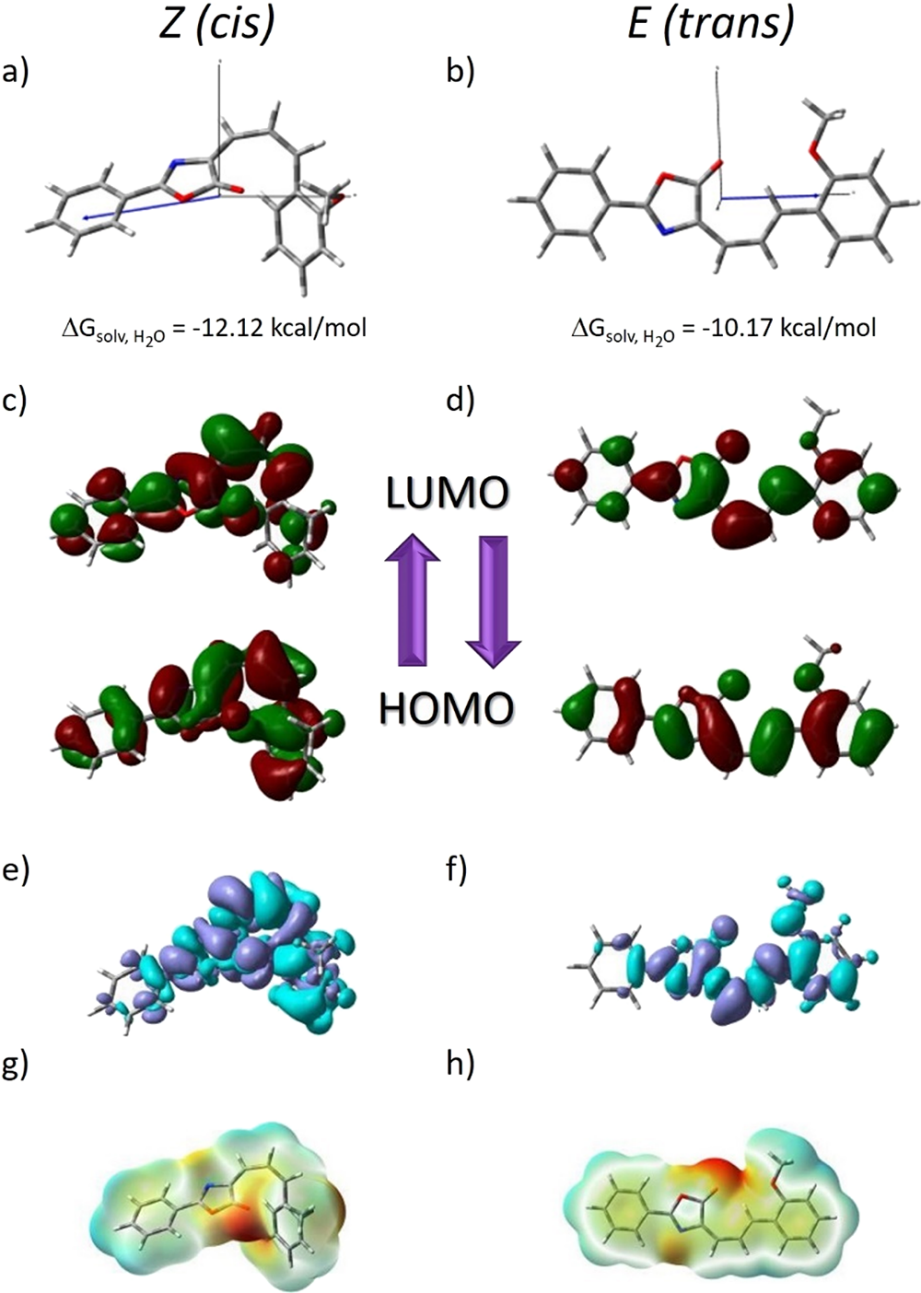
Electrochemical properties of the *Z* (left column) and *E* (right column) Ox-π,π-Ph(OMe) isomers. (a, b) Δ*G* energy in the water environment and orientation of the dipole moment vectors for the ground state; (c, d) HOMO–LUMO and their energy gap in the gas (3.59 and 3.15 eV) and water (3.59 and 3.10 eV) environment; (e, f) density difference for the gas (*D*_CT_ = 1.511 Å, *q*_CT_ = 0.510 e, and *D*_CT_ = 1.149 Å, *q*_CT_ = 0.372 e) and water (*D*_CT_ = 1.641 Å, *q*_CT_ = 0.501 e and *D*_CT_ = 1.656 Å, *q*_CT_ = 0.407 e) environment; and (g, h) MEP in the gas (±0.04324 and ±0.04888 a.u.) and water (±0.05108 and ±0.05952 a.u.) phase for *Z* and *E* isomers, respectively. In panels e and f, the blue (purple) zones indicate density decrease (increase) upon electronic transition.

For the tested dye, upon increasing the solvent polarity, a red-shifted emission can be found, with a significantly decreased quantum yield due to the vibrations and rotations around the single and double bonds and the ICT interaction. Additionally, the excited state is more polar than the ground state so that it is better stabilized by more polar solvents. Thus, the fast fluorescence decay mechanism may be attributed to relaxation from the locally excited state toward an intramolecular charge-transfer (ICT) state. The observed increase of the average fluorescence lifetimes in more polar environment may result from the more forbidden feature of the CT emission. In addition, as shown in Figure 5, during HOMO–LUMO transition, electrons delocalized on the entire surface of the dye in the ground state are transferred toward the oxazolone ring and π-bridge upon excitation, which will be favorable to inhibit the radiationless decay channel around the exocyclic double bond. The more electrons there are that transfer to the double bond bridge, the better is the *Z*/*E* photoisomerization inhibition, which can also influence the fluorescence lifetimes. However, the proper explanation of the time-resolved studies needs more research.^31−33^

The effect of the solvent properties on the absorption and fluorescence maximum shift, as well as the Stokes shift, was estimated based on the multiple regression analysis using Catalán’s solvent polarity parameters,^34^ i.e., polarizability (SP), dipolarity (SdP), acidity (SA), and basicity (SB).^34−36^ The best correlation results obtained by eq 1 are summarized in Table 2.
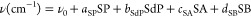
1

**Table 2:** Values Estimated Based on Eq 1 Coefficients (*y*_0_, *a*_SP_, *b*_SdP_, *c*_SA_, and *d*_SB_), Their Standard Errors and Correlation Coefficients (*R*^2^) According to the Multiple Linear Regression Analysis ν_ab,_ ν_fl_*i* Δν^SS^ for Ox-π,π-Ph(OMe) in 16 Different Solvents as a Function of Catalán’s Four-Parameter Scale of Solvents

*y*	ν_ab_	ν_fl_	Δν^SS^
*y*_0_	26,658 ± 452	22,002 ± 615	4657 ± 937
*a*_SP_	–(1901 ± 724) **50.4%**	–(1925 ± 986) **36.6%**	24 ± 1501 0.5%
*b*_SdP_	–(160 ± 128) 4.2%	–(2428 ± 174) **46.2%**	2268 ± 265 **46.3%**
*c*_SA_	–(1009 ± 233) 26.7%	533 ± 317 10.2%	–(1541 ± 483) 31.4%
*d*_SB_	–(704 ± 181) 18.7%	365 ± 246 7.0%	–(1069 ± 375) 21.8%
*R*^2^	0.927	0.970	0.895

To compare the data obtained based on multiparameter linear regression, the percentages of individual solvent polarity parameters were determined. According to the results, the Ox-π,π-Ph(OMe) dye behaves in terms of absorption, fluorescence, and Stokes shift in the solvents used similarly to the compound described previously.^24^ The solvent polarizability plays the most important role in the solvation of the solute with respect to absorption as indicated by the percentage of this polarity parameter. Since the absorption band position is mainly controlled by nonspecific interactions, the absorption arises from the polarized π–π* transition.^21^ At the same time, the negative sign indicates that the absorption band shifts toward longer wavelengths with increasing polarizability (SP), dipolarity (SdP), acidity (SA), and basicity (SB) of the environment. On the other hand, fluorescence is the most sensitive to changes in the polarizability (SP) and dipolarity (SdP) of the solvents. Its maximum is red-shifted in more polar solvents due to the high ratio between SP and SdP, although a hypsochromic shift is observed with respect to SA and SB, accordingly. However, the latter terms are negligible in fluorescence analysis since they are characterized by the lowest values and high standard errors. In addition, an excitation led to changes in SA and SB terms, which indicate the lower acidity and basicity of the excited state^37^ of the Ox-π,π-Ph(OMe) dye.

Following the experimental data, the Stokes shift changes by approximately 2010 and 1149 cm^–1^ with the solvent type, i.e., from 4431 cm^–1^ in MCH to 6441 cm^–1^ in MeCN and from 4548 cm^–1^ in 1-BuOH to 5707 cm^–1^ in MeOH, respectively. The increase in the Stokes shift in different environments is due to the increase in solvent dipolarity. The effect may relate to the separation of charges in the D-π-A dye molecule and creation of a dipolar structure differently oriented in space as observed in other compounds with a donor–acceptor substituent displaying a low or moderate effect.^38^

According to the computational calculation, the charge-transfer (CT) excitation for both conformers (*E* and *Z*) corresponds basically to the HOMO → LUMO transition (Figure 5). HOMO electrons are delocalized on the entire surface of the compound, while the LUMO is mostly on the π-electron bridge. The transfer of electrons from the benzene rings, as a donor group, toward the oxazolone and carbon bridge is observed. This indicates that the lowest-lying excited state can be assigned as a π–π* transition mixed with an intramolecular charge-transfer (ICT) process. Isomerization does not change the distribution of both frontier molecular orbitals.

There is also no significant change in the value of the energy separation between HOMO → LUMO orbitals (*E*_GAP_). The Δ*E*_GAP_^*E* – *Z*^ difference is 0.44 eV in the gas phase, and it is increased to 0.49 eV in the aqueous phase (Table S9 in the Supporting Information).

The density variation upon photoexcitation (Δρ(*r*)) calculated for the first electronic transition is graphically depicted in Figure 5 and Figure S5. The Δρ(*r*) plots show that the density depletion zones (blue) are mostly delocalized on the benzene rings. The regions of density increment (purple) are mostly localized on the carbon bridge and oxazolone ring. At the same time, the solvent polarity does not significantly change in the location of these regions. This is reflected in the amount of transferred charge (*q*_CT_) and charge-transfer distance (*D*_CT_). Both *q*_CT_ and *D*_CT_ values are large for the *Z* isomer. The Δρ_CT_^*Z* – *E*^ is 0.1 a.u. (Table S10 in the Supporting Information), while the Δ*D*_CT_^*Z* – *E*^ is 0.3 Å in less polar solvents and 0.05 Å in more polar solvents. Regardless of the isomer type, the *D*_CT_ indicates the CT character and confirms the contributions from HOMO → LUMO transition, although minor contributions from other orbitals should be expected (the frontier orbital energies in different solvents are described in Supporting Information, Table S9).

**Figure 6:**
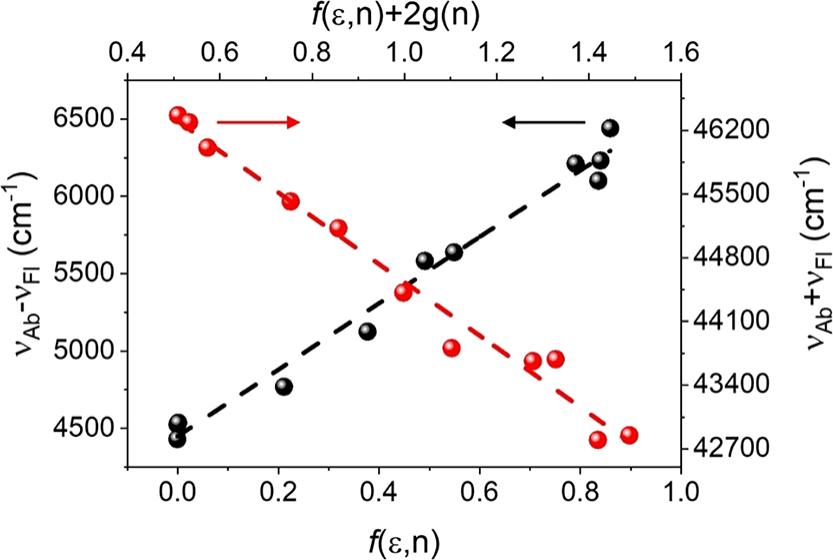
Plot of ν_Ab_ – ν_Fl_ vs *f*(ε,*n*) and ν_Ab_ + ν_Fl_ vs *f*(ε,*n*) + 2*g*(*n*) of the Ox-π,π-Ph(OMe) dye in different aprotic solvents.

Correlations between the absorption (ν_ab_) and fluorescence (ν_fl_) band shift of a spherical solute in solvents with different permittivity (ε) and refractive index (*n*), based on eqs S1 and S2 (see the Supporting Information), which result from the theory of Bilot and Kawski^39−43^ by employing the quantum–mechanical perturbation theory, give straight lines with the correlation coefficients being larger than 0.98, which indicate a good linearity for both *m*_1_ and *m*_2_ (Figure 6). The obtained slope values (2146.1 and 3601.6 cm^–1^ for *m*_1_ and *m*_2_, respectively) are rather high, indicating a significant variation of the dipole moment upon excitation. This suggests that the emission of the Ox-π,π-Ph(OMe) dye originates from a state that is more polar than the ground state.

According to the slopes and the Onsager cavity radius of the dye (5.48 Å) using eqs S5, S6, and S7, we got μ_g_ = 2.02 D and μ_e_ = 7.96 D and change in dipole moments Δμ = 5.94 D. The dipole moment change upon excitation corresponds to an intermolecular displacement of a charge, which is 1.24 Å. The applied extrapolation technique of the linear fit to the experimental data corresponds to the gaseous phase. Thus, the calculated dipole moments refer to an isolated molecule free from solvents that are characteristic for a given molecule.^43^ The calculated values indicate that the charge transfer accompanying excitation to the lowest excited singlet state results in the excited molecule having a greater dipole moment than the one in the ground state.^44^

The experimental data are supported by the theoretical calculation of the dipole moments presented in Figure 5 and Table S11. First, the dipole moment value in the ground (μ_GS_) and excited (μ_CT_) state increases as a function of solvent polarity for both *E* and *Z* isomers. The *E* isomer is characterized by a higher value of the CT excited-state dipole moment relative to the *Z* isomer (Δμ_GS_^*E* – *Z*^: 0.97 → 3.69 D from vacuum to water). However, for the *Z* isomer, a higher μ_GS_ is observed relative to *E* (Δμ_GS_^*Z* – *E*^: 0.88 → 1.02 D). At the same time, both isomers are characterized by a high polarity of the excited state (Δμ_CT – GS_), and the Δμ_CT – GS_ exceeds 6 D. Moreover, although the vector orientation is similar in both conformers (Figure 5), the presented observation indicates that the *E → Z* transitions occur with a substantial dipole moment change.

### Nonlinear Spectral Properties: Theoretical Aspects

According to the literature,^45−48^ a highly polarizable electron cloud is the main prerequisite for demonstrating the nonlinear optical (NLO) behavior of organic compounds. Such molecules typically have a π-conjugated framework, end-capped by electron-accepting and electron-donating groups to enhance the polarizability and hyperpolarizability.^49^

The polarizability and hyperpolarizability of the molecule irradiated with intense laser light giving the electric field are the subject of many studies in terms of understanding various linear and NLO properties. In particular, these studies include the interrelationship of the nonlinear optical properties with the electronic structure to design new multifunctional molecules. The calculated values for *E*/*Z* conformers are collected in Table 3 and Table S12 in the Supporting Information. For both *E* and *Z* conformers, the ⟨α⟩, Δα, and β_vec_ values increase monotonously with the solvent polarity. The *E* isomer is characterized by higher linear polarizability and first-order hyperpolarizability values. These observations suggest and at the same time confirm that the presence of the methoxy group is a factor maximizing the nonlinear response of the compound and the *E* conformation can be more efficient in the second harmonic generation (SHG) phenomenon.

**Table 3:** Theoretical Linear and Nonlinear Optical Properties of the Ox-π,π-Ph(OMe) Isomers

	GP		water	
	*Z*	*E*	*Z*	*E*
λ_Ab_^vert^ (nm)	417.58	443.17	427.03	471.19
λ_Ab_^cLR^ (nm)	420.43	447.31	420.00	453.18
λ_FL_^vert^ (nm)	474.16	480.71	550.31	541.03
μ_GS_ (D)	2.39	1.51	3.32	2.30
*x*	–2.30	1.51	–3.18	2.25
*y*	–0.37	–0.00	–0.04	–0.43
*z*	–0.51	–0.00	–0.097	0.02
μ_CT_ (D)	7.93	8.90	9.47	13.16
*x*	8.39	7.34	9.77	10.88
*y*	–4.49	0.99	–6.41	1.14
*z*	–0.96	0.00	–1.48	–0.04
Linear polarizabilities				
*xx*	386.86	610.96	523.05	858.05
*xy*	19.26	–23.61	31.58	–40.84
*yy*	231.93	247.04	342.06	358.91
*yz*	–21.02	1.08	–37.33	0.15
*zx*	8.74	–0.56	9.91	–1.53
*zz*	190.86	122.26	268.73	170.29
⟨α⟩ (a.u.)	269.89	326.75	377.00	462.42
Δα (a.u.)	180.27	440.11	229.46	616.19
First-order hyperpolarizabilities				
*xxx*	9.09	34.81	9.44	116.31
*xyy*	1.52	0.12	3.66	0.72
*xyz*	0.54	0.01	1.80	0.54
*yzz*	0.60	–0.52	1.76	–1.27
*yxx*	–5.02	3.93	–14.72	14.13
*yyy*	–1.61	1.17	–5.24	4.03
*yzz*	0.16	0.13	0.08	0.57
*zxx*	–1.32	0.01	–1.68	–0.57
*zyy*	–0.07	0.01	–0.79	0.08
*zzz*	0.40	0.00	1.08	0.04
β_vec_ (esu)	9.60	20.63	8.12	66.14

### Basic Spectroscopy in the Solid State

**Figure 7:**
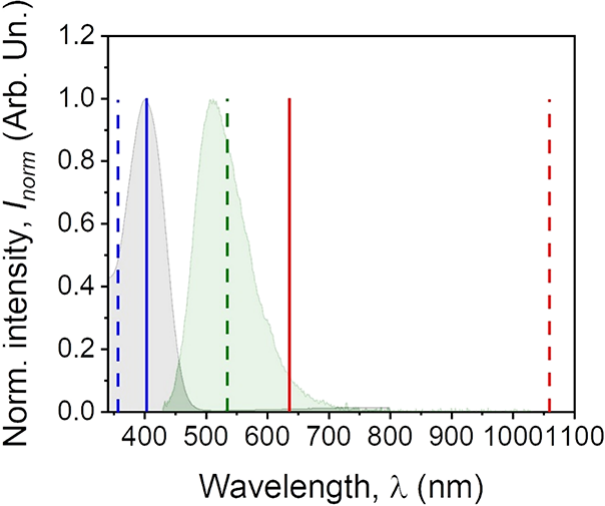
Absorption and fluorescence bands marked in gray and green regions, respectively. Solid blue and red lines denote the OKE experiment laser sources, which are pump (405 nm) and reference (633 nm) laser beams. Red, green, and blue dash lines represent the incident/fundamental beam (1064 nm) and its second (532 nm) and third (355 nm) harmonics of light, respectively.

In Figure 7, we have presented both absorption and fluorescence spectra of the Ox-π,π-Ph(OMe) chromophore collected from the PMMA-based thin film. The investigated molecular system absorbs electromagnetic wave from the UV range (∼300 nm) up to the visible region (∼500 nm), marked as a gray and down-field curve, and provides a significant fluorescence band (green curve and down-field) with the maximum centered at 510 nm (green emission). The observed Stokes shift is equal to 110 nm; moreover, two aforementioned bands represent symmetrical Gaussian shapes. In the same Figure 7, a set of experimentally introduced and observed signals was marked as well. Namely, by solid blue and red lines, we showed pumping and reference laser beams, respectively, implemented in a pump-probe laser setup where an all-optical switching phenomenon can be observed. Additionally, using dashed red, green, and blue lines, we have shown the fundamental beam as well as the second and third harmonics of light generated by the NLO medium for clarity. Based on the collected spectrum (Figure 7), it can be easily stated that in both NLO experiments, the provided reference and fundamental beams are out of the absorption resonance of the Ox-π,π-Ph(OMe) dye.

### Laser Spectroscopy in the Solid State

#### All-Optical Switching

**Figure 8:**
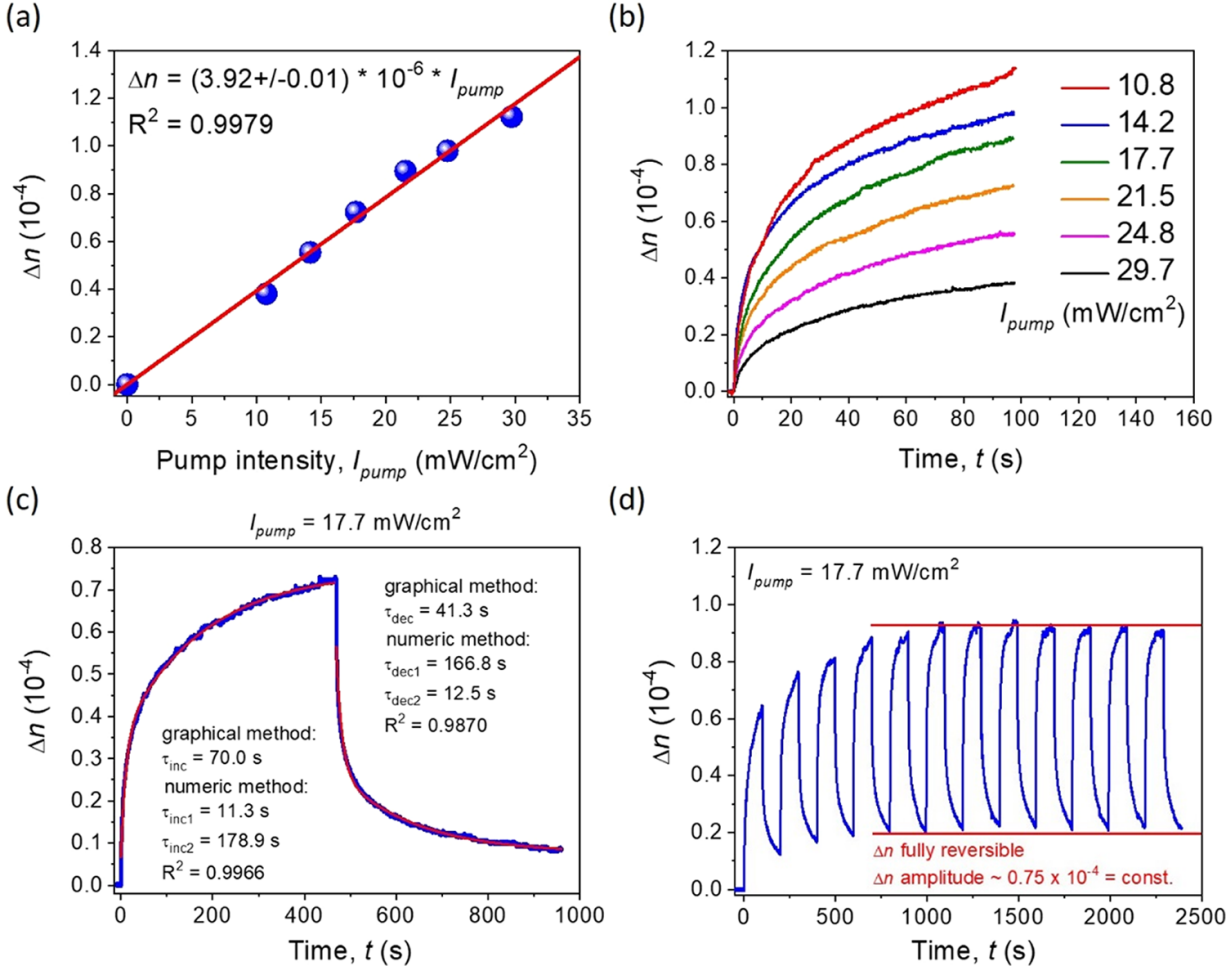
(a) Photoinduced birefringence vs pump beam intensity correlation and (b) kinetics of the Δ*n* signal over time using various *I*_pump_ values for static (total) OKE measurements. (c) High-quality singular measurement of Δ*n* UV-inducement and dark, thermodynamic relaxation with estimated time constants of these two processes using two methods and (d) multiple Δ*n* signal recording and erasing with full reversibility.

In Figure 8a, the linear correlation between photoinduced birefringence and the cause of the NLO effect, pump beam intensity, is presented. A value close to 1 of the coefficient of determination from the approximation function shown in the red line indicates an appropriate physical behavior, which represents the optical Kerr effect.^50,51^ From the same relation, a nonlinear refractive index (*n*_2_) value can be extracted. In the case of the Ox-π,π-Ph(OMe) NLO chromophore, it is equal to (3.92 ± 0.01) × 10^–10^ cm^2^/mW (in the SI unit system, *n*_2_ = (2.78 ± 0.01) × 10^–8^ m^2^/W). The experimental points taken into account for the correlation shown in Figure 8a originate from the Δ*n* kinetics measurement presented in Figure 8b after c.a. 100 s. Sets of increasing signals, which represent rising optical anisotropy, were shown according to the increase pump beam intensity. The NLO reversible response was recorded also in a singular high-quality measurement (Figure 8c). In here, where Δ*n* was shown as a signal increase (UV laser light was applied, up to ∼480 s), and after the moment, when the darkness, thermodynamic conditions were present (UV light in the ″OFF″ state) and the signal started to decrease. Two approaches were utilized to approximate experimental curves (Δ*n* inducement and vanishing) as mono- and biexponential by graphical and numerical methodologies, respectively. The first mentioned time constant values of increase and decrease fragments were defined to be 70.0 and 41.3 s, respectively. It means that thermodynamic relaxation is an almost two times faster process than that artificially caused by UV light photoinduced birefringence. The other tendency is shown by time constant values estimated by the numerical method. Namely, the very first moment of both UV light irradiation as well as its deactivation is fast (τ_inc1_ = 11.3 s and τ_dec1_ = 12.5 s), whereas the second part of the same processes is much slower by about 2 orders of magnitude, giving the following numbers: τ_inc2_ = 178.9 s and τ_dec2_ = 166.8 s, respectively. Typically, in the observed optical Kerr effect, the monoexponential approximation function is used:^50,51^ however, in the case of the Ox-π,π-Ph(OMe) NLO chromophore, the rule may be different for a structural reason, namely, an existence of two π-bonds localized in the central part of the molecule. One of them is partially blocked by the close heteroatomic ring neighborhood, while the second one is surrounded just by hydrogen atoms, providing a much higher flexibility in this region (Figure 1). Thus, it can be responsible for two various kinetics of the observed UV-induced photoisomerization as well as thermodynamic processes. Additionally, the high signal stability and reversibility of the remote-controlled photoinduced birefringence are shown in Figure 8d. Only the first three UV light ON/OFF cycles characterize the increasing value of the generated Δ*n* signal. The next modulations practically reach the same level of obtained NLO response continuously (Δ*n* amplitude ∼0.75 × 10^–4^), which demonstrates a highly stable and responsive organic system controlled fully by light (all-optical switching).

**Figure 9:**
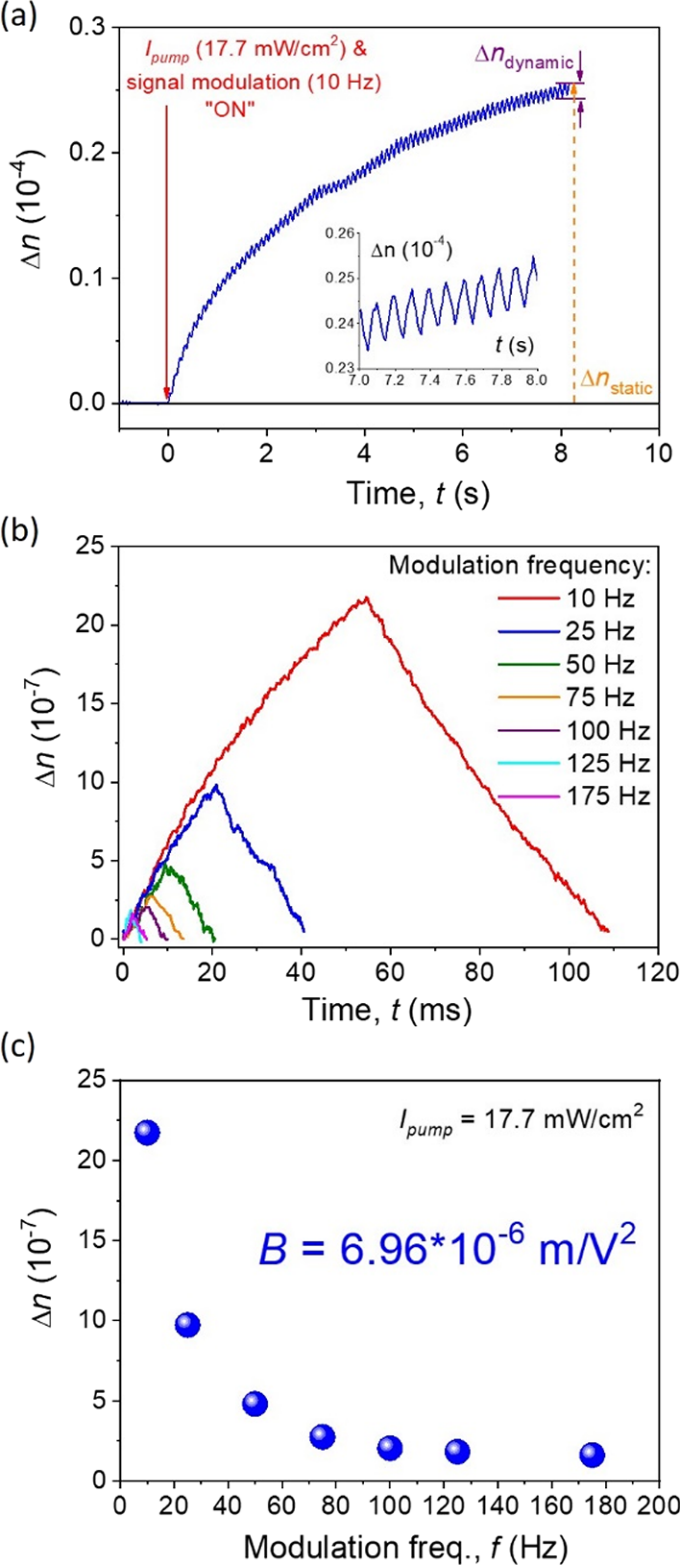
Photoinduced birefringence kinetics in the long-term measurement with applied external signal triggering. Inset shows trans–cis–trans molecular transformations (a). OKE dynamic signal presentation with various external triggering (b) and Δ*n* maxima (signal amplitude) in modulation frequency function (c).

Based on the theory of the all-optical switching phenomenon observed in the optical Kerr effect,^50,51^ the macroscopic optical anisotropy generation originates from multiple *E-Z-E* (trans–cis–trans) isomerization cycles, which were already confirmed by quantum chemical calculations results (Figure 5). All these spatial structural changes are feasible thanks to the presence of at least one free π-bond in the photoresponsive system. The efficiency of UV-light absorption is controlled by a simple rule, defined as *P* = cos^2^(α), where *P* means the probability of photon absorption and α denotes the angle between the polarization direction of the provided laser light and long axis of the molecule (typically, the same direction as the π-bond embedded into the structure). Therefore, the absorption as well as photoisomerization cycles will take place up to the moment when optical anisotropy achieves a photostationary state. Namely, the maximum population of molecules will be oriented perpendicularly toward applied linearly polarized UV light. In Figure 9a, we present the photoinduced birefringence signal, which was collected under a particular value of pump beam intensity (*I*_pump_ = 17.1 mW/cm^2^) and external signal modulation (*f* = 10 Hz) at the same time. In that way, it was possible to show what is the dynamic component’s (multiple *E-Z-E* isomerization) participation to the total Δ*n* value. The total (static) photoinduced birefringence is marked in yellow, whereas its dynamic component is shown in violet color in Figure 9a and presented as a signal modulation between the seventh and eighth measurement second in its inset.

Afterward, the dynamic part of estimated photoinduced birefringence characteristics is presented in Figure 9b,c. Figure 9b shows a set of singular photoinduced isomerization trans–cis–trans, where the Δ*n* signal amplitude increases and decreases, respectively, for selected frequency modulations in the range of 10–175 Hz. It is clearly shown that the needed time (∼110 ms) and amplitude magnitude (2.2 × 10^–6^) are the highest for the lowest value of external signal modulation (10 Hz). Such tendency is exponentially decreasing along the higher-frequency modulations up to almost 200 Hz, which is presented in Figure 9c. Also there, a Kerr constant is presented, which is 6.96 × 10^–6^ m/V^2^. Finally, the third-order nonlinear optical susceptibility value was determined using all experimental achievements and led us to define χ^(3)^ as 2.18 × 10^–10^ in the SI unit system (2.55 × 10^–8^ cm^2^/W in cgs).

By making a comparison with other, similar low-molecular NLO chromophores existing in polymeric systems, it can be stated that the presented results are in accordance with already achieved developments.^21,52−54^ Going into details, the obtained photoinduced birefringence for the Ox-π,π-Ph(OMe) NLO active system is in the same order of magnitude as its previously investigated derivative (Ox-π,π-Ph/PMMA, structure shorter on the -OMe moiety). The cited organic system characterizes a Δ*n* value equal to 5.3 × 10^–4^ as the reached maximum for *I*_pump_ ∼ 50 mW/cm^2^ and about 3.5 × 10^–4^ when *I*_pump_ ∼ 10 mW/cm^2^^24^ (the latter constitutes a more reliable comparison with respect to the experimental environment applied then and during these investigations). Subsequently, other organic NLO dyes, like pyrazolone,^21^ pyrazoline,^52^ or thiophene^53^ derivatives, represent a Δ*n* parameter on the similar level of photoinduced birefringence, i.e., equal to 5.5 × 10^–4^, 1.0 × 10^–4^, and 1.5 × 10^–4^, respectively. However, in all these cases, the pump intensity used to achieve such magnitude of photoinduced values was much higher than in the current situation (or in pyrazolone or oxazolone derivatives). Meanwhile, if comparing azobenzene derivatives like DR1, CPND-5, SR7B, or S3 NLO chromophores, Δ*n* values are rather varied (from 1.4 × 10^–3^ up to the 4.0 × 10^–3^).^54^ All estimated NLO parameters compared with available literature data are presented in Table 4.

**Table 4:** Summary of the Nonlinear Optical Parameters Based on the All-Optical Switching Experiment for Various Organic-Based NLO Active Systems

compound (acronym)	Δ*n* (×10^–4^)	*n*_2_ (m^2^/W)	χ^(3)^ (m^2^/V^2^)	*B* (m/V^2^)
**Ox-π,π-Ph(OMe)**^a^	**1.2**	**(2.78 ± 0.01) × 10^–8^**	**2.18 × 10^–10^**	**6.96 × 10^–6^**
Ox-π,π-Ph^b^	5.3	2.89 × 10^–8^	2.3 × 10^–10^	2.92 × 10^–5^
DCNP^c^	4.0	2.0 × 10^–9^	1.8 × 10^–11^	4.1 × 10^–7^
PY-*p*NO_2_^c^	1.0	6.0 × 10^–10^	5.6 × 10^–12^	3.9 × 10^–7^
Th-*p*NO_2_^c^	1.5	3.0 × 10^–8^	2.4 × 10^–10^	3.6 × 10^–6^
DR1^d^	35			
CPND5^d^	14			
S3^d^	14			

a Current contribution.

b Ref (24).

c Ref (55).

d Ref (54).

#### Harmonics of Light Generation

**Figure 10:**
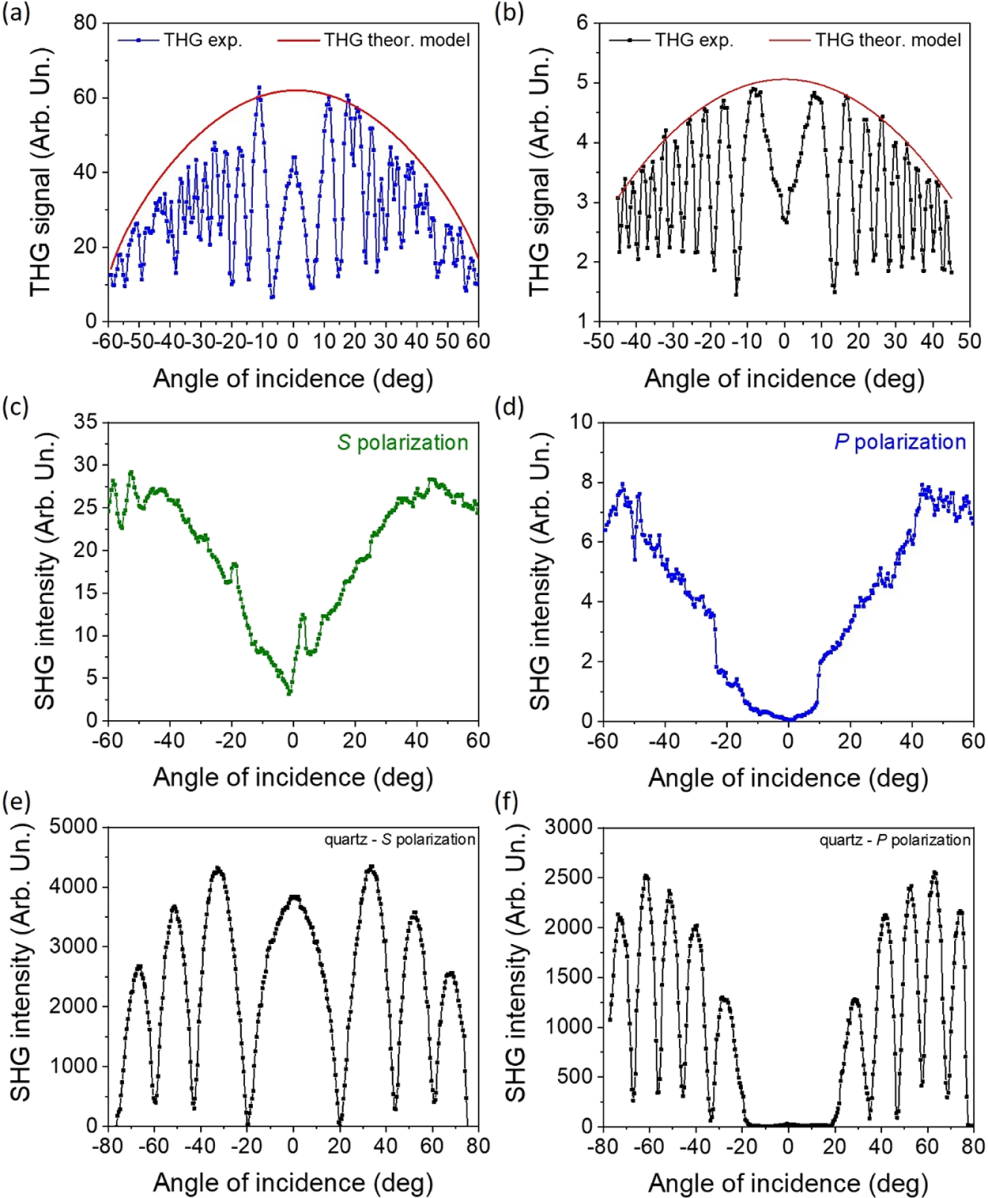
THG signals as Maker fringes for the (a) Ox-π,π-Ph(OMe) dye and (b) silica, respectively. SHG signals for (c, d) the NLO chromophore and (e, f) quartz in both (c, e) *S* and (d, f) *P* polarization configurations, respectively.

In Figure 10a,b, typical Maker fringe signals collected from the investigated NLO chromophore as well as the reference material (silica) are presented, respectively. It is clearly visible how the generated THG signal is sensitive due to the angle between the fundamental laser beam and sample surface. Such rotation proves and shows dominant signals localized symmetrically in both sides, with the most intense bands close to the normal direction and decreasing ones for the higher rotation angles. Based on the collected spectra and applying the Kubodera and Kobayashi theoretical model^56^ (marked as the red curve in Figure 10a,b), the χ_elec._^(3)^ component value defined for the Ox-π,π-Ph(OMe) NLO chromophore was (3.38 ± 0.02) × 10^–22^ m^2^/V^2^. The same parameter was already estimated for the reference material and is equal to 2.0 × 10^–22^ m^2^/V^2^.^57,58^ If comparing similar chemical structures for which the third-order NLO susceptibility parameter was defined, the current results place the Ox-π,π-Ph(OMe) system among the active and significant higher harmonic of light (THG) generators. For instance, the oxazolone derivative cited before is characterized by χ_elec._^(3)^ equal to 6.0 × 10^–22^ m^2^/V^2^,^24^ which represents the same order of magnitude of the experimental achievement. However, there are known systems where the χ_elec._^(3)^ parameter is even higher (i.e., pyrazoline derivatives molecules, DCNP 11.0 × 10^–22^ m^2^/V^2^ or PY-*p*NO_2_ 81.4 × 10^–22^ m^2^/V^2^, respectively)^59^ or lower (i.e., TTF-based NLO chromophores characterized by χ_elec._^(3)^ parameter less than 0.5 × 10^–22^ m^2^/V^2^).^60^ Such observation makes materials engineering even more attractive and useful because based on the chemical structure pattern, it is possible to influence straightforwardly of the output physical, in particular, spectroscopic properties of the compounds. (Table 5).

**Table 5:** Fourth- and Third-Order Nonlinear Optical Tensor Values for the Selected Organic Systems

compound (acronym)	χ_elec._^(3)^ (m^2^/V^2^) × 10^–22^	χ^(2)^*S* polarization (pm/V)	χ^(2)^*P* polarization (pm/V)
**Ox-π,π-Ph(OMe)**^a^	**3.38 ± 0.02**	**0.025 ± 0.001**	**0.056 ± 0.001**
silica (THG reference)^b^	2.00		
quartz (SHG reference)^c^		1.00	1.00
Ox-π,π-Ph^d^	6.0	0.05	0.14
DCNP^e^^*,*^^f^	11.0	11.0	5.5
PY-*p*NO_2_^e^^*,*^^f^	81.4	6.86	3.43
TTF derivative_1^g^	0.37	6.80	8.00
TTF derivative_2^g^	0.20	0.94	2.72

a Current contribution.

b Refs (57) and^58^.

c Ref (61).

d Ref (24).

e Ref (59).

f Ref (64).

g Ref (60).

In Figure 10c–e, there are presented Maker fringes representing the second harmonic of light signal collected for the investigated sample (Figure 10c,d) and reference material (*Y*-cut quartz slab, Figure 10e,f) in *S* (parallel, Figure 10c,e) and *P* (perpendicular, Figure 10d,f) polarization configuration, respectively. Such geometrical changes introduced to the experimental setup allowed us to define whether the investigated NLO chromophore is sensitive (or not) to various spatial fundamental laser beam configurations. Indeed, when *S* polarization was used (the same as the laser output), the SHG magnitude was higher than that in the *P* one (oriented orthogonally with respect to the laser source) by about 1 order of magnitude (Figure 10c,d). There is no doubt that SHG signal modulation was significant and easily observed in both laser polarization configurations, albeit its influence on the χ^(2)^ value is almost negligible. The second-order NLO susceptibility value was defined as 0.025 ± 0.001 and 0.056 ± 0.001 pm/V for *S* and *P* experimental setup configurations, respectively. Together with the already defined χ^(2)^ parameter for the quartz slab used as the reference material, which is 1.00 pm/V,^61^ it seems that the Ox-π,π-Ph(OMe) NLO active medium can serve as a very precise SHG sensor that is also sensitive in a spatial manner.^61−63^ When discussing the obtained χ^(2)^ parameter for the investigated system together with the already known pyrazolone-based NLO system, it is quite similar in generating a low-value SHG signal; however, its magnitude is significantly different and higher (doubled) for the *P* polarization configuration than the *S* one (Table 5). Anyhow, such trend where one of the laser polarization configurations can influence the output SHG signal magnitude is more significant for other group of compounds, i.e., TTF-based NLO chromophores. In that case, χ^(2)^ in the *P* configuration is even 1 order of magnitude higher than that in the *S* one (i.e., 2.72 pm/V together with 0.94 pm/V; Table 5).^60^ Furthermore, in the literature, there are known compounds, i.e., stilbene- or azobenzene-based derivatives, which generate even higher second harmonic of light signals and at the same time are characterized by a higher value of the third-order NLO tensor, χ^(2)^, namely, DCNP or PY-*p*NO_2_ molecules^64^ or disperse red 1.^65^

## Conclusions

In this contribution, we have shown a chemically modified oxazolone dye with appealing optical and nonlinear optical properties, which can be easily implemented into spectroscopy and electro-optical devices. Starting from synthesis, we have presented the ease of fabrication and further manipulation, which are desirable from the application’s point of view. Quantum chemical calculations confirmed the existence of two stable, spatially different forms, which are responsible for significantly different spectroscopic responses. The internal electron charge transfer proven by a theoretical approach confirms the efficient movement of the negative carriers, which causes a significant THG signal (χ_elec._^(3)^ value far from 0). Moreover, by applying the appropriate thermoelectrical prefabrication process, it was feasible to break the internal symmetry and finally induce the SHG signal, and it was also sensitive to spatial changes applied in the experimental setup environment. Then, by means of implementing low-energy CW laser emission, it was possible to modulate the refractive index value effortlessly. Using a typical pump-probe setup, we have proven that the Ox-π,π-Ph(OMe) NLO chromophore can characterize both the optical isotropy and anisotropy of the *n* coefficient, which paved the way to construct logical gates working on 0–1 modules. Especially, the remote-controlled two-distinguishable-states switching process is stable over time with the same signal amplitude. Such revelations show undoubtedly that oxazolone derivatives can play a pivotal role as useful and efficient spectroscopy tools for various applications.

## Experimental Section

### Materials and Methods

The reagents for the synthesis, i.e., hippuric acid, 2-methoxycinnamaldehyde, sodium acetate (anhyd.), and acetic anhydride, as well as all solvents (spectroscopic grade, Table S1 in the Supplementary Information), were purchased from either Aldrich Chemical Co. or Chemat Co. Poland, and they were used as obtained.

The oxazolone dye, 4-[3-(2-methoxyphenyl)prop-2-enylidene]-2-phenyl-1,3-oxazol-5-one, was synthesized by the method described in the literature^66,67^ using hippuric acid (**1**; 0.5 g, 2.8 mmol, 1 equiv), 2-methoxycinnamaldehyde (**2**; 0.45 g, 2.8 mmol, 1 equiv), sodium acetate (0.23 g, 2.8 mmol, 1 equiv), and acetic anhydride (1.3 mL, 1.43 g, 14 mmol, 5 equiv).

Hippuric acid (**1**) in acetic anhydride was heated at 50–60 °C until a clear yellow solution of compound **1a** was obtained (30 min). Then, 2-methoxycinnamaldehyde (**2**) and anhydrous sodium acetate were added to the solution. The reaction mixture was refluxed with stirring for 4 h. After cooling to room temperature, the mixture was diluted with anhydrous ethanol (5 mL, 4.4 g, 95 mmol, 34 equiv) and kept below 0 °C for 20 min. The precipitate was filtered off under suction, washed with cooled ethanol and then with hot distilled water, and dried. The crude product was purified by flash chromatography using chloroform as the eluent to give an orange crystalline solid. Yield 41% (0.35 g), mp: 151–152 °C, TOF MS (ESI) *m*/*z*: [M + H]^+^ calcd for C_19_H_16_NO_3_ m/e 306.1130 (100%), 307.1163 (20.5%), 308.1197 (2.0%); found 306.1129, 307.1155, 308.1197.

^1^H NMR (400 MHz, DMSO-*d*_6_) δ 8.09–8.07 (2d, *J* = 8 Hz, 2H, Ph), 7.72 (m, 2H, Ph), 7.68–7.65 (d, *J* = 12 Hz, 1H, =CH−), 7.64–7.62 (d, *J* = 8 Hz, 2H, Ph), 7.61 (m, 1H, Ph), 7.42 (t, 1H, =CH−), 7.33–7.30 (d, *J* = 12.0 Hz, 1H, =CH−), 7.13–7.11 (d, *J* = 8.0 Hz, 1H, Ph), 7.04 (t, 1H, Ph), 3.91 (s, 3H, OCH_3_);

^13^C{^1^H} NMR (100 MHz, DMSO-*d*_6_) δ 166.6, 161.5, 158.2, 139.9, 133.5, 134.0, 133.8, 132.2, 129.8, 128.7, 128.2, 125.8, 124.7, 124.2, 121.4, 112.3, 56.2;

^15^N NMR (40 MHz, DMSO-*d*_6_) δ 238.07.

IR (KBr, cm^–1^) 3019, 2985, 2837, 1781, 1645, 1592, 1555, 1489, 1468, 1451, 1358, 1327, 1297, 1254, 1162, 1027, 976, 876, 777, 762, 745, 696.

Structural assignments were made with additional information from gCOSY, gHSQC, and gHMBC experiments. The NMR and IR spectra are provided in the Supporting Information.

### Spectral Measurements

The 1D and 2D NMR spectra were recorded on a Bruker Ascend 400 spectrometer with a resolution of 400 and 100 MHz for ^1^H and ^13^C spectra, respectively (software Bruker Top Spin 2.1). Tetramethylsilane (TMS) was used as an internal standard for calibrating the chemical shift. The measurements were performed in dimethylsulfoxide (DMSO-*d*_6_). The IR spectra were recorded on a Bruker Vector 22 FT-IR spectrophotometer (Germany) in the range of 400–4500 cm^–1^ by the KBr pellet technique. Melting point (m.p.) values were determined on a Boëthius apparatus (Vernon Hills, United States) and were not calibrated. The TOF MS spectrum was recorded on a Waters LCT Premier XE mass spectrometer with ESI ionization. The HPLC analysis was done by a Waters Breeze 2 HPLC system equipped with a UV–vis detector (detection wavelength was 420 nm), binary HPLC pump, and a Symmetry C18 column (3.5 μm, 4.6 × 75 mm). Separation was conducted under isocratic conditions with 1.0 mL/min flow rate at r.t., 10 μL injection volume, and HPLC-grade acetonitrile as a mobile phase.

The UV–vis absorption spectra from solutions were recorded at room temperature on a UV–vis Multispec-1501 spectrophotometer from Shimadzu, whereas the fluorescence spectra were determined using an F-7100 spectrometer from Hitachi. The solution concentration was ca. 10^–5^ and 10^–6^ M for absorption and fluorescence measurements, respectively.

The fluorescence decay curves were recorded on a single-photon counting system (FLS920P Spectrometer) from Edinburgh Instruments. The sample was excited by a picosecond diode pulsed laser source with a high repetition rate (pulse ca. 55 ps at 375 nm). Photons emitted by the sample were detected with a fast microchannel plate photodetector, and the time with respect to the excitation pulse was measured. The fluorescence decay curves were analyzed using the Fast program, and they were fitted as sums of two exponentials. The average lifetime, τ_av_, was calculated as τ_av_ = (Σ_*i*_α_*i*_τ*_i_)*/(Σ_*i*_α*_i_)*, where α_*i*_ and τ_*i*_ are the amplitudes and lifetimes, accordingly. The compounds were studied at the concentration needed to provide an absorbance of 0.2–0.3 in a 10 mm cell at 375 nm.

The fluorescence quantum yield (FQY) was calculated by the comparative method using Coumarin 153 in an ethanol solvent (ϕ_ref_ = 0.38^68^) as reference. The absorbance (*A*) of both the dye and reference solution at an excitation wavelength (380–404 nm) was ca. 0.1.

The solvent effect on the spectral properties of the tested dye was analyzed by applying multilinear correlation based on the four-parameter Catalán^34^ solvent scale (Table S1 in the Supporting Information) and the Bakhshiev^69^ as well as Bilot and Kawski^39−42^ theories including the Onsager description of nonspecific electrostatic solute–solvent interactions.

### Computational Details

All geometrical parameters of the investigated molecules in their ground (*S*_GS_) and excited states (*S*_CT_) were calculated using the density functional theory (DFT) approach implemented in the Gaussian 09 program package^70^ with the TIGHT threshold option and PBE0/6-311++G(d,p) basis set. To verify that all the structures correspond to the minima on the potential energy surface, an analysis of Hessians was performed. The electronic properties were characterized by computations of the vertical absorption and emission spectra, which were obtained using the time-dependent density functional theory (TDDFT/PBE0)^71^ and by including the state-specific (SS) corrected linear response (cLR) approach.^72^ All spectroscopic calculations were performed using several different functionals, namely, the standard-hybrid PBE0^73,74^ functionals. The dipole moments and polarities of the charge-transfer state (CT) were evaluated by numerical differentiation of the excitation energies (*E*) in the presence of an electric field *F* of 0.001 a.u. strength:^75^
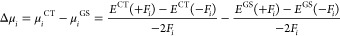
2where *F_i_* corresponds to the electric field applied along the Cartesian direction and *i* and μ_*i*_ are the *i*th Cartesian component of the electric dipole moment, respectively. The isotropic average polarizability (⟨α⟩), polarizability anisotropy (Δα), and first-order hyperpolarizability (β_vec_) were determined based on the Gaussian 09 program and were defined as follows:
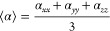
3
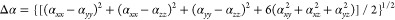
4
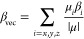
5where β_*i*_(*i* = *x*, *y*, *z*) is given by .

The density differences were obtained at the PBE0/6-311++G(d,p) level represented with a contour threshold of 0.02 a.u. The charge-transfer distance (*D*_CT_) and the amount of transferred charge (*q*_CT_) have been determined following Le Bahers’s procedure.^76^ The solvent effect on the linear and nonlinear optical properties has been taken into account using the Integral Equation Formalism for the Polarizable Continuum Model (IEF-PCM).^77,78^

### Solid-State Experiments

Samples in the solid state were prepared as follows: two components in powder forms being in 2% dry weight to weight ratio between the guest (Ox-π,π-Ph(OMe) dye) and host (poly(methyl methacrylate), PMMA) were dissolved in dichloromethane (DCM). When the solution was homogeneous, it was deposited on the pure glass substrate using the spin-coating (2000 rpm, 1 min, 400 μL) or drop-casting technique (400 μL, drying process under DCM atmosphere by 1 day) depending on the dedicated experimental needs. After complete solvent evaporation, such prepared thin films were ready for further studies.

Absorption spectra of the thin films were collected using a JASCO V-670 spectrometer, whereas their fluorescence spectra were recorded with the help of Fluoromax-4 HORIBA.

Thickness values of each thin film were measured in several randomly selected points on the sample surface but localized mainly in the central part, where all kinds of laser spectroscopy measurements were performed. In here, a DEKTAK 3 profilometer was used.

The optical Kerr effect (OKE) phenomenon was investigated in a typical pump-probe laser setup. The diode pumped solid-state (DPSS) lasers with emission at 405 and 638 nm were introduced as pump and probe beams, respectively. All principles defining the OKE experiment and comprehensive characterization of the third-order NLO effects (including all-optical switching phenomena) are described in the literature.^50,51^ Here are just some important details. Briefly, the utilized laser lines (pump and reference ones) intercrossed on the sample surface, creating a light spot with a 3 mm diameter. The thin film was localized between two polarizers, turned to each other by 90°. The NLO response was read out using a silicon photodiode mounted behind a cross-polarizer system and prevented by a red filter, which cut off the pumping laser line. Additionally, to maximize the observed effect, the linearly polarized UV light was oriented to be turned by 45° with respect to the polarization direction of the reference beam.^50,51^ For the optical Kerr effect investigation, the measured and averaged sample thickness was 24.5 μm.

To achieve a complementary investigation of nonlinear optical features of the Ox-π,π-Ph(OMe) chromophore, the ability of higher harmonics of light generation was experimentally checked. Using a comparable technique based on the Maker fringe measurements,^79^ the second-order NLO susceptibility value (χ^(2)^) and electronic component of χ_elec._^(3)^ were estimated. The experimental setup describing introduced electro-optical elements is described in the literature;^80^ however, several important details will be provided also in this contribution. The picosecond pulsed laser source was utilized to provide the fundamental beam with a repetition rate equal to 10 Hz (*t* = 30 ps) and generated wavelength at 1064 nm. Input and output signals were monitored by electro-optical controllers. The initial beam was illuminating the sample surface, which was rotated from −60/–80° up to +60/+80° depending on the observed effect and type of sample (reference material or thin polymeric film). For the THG experiments, a spin-coated sample was used (*d*_THG_ = 1.72 μm), whereas for the SHG ones, a drop-casted layer was prepared (*d*_SHG_ = 13.11 μm). Such approach was motivated by a particular nature of the performed experiments. Since the investigated Ox-π,π-Ph(OMe) NLO chromophore characterizes centrosymmetry, it was necessary to use an additional procedure for breaking the symmetry, namely, the corona poling technique.^81,82^ For a more efficient molecular ordering using thermoelectric methods, the used sample thickness was increased upon above of 10 μm. If considering the second harmonic generation investigation, as it was mentioned before, the drop-casted layer was transformed according to the corona poling procedure. The sample was treated by a higher temperature (100 °C) and applied DC voltage (8 kV) for 10 min. After that time, with the still applied external electric field, the thin film decreased its temperature and sustained its broken symmetry, which enabled further SHG studies. To analyze experimental results on both THG and SHG experiments, the Kubodera and Kobayashi^56^ as well as Lee^83^ theoretical models were introduced, respectively.

## Supporting Information Available

Solvent parameters, photophysical data, absorption fluorescence and fluorescence excitation spectra, calculated values of excitation energies and oscillator strengths, and plots of orbital contour surfaces (PDF)
