# Supplementary material for: A Modified Oxazolone Dye Dedicated to Spectroscopy and Optoelectronics
Source: J Org Chem. 2022 May 19;87(11):7319–32. doi: 10.1021/acs.joc.2c00500 (PMC9171828; doi:10.1021/acs.joc.2c00500)
Supplement: Supplementary file 1 — jo2c00500_si_001.pdf [file jo2c00500_si_001.pdf]

Supporting Information

A modified oxazolone dye dedicated to spectroscopy and optoelectronics

Adam Szukalski,*,† Przemysław Krawczyk,‡ Bouchta Sahraoui, Faustyna Rosińska,§,1 Beata Jędrzejewska*,§

†Wroclaw University of Science and Technology, Faculty of Chemistry, Wyb. Wyspiańskiego 27, 50-370 Wrocław, Poland

‡Nicolaus Copernicus University, Collegium Medicum, Faculty of Pharmacy, Kurpińskiego 5, 85-950 Bydgoszcz, Poland

Laboratoire MOLTECH-Anjou, Université d'Angers, UFR Sciences, UMR 6200, CNRS, 2 Bd. Lavoisier, 49045, Angers Cedex, France

§Bydgoszcz University of Science and Technology, Faculty of Chemical Technology and Engineering, Seminaryjna 3, 85-326 Bydgoszcz, Poland

Corresponding authors e-mail addresses: adam.szukalski@pwr.edu.pl; beata@pbs.edu.pl

Table of contents	Page
^{1}H NMR spectra	S3
^{13}C NMR spectra	S4-S5
COSY_^{1}H NMR spectra	S6
HMBC_^{13}C NMR spectra	S7
HMBC_^{15}N NMR spectra	S8-S9
IR spectra	S10
HPLC	S11
ESI-HRMS spectrum	S11-S12
S1. Dipole moments determination	S12-S13
Atom numbering scheme – Figure S1	S13
Solvent parameters – Table S1	S14
Structural parameters of the *E* isomer in the ground state – Table S2	S15
Structural parameters of the *Z* isomer in the ground state – Table S3	S16

Changes in absorption and fluorescence spectra after one week of storing the diluted solution at room temperature – Figure S2	S17
Absorption and fluorescence spectra of Ox-π,π-Ph(OMe) in solvents tested – Figure S3	S17
Fluorescence excitation and fluorescence spectra of Ox-π,π-Ph(OMe) – Figure S4	S18
S2. Spectral properties	S18
The vertical excitation energies – Table S4	S19
The cLR corrected excitation energies – Table S5	S20
The vertical de-excitation energies – Table S6	S20
Fluorescence lifetimes data – Table S7	S21
Radiative and nonradiative rate constants – Table S8	S22
S3. Electrochemical properties	S23
Density difference plots – Figure S5	S23-S24
The frontier orbital energies in selected solvents – Table S9	S25
CT parameters for the bright low-lying excited state of Ox-π,π-Ph(OMe) – Table S10	S26
Calculated values of dipole moments for the ground and CT excited state – Table S11	S26
Nonlinear properties of Ox-π,π-Ph(OMe) isomers – Table S12	S27
S4. Cartesian coordinates of optimized structures	S27-53
The number of imaginary frequencies for structures in the ground state – Table S13	S54
Computed total energies of optimized structures in the ground state. Values are given in a.u. – Table S14	S54
The number of imaginary frequencies for the transition state structure – Table S15	S55
Computed total energies of the transition state. Values are given in a.u. – Table S16	S55
List of references	S55

^{1}H NMR spectrum of Ox-π,π-Ph(OMe) in DMSO-d_6 (400 MHz)

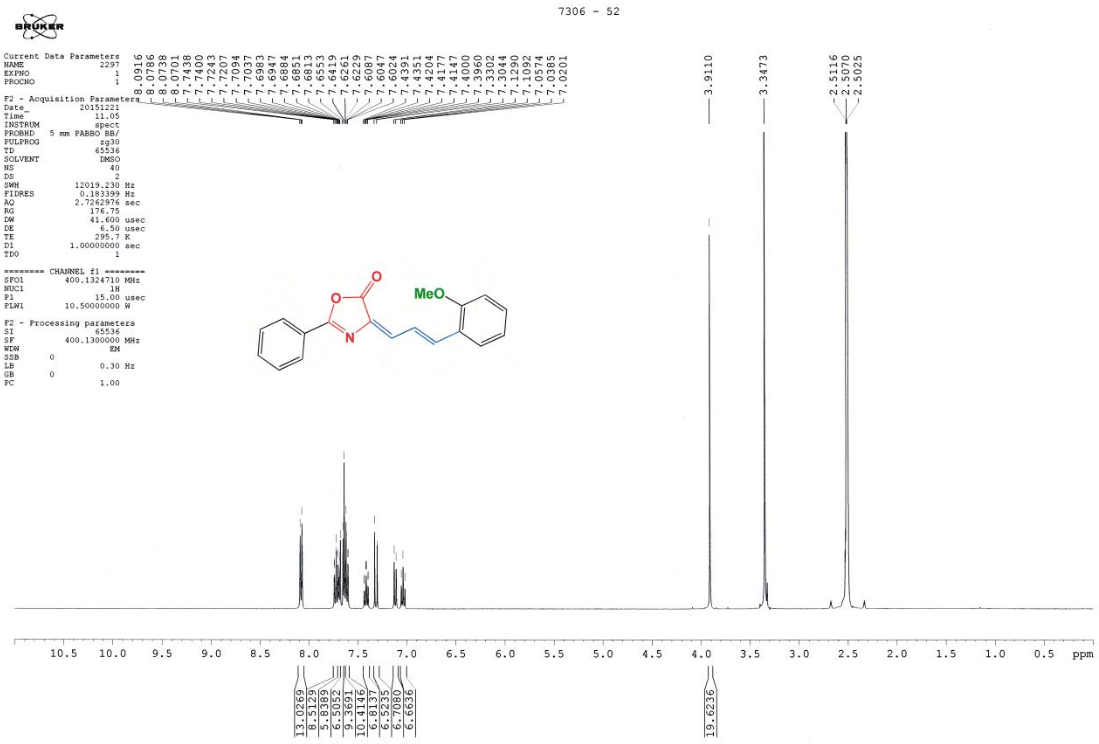

Enlarged spectrum in the range of 6-9 ppm.

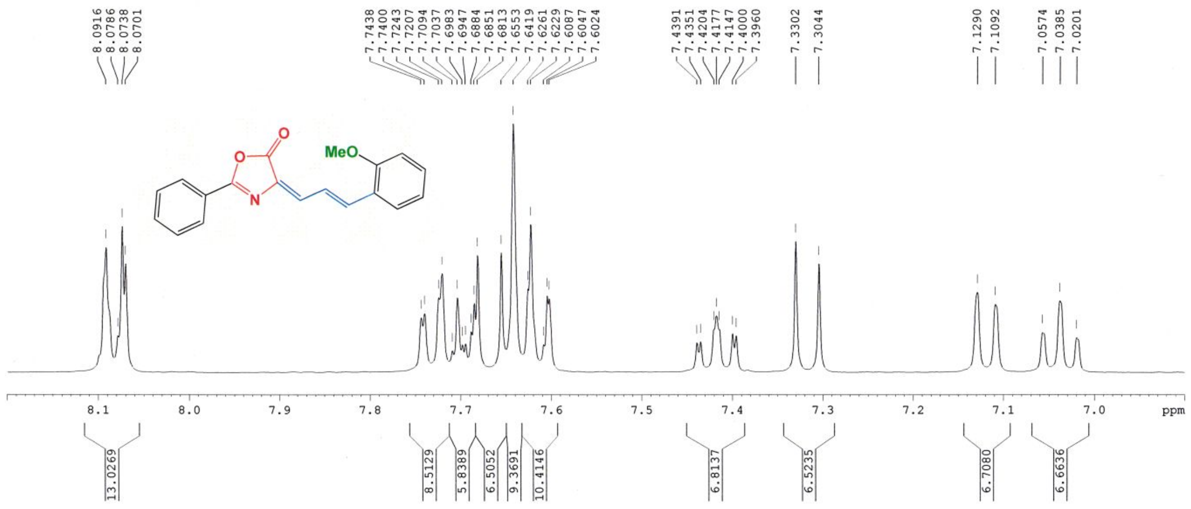

$^{13}C\{^1H\}$ NMR spectrum of Ox-π,π-Ph(OMe) in DMSO-d_6 (100 MHz)

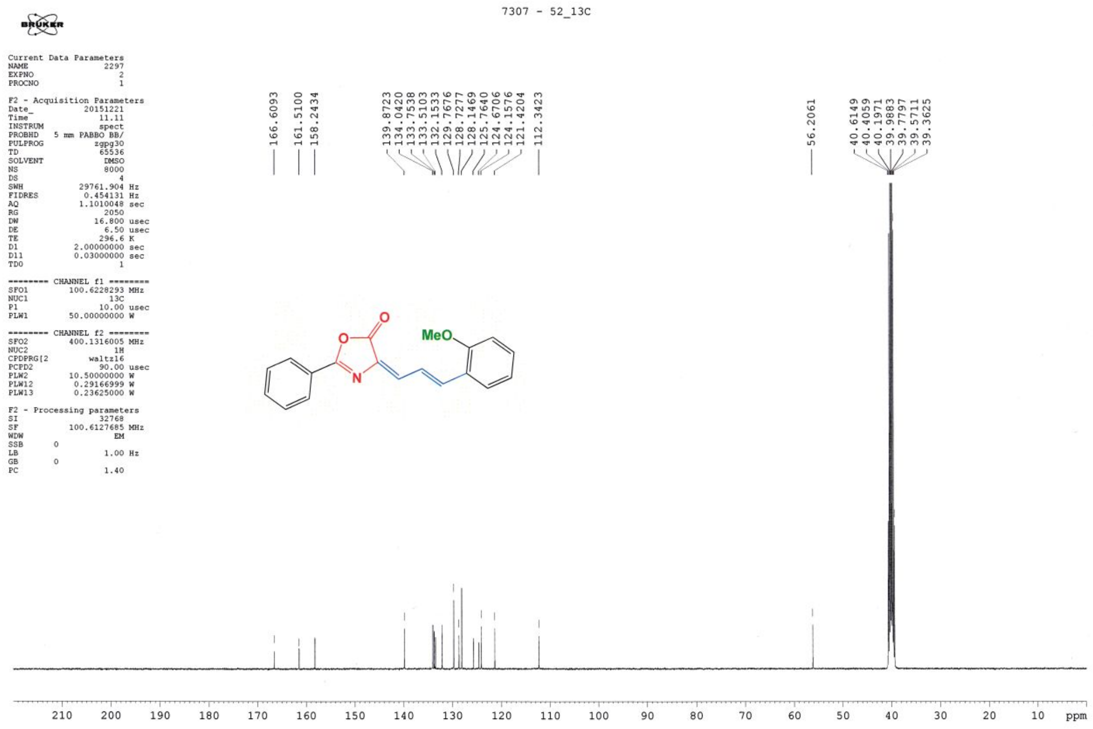

Enlarged spectrum in the range of 100-170 ppm.

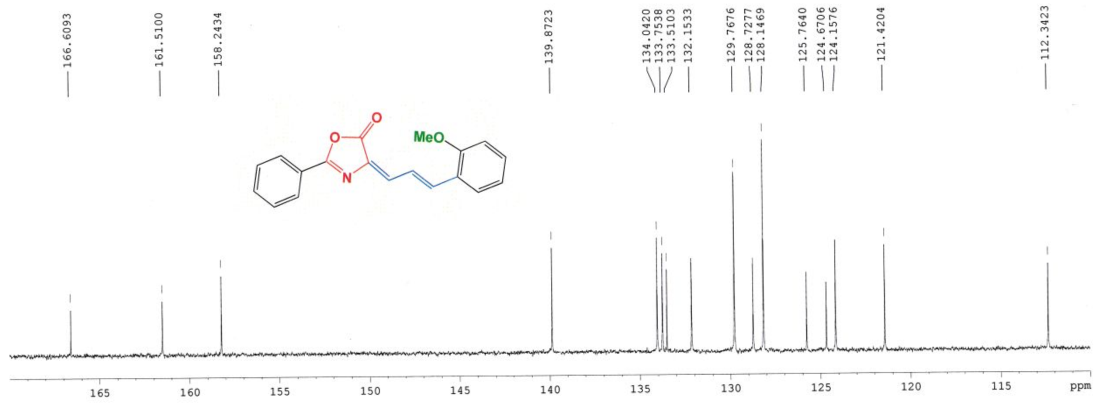

$^{13}C\{^{1}H\}$ NMR spectrum of Ox-π,π-Ph(OMe) dept in DMSO-d_6 (100 MHz)

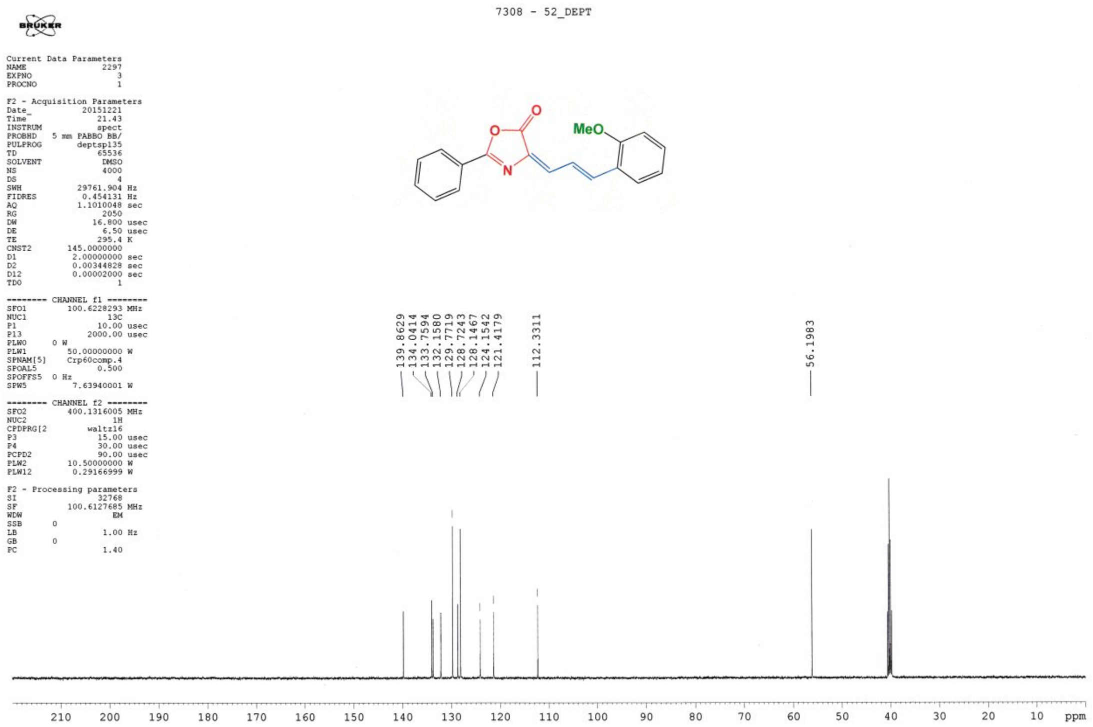

Enlarged spectrum in the range of 100-170 ppm.

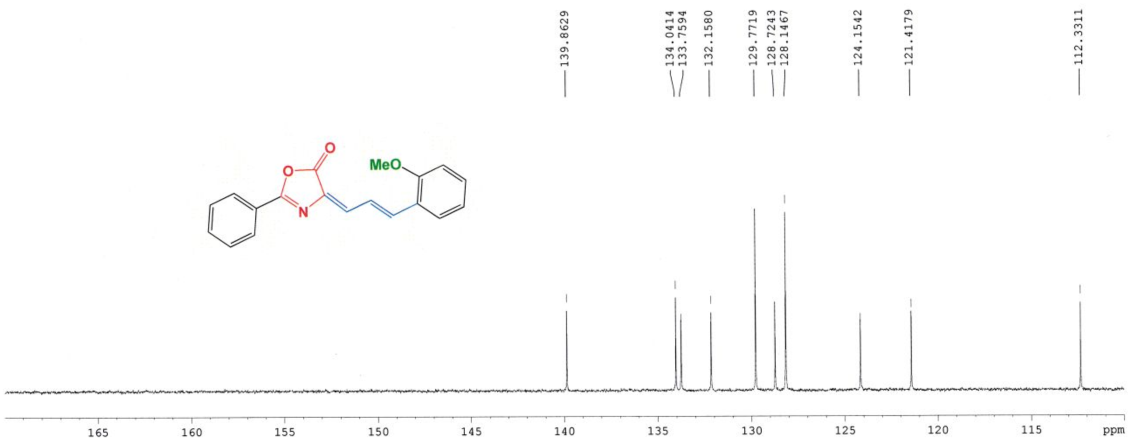

^1H–^1H COSY spectrum of Ox-π,π-Ph(OMe) in DMSO-d_6

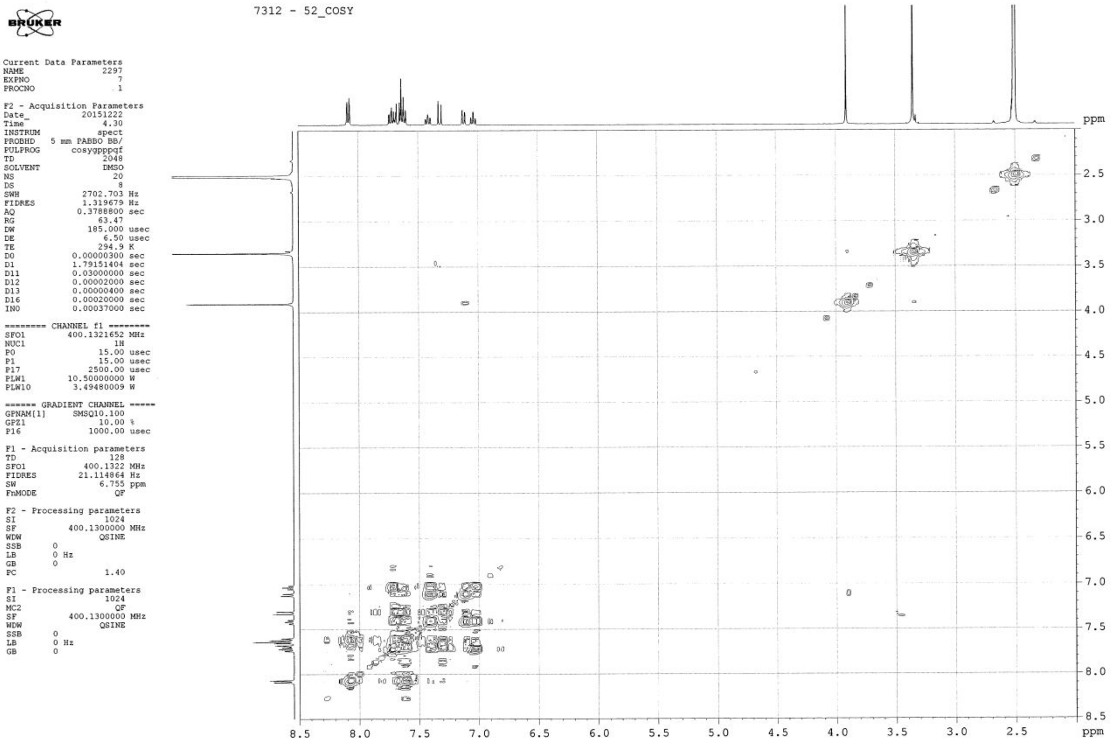

Enlarged spectrum

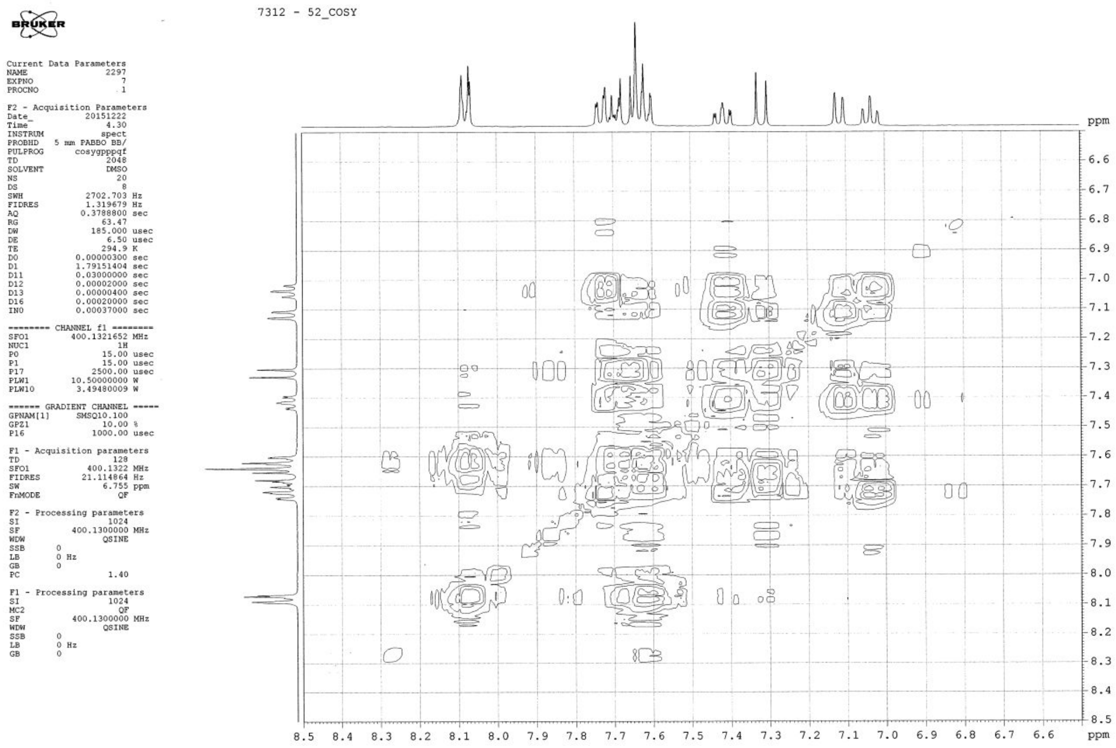

^{1}H–^{13}C HMBC spectrum of Ox-π,π-Ph(OMe) in DMSO-d_6

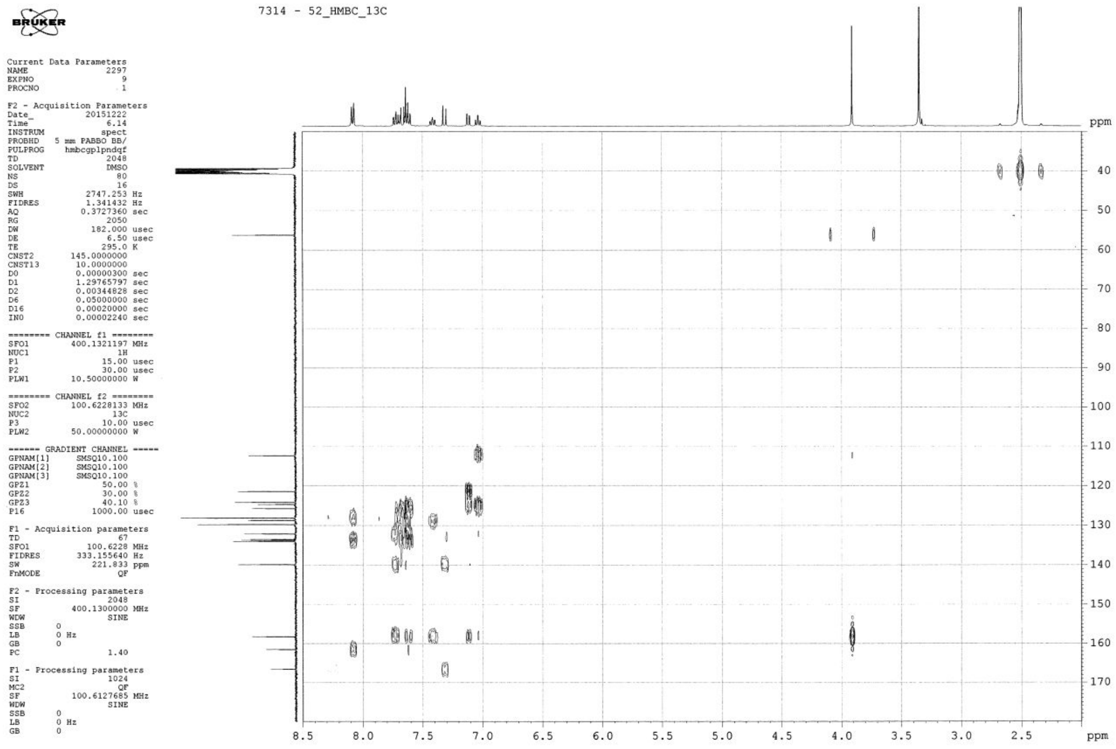

Enlarged spectrum

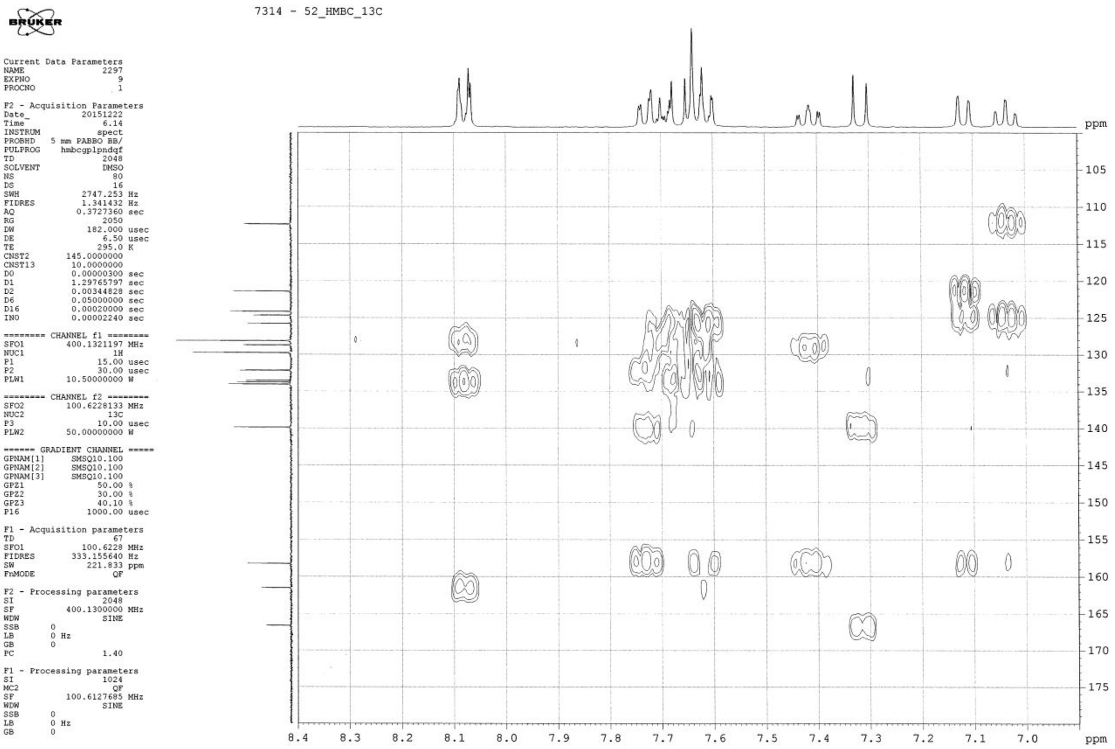

^{1}H–^{15}N HMBC spectrum of Ox-π,π-Ph(OMe) in DMSO-d_6

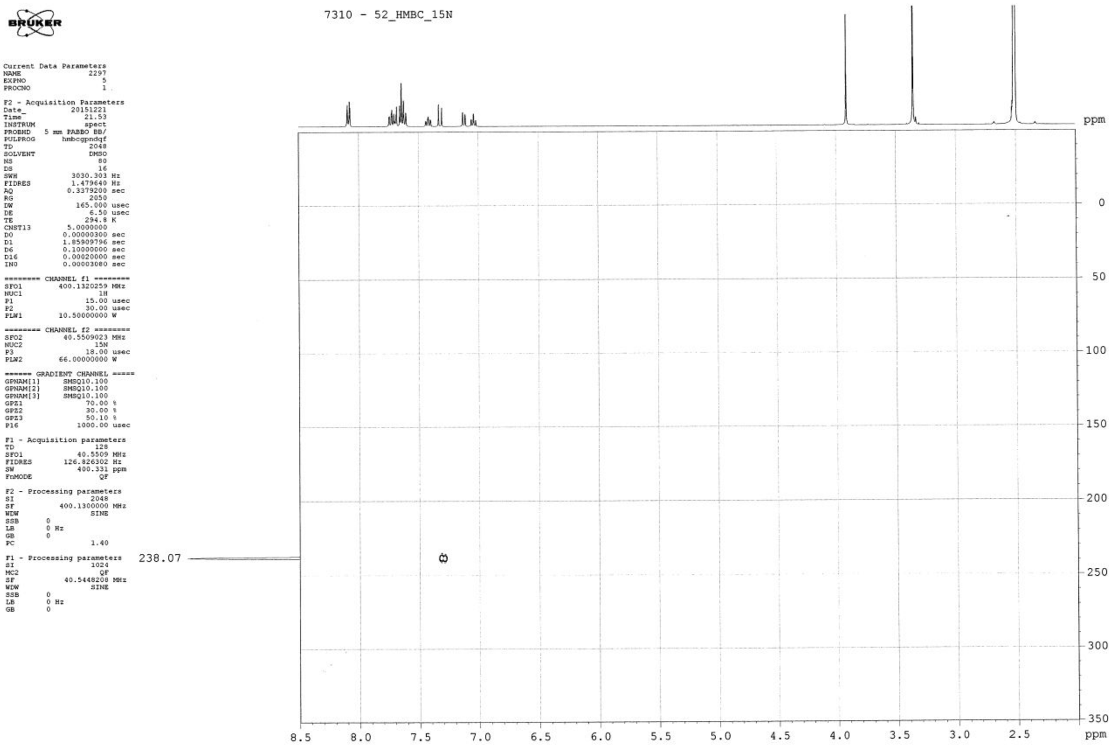

Enlarged spectrum

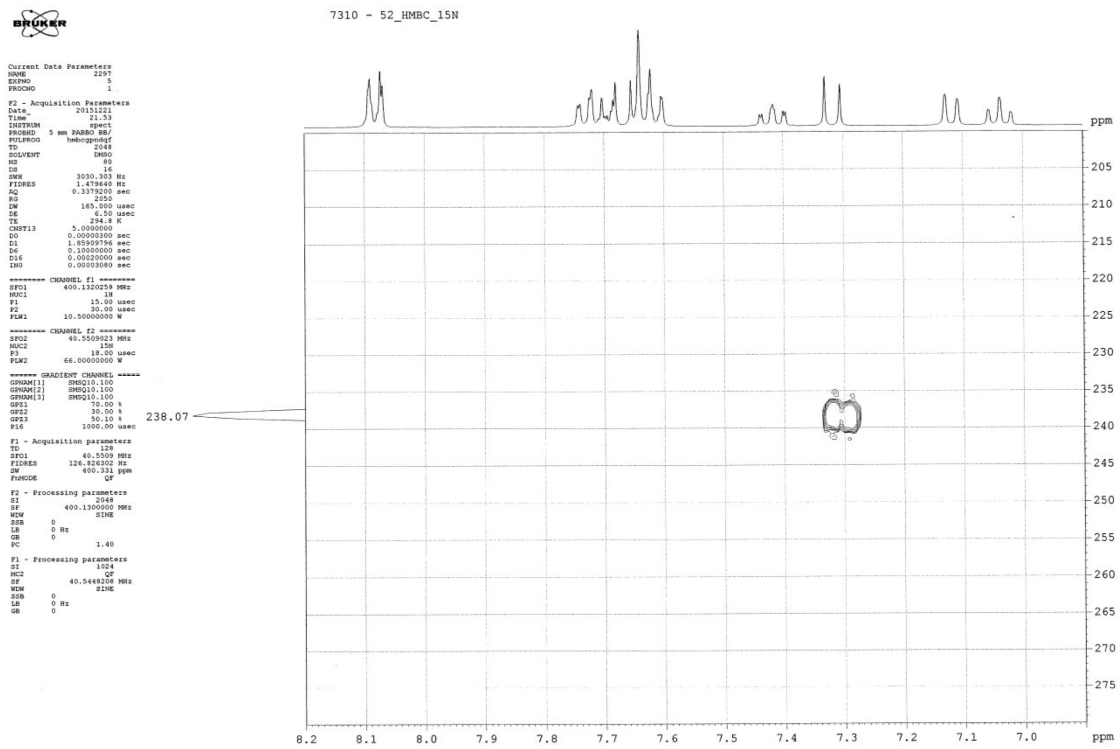

IR spectrum of Ox-π,π-Ph(OMe) (KBr pellet technique)

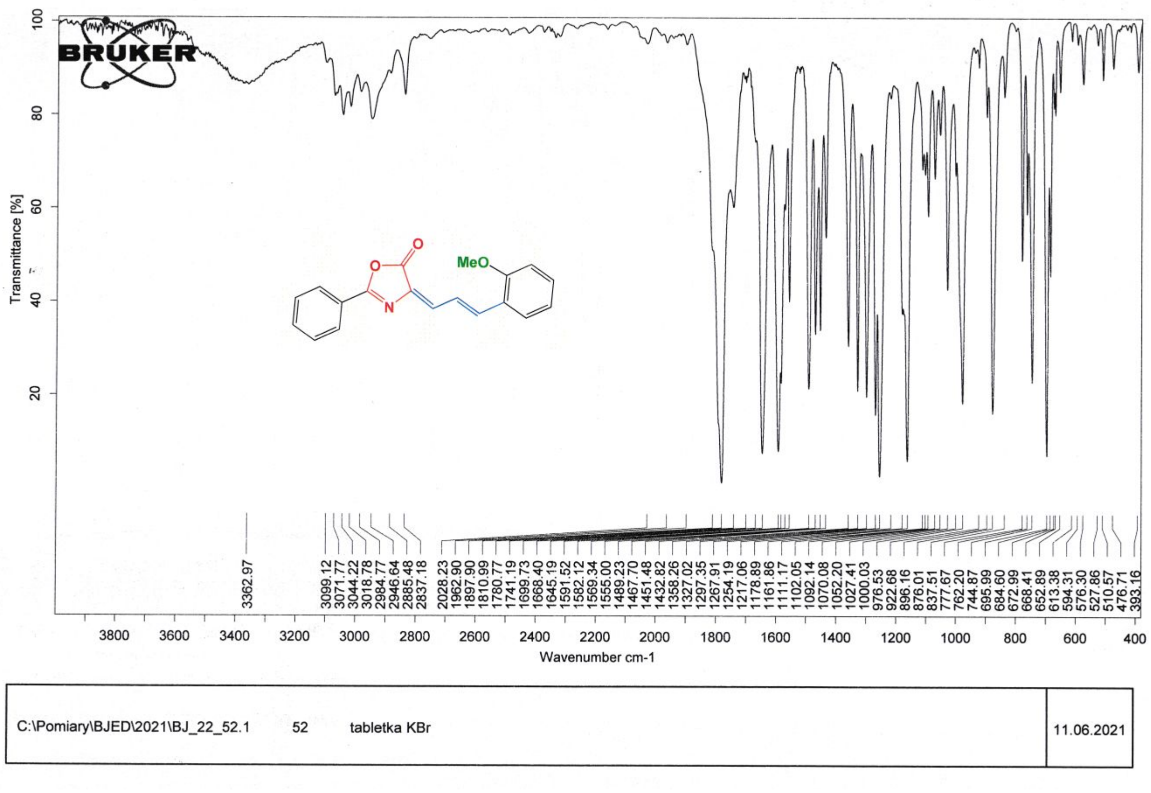

Enlarged spectrum in the range of 2000 - 400 cm^{-1}.

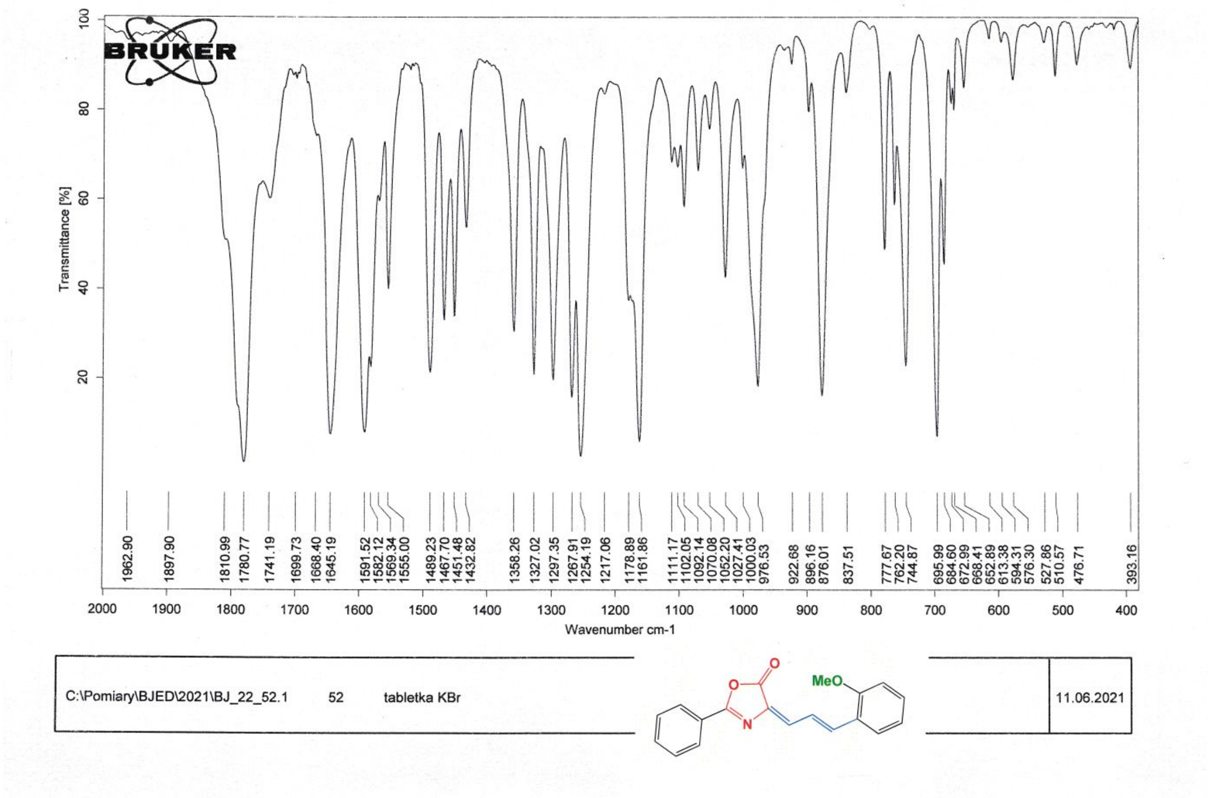

The NMR signals for different kinds of both protons and carbons at their respective positions are consistent with the structure of the synthesized compound. For example, the existence of a methine bridge between oxazolone ring and phenyl is evidenced by the observation of doublets at ca. 7.3 ppm and 7.68 ppm in *Z* conformation ($^3J_{H,H}$ =12 Hz) and triplet at 7.4 ppm. For the methoxy substituent that is in *ortho* position with respect to the methine units, the hydrogen signal is at 3.9 ppm and the carbon one is at 56.2 ppm. The IR spectra of the dye showed several strong bands in the range of 2000-1500 cm^{-1} which are mainly derived from various types of double bonds. The bands at ca. 1781 cm^{-1}, 1645 cm^{-1} and 1592 cm^{-1} due to the C=O, C=N and C=C double bonds, respectively, indicate the formation of oxazolone backbone. In the range below 1500 cm^{-1} called the "fingerprint region", the system of bands characteristic for the bands of stretching vibrations of single bonds, i.e. C-C, C-O, C-N, and bands corresponding to deformation vibrations, is visible.

HPLC analysis of Ox-π,π-Ph(OMe)

HPLC (Symmetry C18 column (3.5 µm, 4.6 mm × 75 mm), acetonitrile, flow rate = 1.0 mL/min, λ = 420 nm) t_R= 1.2 min).

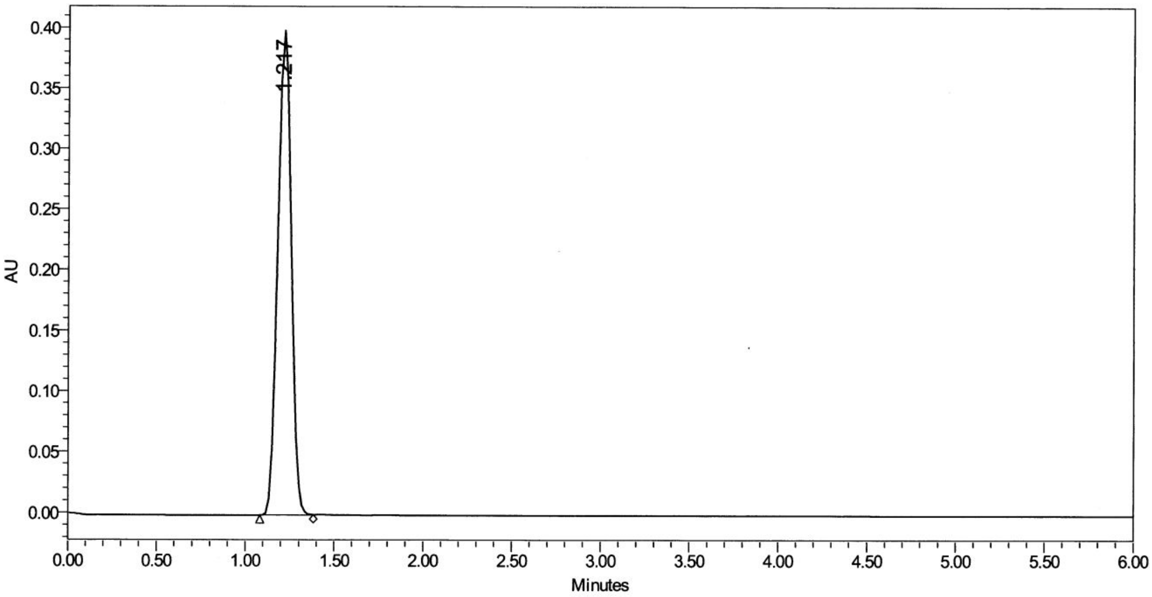

	RT (min)	Area (µV*sec)	% Area	Height (µV)	% Height
1	1.217	2071270	100.00	395726	100.00

HRMS (ESI-TOF) spectra of target compound

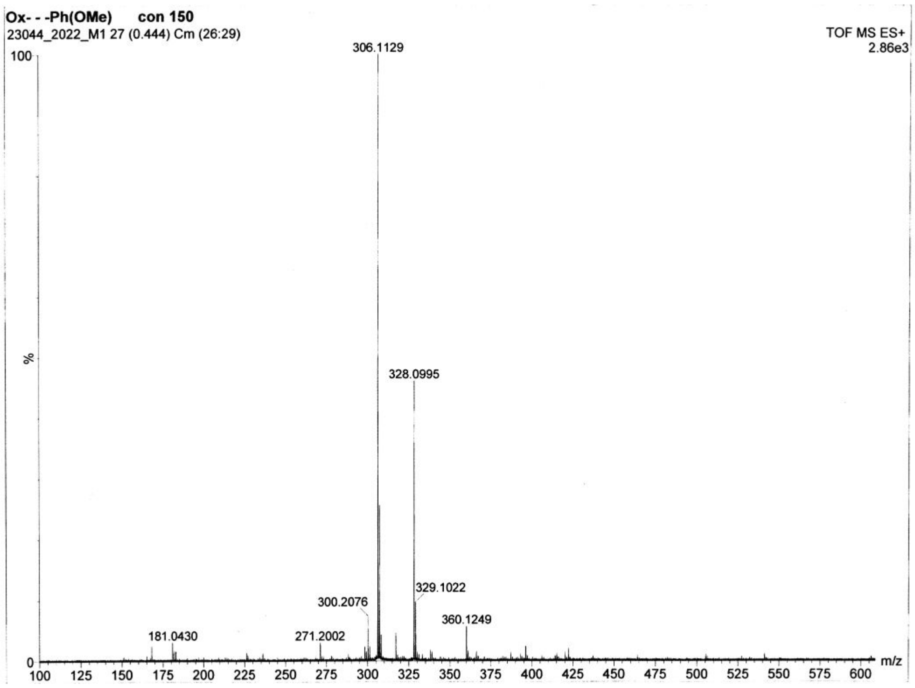

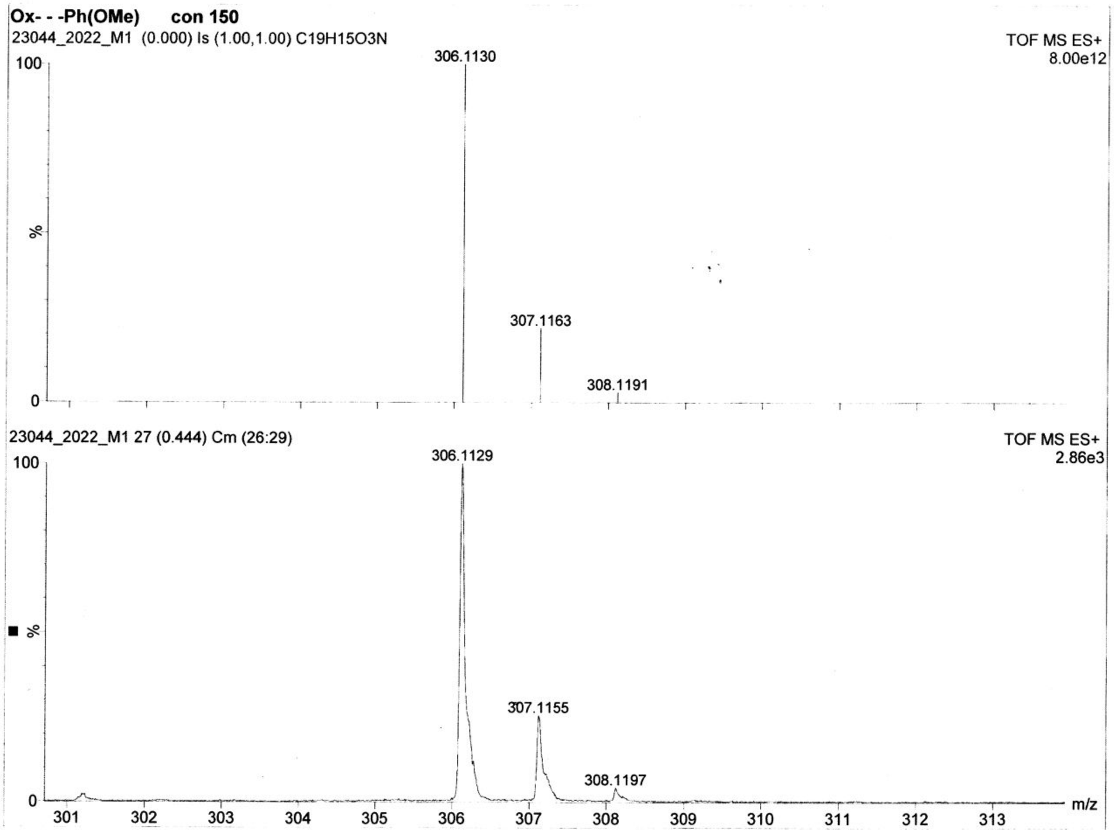

S1. Dipole moments determination

Based on quantum mechanical perturbation theory[1-4] and Inamdar's[5] studies on the absorption (ν_{Ab}) and fluorescence (ν_{Fl}) band shift of a spherical solute in solvents of varying permittivity (ε) and refractive index (n), the changes of the spectroscopic properties of the solute can be characterized by the difference in the absorption and fluorescence maxima positions.

$$\nu_{Ab} - \nu_{Fl} = m_1 \cdot f(\varepsilon, n) + const \quad \text{(S1)}$$

The model of dipole in a dielectric medium that is used to derive Eq. S1 also gives Eq. S2 for the sum of $\nu_{Ab} + \nu_{Fl}$.

$$\nu_{Ab} + \nu_{Fl} = m_2 \cdot [f(\varepsilon, n) + 2g(n)] + const \quad \text{(S2)}$$

ν_{Ab} and ν_{Fl} are the peak of absorption and emission (steady-state fluorescence) frequencies, where:

$$f(\varepsilon, n) = \frac{(2n^2+1)}{(n^2+2)} \cdot \left[\frac{(\varepsilon-1)}{(\varepsilon+2)} - \frac{(n^2-1)}{(n^2+2)}\right] \quad \text{(S3)}$$

is solvent polarity parameter and

$$g(n) = \frac{3}{2} \cdot \left[\frac{(n^4-1)}{(n^2+2)^2} \right] \quad \text{(S4)}$$

with

$$m_1 = \frac{2 \cdot (\mu_e - \mu_g)^2}{hca^3} \quad \text{(S5)}$$

and

$$m_2 = \frac{2 \cdot ({\mu_e}^2 - {\mu_g}^2)}{hca^3} \quad \text{(S6)}$$

where:

h is Planck's constant, *c* is the velocity of light in vacuum, μ_g and μ_e are the dipole moments in the ground and excited states, respectively. The parameters m_1 and m_2 can be obtained from the absorption and fluorescence band shifts (Eqs. S1 and S2). The Onsager radius a of the solute molecule can be determined by theoretical calculations. For $m_2 > m_1$ the excited state dipole moment may be determined according to Eq. S7:

$$\mu_e = \frac{m_2 + m_1}{m_2 - m_1} \cdot \mu_g \quad \text{(S7)}$$

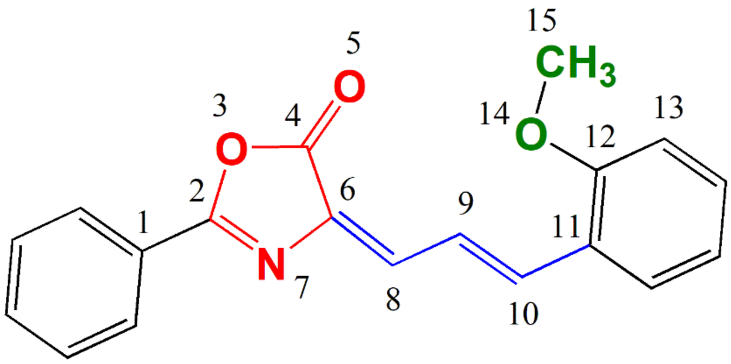

Figure S1. Atom numbering scheme.

Table S1. Solvent parameters and values of solvent polarity functions according to *Catalán*.

Solvent	Type[a)]	ε	n_{20}^{D}	E_T^N	SP	SdP	SA	SB
n-Hexane, Hex	NHB	1.8863	1.37486	0.009	0.616	0	0	0.056
2,2,4-TriMethylPentane, TMP	NHB	1.94	1.39145	n.d.	0.618	0	0	0.044
MethylCycloHexane, MCH	NHB	2.02	1.42312	n.d.	0.675	0	0	0.069
DiButyl Ether, Bu_2O	HBA	3.10	1.3992	0.071	0.672	0.175	0	0.637
DiEthyl Ether, Et_2O	HBA	4.335	1.35243	0.117	0.617	0.385	0	0.562
EthylAcetate, EtOAc	HBA	6.053	1.37239	0.099	0.656	0.603	0	0.542
TetraHydroFuran, THF	HBA	7.58	1.40716	0.207	0.714	0.634	-0.012	0.591
Aceton, MeAc	HBA	20.70	1.35868	0.355	0.651	0.907	0.007	0.475
Acetonitryl, MeCN	HBA-HBD	35.94	1.34411	0.46	0.645	0.974	0.044	0.286
N,N-DiMethylFormamide, DMF	HBA	36.71	1.43047	0.404	0.759	0.977	0.031	0.613
DiMethylSulfoxide, DMSO	HBA	46.45	1.4793	0.444	0.83	1	0.072	0.647
1-Butanol, 1-BuOH	HBA-D	17.51	1.3993	0.602	0.674	0.655	0.341	0.809
2-Propanol, 2-PrOH	HBA-D	19.92	1.3772	0.546	0.633	0.808	0.283	0.83
1-Propanol, 1-PrOH	HBA-D	20.45	1.38556	0.617	0.658	0.748	0.367	0.782
Ethanol, EtOH	HBA-D	24.55	1.36143	0.654	0.633	0.7830	0.400	0.6580
Methanol, MeOH	HBA-D	32.66	1.3284	0.762	0.608	0.904	0.605	0.545

[a)] NHB = non-hydrogen-bonding solvent; HBA = hydrogen bond acceptor; HBD = hydrogen bond donor; HBA-D = amphiprotic hydrogen bond acceptor-donor.

Table S2. Selected structural parameters of the *E* isomer in the ground state.

	GP	TMP	MCH	Bu_2O	Et_2O	THF	MeAc	MeCN	DMF	DMSO	Water
bond lengths											
C1-C2	1.45268	1.45261	1.45260	1.45255	1.45251	1.45246	1.45240	1.45238	1.45238	1.45238	1.45236
C4-O5	1.19802	1.20000	1.20012	1.20112	1.20180	1.20267	1.20355	1.20379	1.20381	1.20381	1.20398
C6-C8	1.36614	1.36699	1.36704	1.36747	1.36776	1.36813	1.36851	1.36861	1.36861	1.36861	1.36868
C8-C9	1.42848	1.42779	1.42775	1.42740	1.42718	1.42691	1.42665	1.42658	1.42658	1.42658	1.42653
C9-C10	1.35857	1.35939	1.35945	1.35985	1.36011	1.36044	1.36076	1.36084	1.36085	1.36085	1.36091
C10-C11	1.44970	1.44894	1.44889	1.44848	1.44825	1.44793	1.44762	1.44754	1.44753	1.44753	1.44747
C12-O14	1.34612	1.34566	1.34566	1.34567	1.34579	1.34601	1.34634	1.34645	1.34646	1.34646	1.34653
O14-C15	1.41029	1.41330	1.41348	1.41493	1.41589	1.41711	1.41828	1.41860	1.41862	1.41862	1.41884
bond angles											
O3-C4-O5	121.19231	121.04550	121.03736	120.96265	120.91404	120.85238	120.79363	120.77915	120.77833	120.77833	120.76745
O5-C4-C6	134.43889	134.47516	134.47531	134.49412	134.50462	134.51459	134.52125	134.52103	134.52101	134.52101	134.52054
N7-C6-C8	121.08601	121.07359	121.07232	121.06541	121.06124	121.05344	121.04828	121.04611	121.04598	121.04598	121.04488
C6-C8-C9	133.74978	133.84333	133.84838	133.88683	133.90972	133.93872	133.96446	133.97177	133.97218	133.97218	133.97768
C8-C9-C10	128.29306	128.27749	128.27491	128.25431	128.24155	128.21793	128.19219	128.18545	128.18504	128.18504	128.17956
C9-C10-C11	125.48688	125.52565	125.52835	125.71804	125.55952	125.57974	125.60428	125.60992	125.61025	125.61025	125.61465
C10-C11-C12	118.63656	118.68021	118.68381	118.71804	118.74463	118.78171	118.82340	118.83664	118.83739	118.83739	118.84625
C11-C12-O14	116.03738	116.14215	116.14864	116.20167	116.23538	116.27939	116.32191	116.33437	116.33505	116.33505	116.34262
C13-C12-O14	123.46002	123.41632	123.41286	123.38394	123.36217	123.33107	123.29660	123.28604	123.28545	123.28545	123.27845
C12-O14-C15	119.13797	119.04883	119.04317	118.98932	118.95169	118.90492	118.85834	118.84706	118.84640	118.84640	118.83796
dihedral angles											
O5-C4-C6-C8	-0.00963	-0.08253	-0.08883	-0.16520	-0.18548	-0.21147	-0.20309	-0.20265	-0.20197	-0.20197	-0.18961
C6-C8-C9-C10	0.00165	-0.04316	-0.04890	-0.05255	-0.06283	-0.04610	-0.00097	0.03034	0.03190	0.03190	0.05781
C8-C9-C10-C11	-179.99849	-179.76791	-179.73571	-179.64013	-179.60267	-179.58381	-179.60118	-179.61622	-179.61720	-179.61720	-179.63167
C9-C10-C11-C12	179.99320	-178.76766	-178.64343	-178.24689	-178.13838	-178.07023	-178.01028	-178.00328	-178.00356	-178.00356	-178.00067
C11-C12-O14-C15	179.96930	-179.89011	-179.90173	-179.84127	-179.87850	-179.89753	-179.91961	-179.91718	-179.91729	-179.91729	-179.92084
C13-C12-O14-C15	-0.03464	0.15044	0.14011	0.22093	0.18874	0.17473	0.16018	0.16496	0.16493	0.16493	0.16290

Table S3. Selected structural parameters of the *Z* isomer in the ground state.

	GP	TMP	MCH	Bu_2O	Et_2O	THF	MeAc	MeCN	DMF	DMSO	Water
bond lengths											
C1-C2	1.45338	1.45311	1.45310	1.45310	1.45296	1.45285	1.45288	1.45277	1.45276	1.45275	1.45274
C4-O5	1.19281	1.19413	1.19422	1.19498	1.19546	1.19614	1.19689	1.19706	1.19708	1.19709	1.19723
C6-C8	1.35446	1.35480	1.35481	1.35478	1.35498	1.35504	1.35510	1.35523	1.35522	1.35522	1.35525
C8-C9	1.44418	1.44417	1.44416	1.44426	1.44408	1.44397	1.44413	1.44381	1.44382	1.44381	1.44378
C9-C10	1.34901	1.34990	1.34942	1.34920	1.34953	1.34961	1.34947	1.34977	1.34979	1.34980	1.34982
C10-C11	1.45864	1.45858	1.45861	1.45907	1.45876	1.45888	1.45916	1.45882	1.45884	1.45883	1.45882
C12-O14	1.36664	1.36762	1.36767	1.36814	1.36842	1.36880	1.36911	1.36917	1.36920	1.36920	1.36926
O14-C15	1.42420	1.42538	1.42544	1.42564	1.42615	1.42643	1.42634	1.42669	1.42670	1.42668	1.42673
bond angles											
O3-C4-O5	121.66617	121.58345	121.57453	121.49244	121.47991	121.42983	121.36090	121.37666	121.37466	121.37469	121.36358
O5-C4-C6	134.23781	134.23101	134.23169	134.27257	134.24446	134.25428	134.27955	134.24596	134.24557	134.24495	134.24856
N7-C6-C8	123.02403	123.04218	123.04083	123.09072	123.06208	123.07119	123.09645	123.07026	123.07123	123.06995	123.06748
C6-C8-C9	129.88125	130.07933	130.08412	129.91697	130.13484	130.17015	130.07548	130.25594	130.25556	130.25805	130.27814
C8-C9-C10	130.56305	130.80436	130.81222	130.68212	130.91555	130.98087	130.89397	131.12395	131.12438	131.12882	131.15550
C9-C10-C11	129.16219	129.51434	129.52545	129.25415	129.59867	129.63485	129.37715	129.70686	129.70663	129.70763	129.72668
C10-C11-C12	119.04175	119.03674	119.03575	119.11945	119.05107	119.05691	119.13900	119.05057	119.05444	119.05585	119.04941
C11-C12-O14	119.42794	119.94218	119.94471	119.45573	119.93989	119.92346	119.45873	119.91349	119.91814	119.91831	119.92137
C13-C12-O14	119.64770	119.08562	119.08012	119.55201	119.04610	119.04229	119.49448	119.02929	119.02377	119.02288	119.01690
C12-O14-C15	114.41448	114.44228	114.44180	114.37732	114.42166	114.42136	114.40005	114.42543	114.42985	114.43185	114.43457
dihedral angles											
O5-C4-C6-C8	-1.81399	-1.66007	-1.67327	-1.75442	-1.72238	-1.78936	-1.79359	-1.92686	-1.92261	3.64780	-1.95185
C6-C8-C9-C10	44.44135	44.69813	44.69601	45.18625	44.92317	44.97717	45.28753	44.94926	44.95355	44.93657	44.94329
C8-C9-C10-C11	8.53866	8.18705	8.19188	8.23313	8.07640	8.02799	7.95857	8.00607	7.99901	8.01130	7.98685
C9-C10-C11-C12	-151.53765	-153.13006	-153.17124	-152.31742	-153.69067	-153.78495	-152.97705	-154.22768	-154.20849	-154.21692	-154.29210
C11-C12-O14-C15	95.70253	85.69471	85.63072	85.23183	85.67951	85.92389	85.30729	86.12607	86.16452	86.17402	86.15154
C13-C12-O14-C15	-86.47736	-96.65317	-96.71226	-96.91068	-96.65188	-96.36487	-96.81912	-96.15631	-96.11669	-96.10468	-96.12365

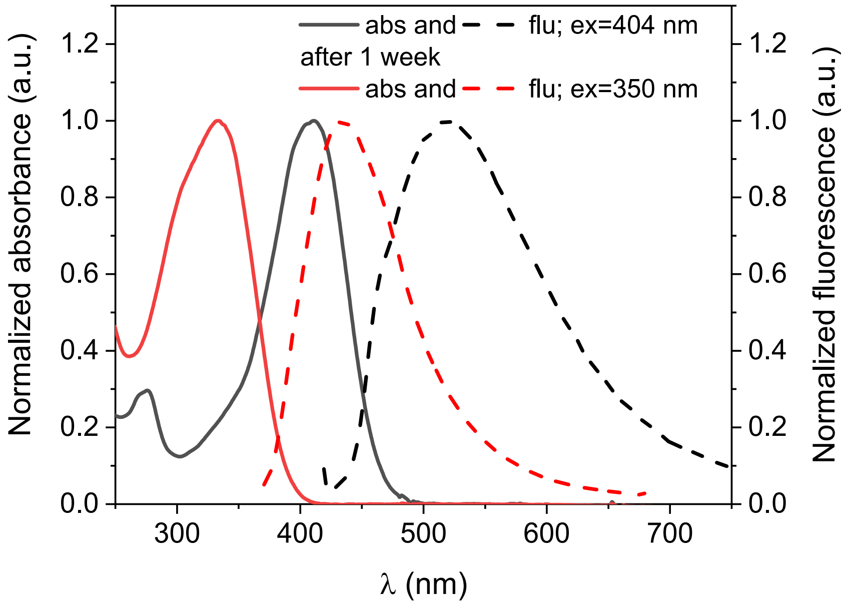

Figure S2. Changes in the position of the absorption and fluorescence bands of Ox-π,π-Ph(OMe) in EtOH after one week of storing the diluted solution at room temperature.

(a) **(b)**

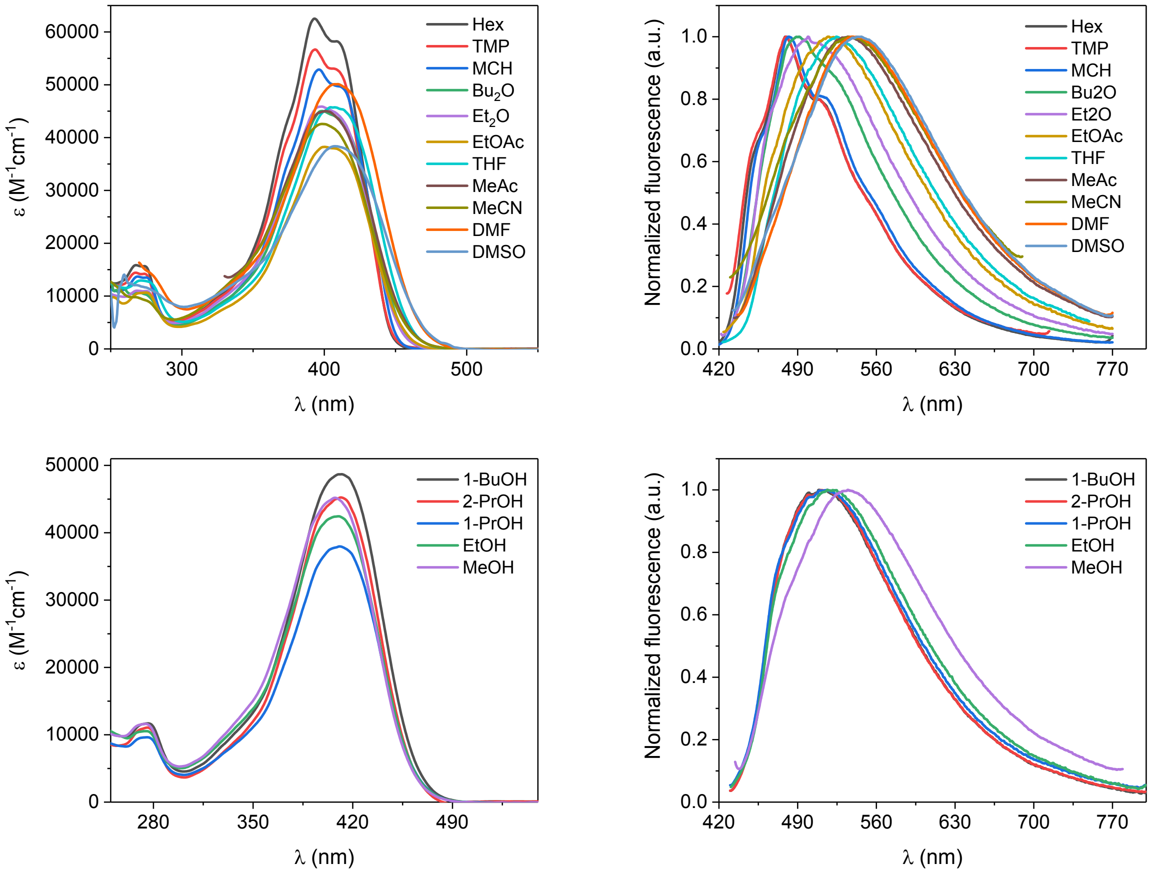

Fig. S3. The normalized electronic absorption (a) and fluorescence (b) spectra of Ox-π,π-Ph(OMe) in solvents of different polarities.

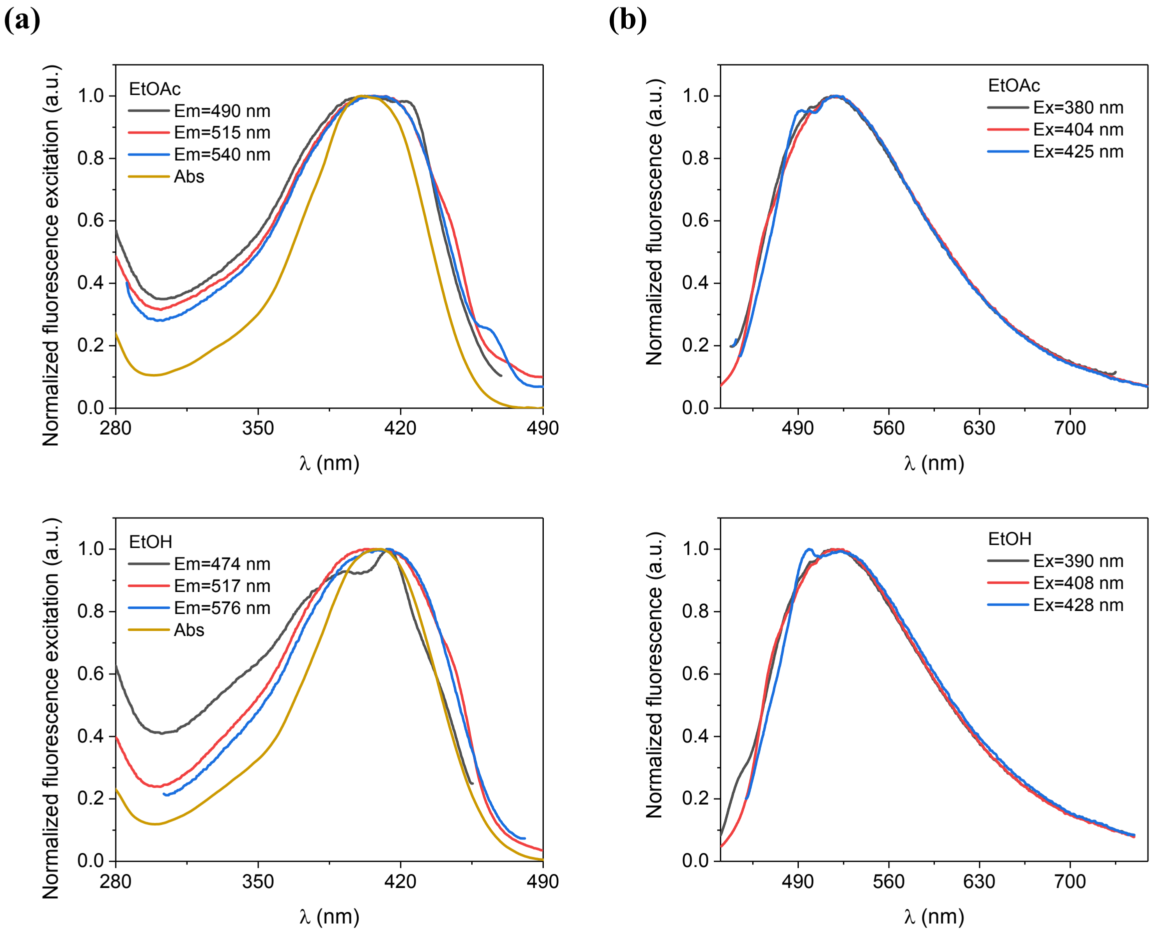

Figure S4. Normalized fluorescence excitation (a) and fluorescence (b) spectra of Ox-π,π-Ph(OMe) in EtOAc and EtOH recorded for different observation and emission wavelengths.

S2. Spectral properties

The obtained excitation energies (λ_{Ab}) are given in Table 3. The effect of the solvent on λ_{Ab} is shown in Table S4. As previously reported,[6-7] calculations of linear optical properties were performed using only PBE0. Considering the vertical values, the values closest to the experimental are obtained for trans isomers. The average error for these conformers is 4.43 nm. For *Z* isomer, the maximum λ_{Ab} shifts towards longer waves and the relative error of the measured values increases to 35.06 nm. Employing the state-specific corrected linear response (λ_{Ab}^{cLR}) approach (Table S5) increases the size of this displacement. Taking into account λ_{Ab}^{vert} and λ_{Ab}^{cLR} it should be assumed that the occurrence of the tested dye in the form of *Z* isomer will cause a bathochromic shift relative to *E* by an average of 30 nm. Moreover, the position of absorption maximum band of *E* and *Z* isomers is sensitive to changes in the environmental polarity. Increasing the solvent polarity results in a bathochromic shift of the λ_{Ab}^{vert} and λ_{Ab}^{cLR}. However, the non-monotonous behavior of λ_{Ab} is observed. The most intense

maximum above 450 nm is almost a pure HOMO→LUMO transition for each isomer. However, contributions from other orbitals are not negligible. Therefore, an additional maximum shifted towards shorter wavelengths may appear. In the case of *Z* isomers they are associated with HOMO-1→LUMO and HOMO-2→LUMO transitions, while for *E* with HOMO-1→LUMO and HOMO-4→LUMO.

The use of PBE0 also leads to reliable maximum fluorescence (λ_{Fl}) results (Table S6). Similarly as in the case of λ_{Ab}, the closest measured values are obtained for trans isomers, with an average error of 6.55 nm. Isomerization causes significant batochromic shift, and the error increases to 35.58 nm. In this case, the maximum λ_{Fl} shift cannot be clearly determined during *E*-*Z* isomerization, as this relationship is closely dependent on the solvent. For example, $\Delta\lambda_{FL}^{E-Z}$ in Bu_2O is 58.44 nm and in DMSO is 16.38 nm. In addition, in contrast to experimental values, theoretical ones exhibit non-monotonous behavior in the function of solvent polarity. In conjunction with absorption considerations, therefore, specific interactions in the solvent-solute system are to be expected, as well as the occurrence of *H*-bonds.

Table S4. The vertical excitation energies (in nm).

	Z		*E*	
	λ_{Ab}	*f*	λ_{Ab}	*f*
GP	428.17	0.4401	402.58	1.1089
TMP	432.31	0.5232	405.43	1.2211
MCH	432.57	0.5278	405.56	1.2268
Bu_2O	434.16	0.5174	404.86	1.2142
Et_2O	435.08	0.5210	405.26	1.1987
THF	436.56	0.5304	405.42	1.2045
MeAc	437.70	0.5170	404.79	1.1882
MeCN	437.98	0.5267	405.10	1.1832
DMF	438.31	0.5387	405.49	1.2027
DMSO	438.33	0.5370	405.43	1.1994
Water	438.18	0.5261	405.00	1.1794

Table S5. The cLR corrected excitation energies (in nm).

	λ_{Ab}^{cLR}	
	Z	*E*
TMP	453.34	413.76
MCH	454.80	414.37
Bu_2O	455.32	412.88
Et_2O	454.28	412.71
THF	457.71	413.61
MeAc	456.84	411.96
MeCN	456.48	412.30
DMF	460.19	414.02
DMSO	459.69	413.76
Water	456.19	412.03

Table S6. The vertical de-excitation energies (in nm).

	λ_{Fl}	
	Z	*E*
GP	474.16	480.71
TMP	515.60	498.86
MCH	514.35	500.81
Bu_2O	588.75	499.21
Et_2O	573.17	496.88
THF	565.91	533.11
MeAc	549.95	531.86
MeCN	546.11	541.27
DMF	552.70	547.99
DMSO	551.68	547.00
Water	550.31	541.03

Table S7. Fluorescence lifetimes (τ; ns), their amplitudes (α; %), average fluorescence lifetimes (τ_{av}; ns) and correlation coefficient (χ^2) for Ox-π,π-Ph(OMe) in different solvents.

**Solvent**	τ_1	α_1	τ_2	α_2	τ_3	α_3	χ^2	τ_{av}
Hex	0.146	97.93	0.859	2.07			1.816	0.16
TMP	0.158	96.92	1.17	1.08			1.661	0.17
MCH	0.135	98.05	1.23	1.95			1.412	0.16
Bu_2O	0.210	17.45	1.137	82.56			1.569	0.98
Et_2O	0.108	98.2	1.116	1.8			1.78	0.13
EtOAc	0.109	97.67	1.562	2.33			1.545	0.14
THF	0.083	94.87	2.606	5.13			1.44	0.21
MeAc	0.082	88.35	2.457	11.65			1.477	0.36
MeCN	0.061	84.6	2.708	15.4			1.824	0.47
DMF	0.105	67.74	1.371	19.32	4.533	12.94	1.926	0.92
DMSO	0.101	39.11	1.58	24.6	6.565	36.29	1.748	2.81
1-BuOH	0.112	97.37	1.869	2.63			1.762	0.16
2-PrOH	0.119	96.63	1.971	3.37			1.716	0.18
1-PrOH	0.149	97.79	1.818	2.21			1.943	0.19
EtOH	0.098	98.59	2.596	1.41			1.916	0.13
MeOH	0.08	98.86	1.718	1.14			1.991	0.10

Table S8. Radiative (k_r; 10^6 s^{-1}) and non-radiative (k_{nr}; 10^9 s^{-1}) rate constants of Ox-π,π-Ph(OMe) dye.

Solvent	k_r	k_{nr}	k_{nr}/k_r
Hex	12.69	6.21	489
TMP	13.21	5.90	447
MCH	15.02	6.38	425
Bu_2O	2.24	1.02	457
Et_2O	4.22	7.92	1880
EtOAc	4.30	7.00	1627
THF	2.68	4.71	1753
MeAc	1.09	2.79	2550
MeCN	0.55	2.13	3845
DMF	0.41	1.08	2631
DMSO	0.16	0.36	2173
1-BuOH	3.77	6.31	1674
2-PrOH	3.28	5.51	1680
1-PrOH	2.83	5.38	1901
EtOH	2.64	7.50	2840
MeOH	2.13	10.13	4766

S3. Physico-chemical properties

	Z	*E*
GP	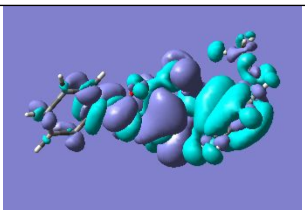	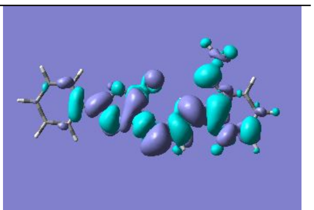
TMP	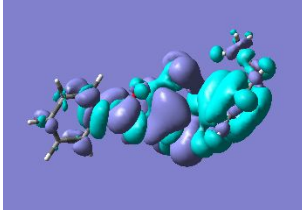	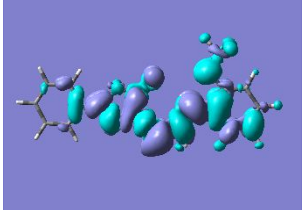
MCH	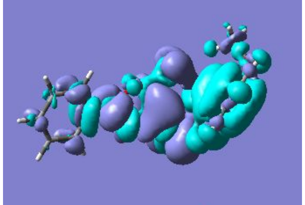	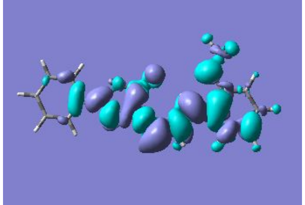
Bu_2O	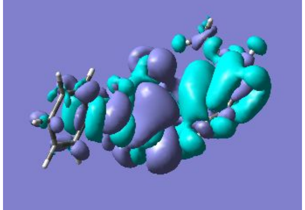	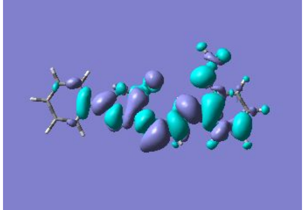
Et_2O	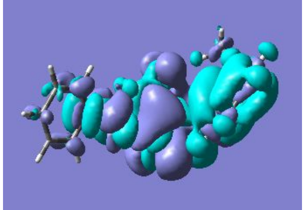	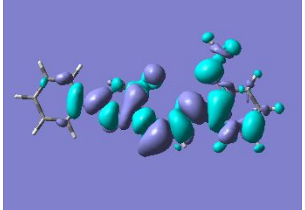
THF	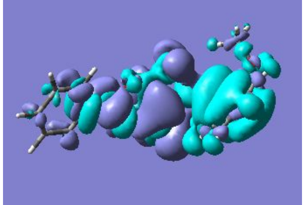	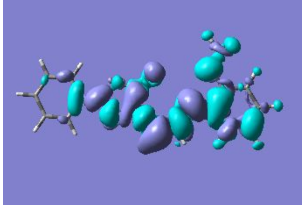
MeAc	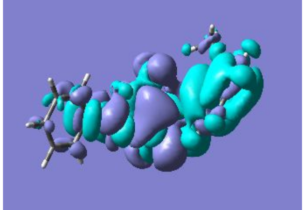	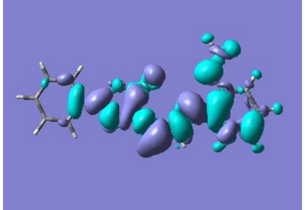
MeCN	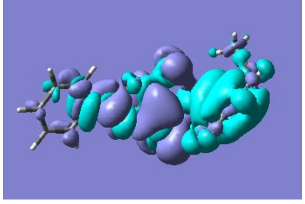	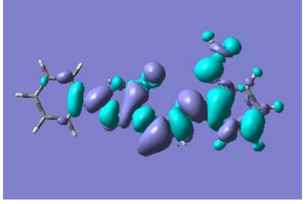

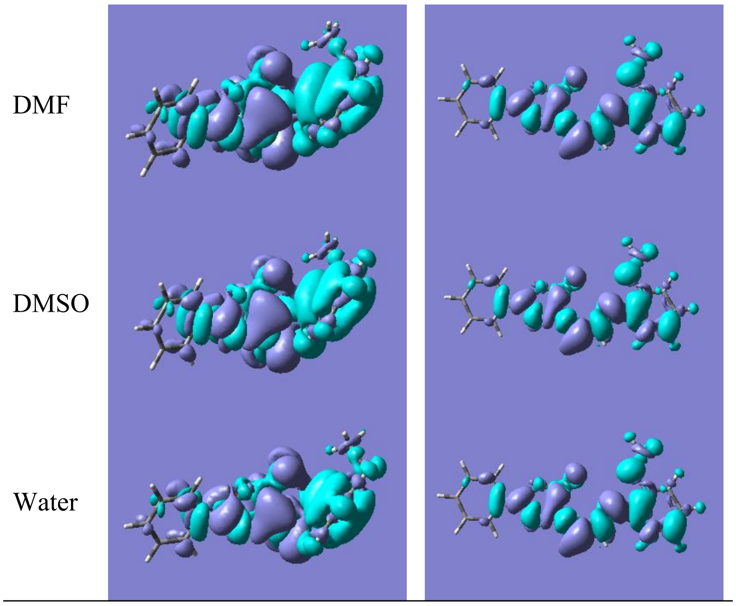

Figure S5. Density difference plots.

Although E_{GAP} is slightly decreased in the function of solvent polarity (Table S9), the investigated conformers are characterized by a low value of chemical hardness (η) and should be treated as soft molecules with very high reactivity. Moreover, the calculated electronegativity (χ), which is greater than 4.0 eV for both molecules, indicates an easy formation of covalent bonds during various chemical processes.

To predict reactive sites for nucleophilic (positive, blue regions) and electrophilic (negative, red and yellow regions) attack of the investigated conformers, the Molecular Electrostatic Potential (MEP) surfaces were calculated (Fig. 5 (g, h)). The most negative region is located on the oxygen atom O5 connected by a double bond to oxazolidine (V(r) > -0.04 a.u.). The maximum positive site is localized on the π-electron bridge. In this fragment of compounds the positive charge is higher than for the methyl group. The *E*-*Z* isomerization does not change the specific zones for nucleophilic and electrophilic attacks and intermolecular interactions.

Table S9. The frontier orbital energies in different solvents. All values are given in eV.

Z isomer	E_{HOMO}	E_{LUMO}	E_{GAP}	η	μ	χ
GP	-6.2524	-2.6582	3.5941	1.7971	-4.4553	4.4553
TMP	-6.3131	-2.7249	3.5881	1.7941	-4.5190	4.5190
MCH	-6.3163	-2.7282	3.5881	1.7941	-4.5223	4.5223
Bu_2O	-6.3359	-2.7369	3.5990	1.7995	-4.5364	4.5364
Et_2O	-6.3631	-2.7734	3.5898	1.7949	-4.5683	4.5683
THF	-6.3874	-2.7970	3.5903	1.7952	-4.5922	4.5922
MeAc	-6.4072	-2.8087	3.5985	1.7992	-4.6080	4.6080
MeCN	-6.4178	-2.8286	3.5892	1.7946	-4.6232	4.6232
DMF	-6.4181	-2.8289	3.5892	1.7946	-4.6235	4.6235
DMSO	-6.4181	-2.8289	3.5892	1.7946	-4.6235	4.6235
Water	-6.4227	-2.8338	3.5890	1.7945	-4.6283	4.6283
E isomer	E_{HOMO}	E_{LUMO}	E_{GAP}	η	μ	χ
GP	-5.8591	-2.7116	3.1476	1.5738	-4.2854	4.2854
TMP	-5.9261	-2.7943	3.1318	1.5659	-4.3602	4.3602
MCH	-5.9307	-2.7998	3.1310	1.5655	-4.3652	4.3652
Bu_2O	-5.9699	-2.8466	3.1233	1.5617	-4.4082	4.4082
Et_2O	-5.9971	-2.8787	3.1184	1.5592	-4.4379	4.4379
THF	-6.0328	-2.9206	3.1122	1.5561	-4.4767	4.4767
MeAc	-6.0692	-2.9628	3.1065	1.5532	-4.5160	4.5160
MeCN	-6.0790	-2.9742	3.1048	1.5524	-4.5266	4.5266
DMF	-6.0796	-2.9750	3.1046	1.5523	-4.5273	4.5273
DMSO	-6.0796	-2.9750	3.1046	1.5523	-4.5273	4.5273
Water	-6.0864	-2.9829	3.1035	1.5517	-4.5346	4.5346

Table S10. CT parameters for the bright low-lying excited state of Ox-π,π-Ph(OMe).

	Z		*E*	
	q_{CT}	D_{CT}	q_{CT}	D_{CT}
GP	0.510	1.511	0.372	1.149
TMP	0.506	1.528	0.389	1.280
MCH	0.506	1.530	0.389	1.293
Bu_2O	0.512	1.602	0.394	1.408
Et_2O	0.506	1.602	0.396	1.476
THF	0.504	1.614	0.401	1.539
MeAc	0.507	1.663	0.405	1.612
MeCN	0.501	1.636	0.406	1.640
DMF	0.501	1.624	0.407	1.623
DMSO	0.501	1.626	0.407	1.633
Water	0.501	1.641	0.407	1.656

Table S11. Calculated values of dipole moments (in D) for the ground and CT excited state.

	Z		*E*	
	μ_{GS}	μ_{CT}	μ_{GS}	μ_{CT}
GP	2.39	7.93	1.51	8.90
TMP	2.78	8.47	1.76	10.58
MCH	2.80	8.50	1.81	10.67
Bu_2O	2.86	8.60	1.91	11.69
Et_2O	3.05	9.08	2.00	11.78
THF	3.17	9.26	2.12	13.05
MeAc	3.16	9.50	2.24	12.96
MeCN	3.30	9.52	2.27	13.05
DMF	3.31	9.45	2.27	13.11
DMSO	3.31	9.47	2.28	13.16
Water	3.32	9.47	2.30	13.16

Table S12. Nonlinear properties of Ox-π,π-Ph(OMe) isomers. Values are given in (a.u.).

	Z		*E*	
	α	*β*	*α*	*β*
GP	269.89	666.33	326.75	2388.55
TMP	304.96	787.45	372.45	3900.45
MCH	307.10	793.30	375.21	4003.59
Bu_2O	323.97	778.23	398.19	4900.97
Et_2O	337.19	853.93	413.51	5534.37
THF	353.13	881.99	433.16	6375.93
MeAc	368.61	830.47	452.97	7241.75
MeCN	374.40	930.82	458.33	7476.36
DMF	374.65	930.07	458.63	7489.68
DMSO	375.89	931.72	460.06	7554.55
Water	377.00	939.70	462.42	7655.47

S4. Cartesian coordinates of optimized structures

Isomer *Z (cis)*

1. Ground state

Bu_2O

C	-0.3239323932	1.3794746997	-0.3777618198
C	-4.0138377365	-0.9749277274	0.9629896078
C	-3.5749849104	-0.025145483	0.0356620246
C	-4.469140047	0.494545999	-0.907260289
C	-5.7860279283	0.0650478452	-0.9196187904
C	-6.2218943448	-0.8825172676	0.0049231482
C	-5.3353236649	-1.399967661	0.9434568851
H	-3.3191312676	-1.3745885068	1.692561038
H	-4.1151203572	1.2309435697	-1.6198838063
H	-6.4778743294	0.4686627414	-1.6512081616
H	-7.2542067069	-1.2166069517	-0.0075235866
H	-5.673890165	-2.137169435	1.6635795663
C	-0.116534353	0.4772848957	0.7720724999
C	1.9511625955	2.4621597606	-0.6678499131
H	2.2285824311	3.5157959429	-0.6630579481
C	2.9581582973	1.5855138841	-0.4734436827

H	3.9204853242	2.0099275	-0.197574779
C	0.5517430586	2.2413702333	-0.9485157789
H	0.1004719981	2.922922346	-1.6691027093
C	2.9675313723	0.1368467149	-0.6471269137
C	2.2014211213	-0.5160688034	-1.6222277604
C	3.851484963	-0.6382230503	0.1220303882
C	2.2828819467	-1.8873187459	-1.8009634118
H	1.5528411644	0.0718360834	-2.2625763985
C	3.9357901946	-2.0148010555	-0.0555306051
C	3.1480944124	-2.6416563952	-1.0109235362
H	1.6867444437	-2.3687392958	-2.5689771728
H	4.6326342007	-2.5794258515	0.55532648
H	3.2210800001	-3.715126669	-1.1518283291
O	4.672191739	-0.023127563	1.0275174885
C	4.1628158688	0.0056329321	2.3587373719
H	4.8919917717	0.5524257805	2.9569698953
H	3.1927782073	0.5096622985	2.3923284513
H	4.0546793676	-1.0101606798	2.7549749976
C	-2.1960152688	0.4330517153	0.0361836955
O	-1.3626361037	-0.0991955915	0.9818631492
O	0.8141483786	0.2266123099	1.4784543948
N	-1.6601822803	1.2903734813	-0.7582196898

DMF

C	-0.3443266269	1.3495493449	-0.346921045
C	-4.0427411723	-1.0193948461	0.9464591614
C	-3.6046424882	-0.0458726937	0.0429460836
C	-4.5033235122	0.5057823983	-0.8779833836
C	-5.8232643328	0.084915579	-0.892049264
C	-6.2579948052	-0.8860913873	0.0089347464
C	-5.3671959007	-1.435645014	0.9254972756
H	-3.3469290566	-1.4455097057	1.6597679543
H	-4.1537164693	1.2602188199	-1.5736802047
H	-6.5180350404	0.5135318045	-1.6062894376
H	-7.2923961905	-1.2132484086	-0.0047578658
H	-5.7045634411	-2.190932617	1.6270331757
C	-0.1403431886	0.413688607	0.7734842497
C	1.9344956696	2.4374821031	-0.6179083709
H	2.208499617	3.4917385948	-0.6032892723
C	2.9491057394	1.5650430596	-0.4408661615
H	3.9093625376	1.9989651601	-0.1714082314
C	0.5345639704	2.2217992668	-0.8977006288

H	0.0857156663	2.9238989715	-1.5995778677
C	2.9785421046	0.1188769652	-0.6304767432
C	2.1945225119	-0.5403842622	-1.587140548
C	3.9064031318	-0.6466424015	0.0978994607
C	2.3044999097	-1.9068203264	-1.7903018716
H	1.5102990955	0.0389987173	-2.1971857816
C	4.0190130326	-2.0177982451	-0.1033504161
C	3.2159088823	-2.650650998	-1.0429914611
H	1.6935135645	-2.3916877941	-2.5442549361
H	4.7470390727	-2.5731216321	0.4788937272
H	3.3103205352	-3.7198141863	-1.2021879047
O	4.7485395364	-0.0343581719	0.9870700306
C	4.2010730442	0.1420763111	2.2926872217
H	4.9407750949	0.69962133	2.8671202012
H	3.261484406	0.7004662357	2.2496672521
H	4.0236420984	-0.8284671244	2.7687920453
C	-2.2231696771	0.4035946067	0.0467173482
O	-1.3858873173	-0.1609487467	0.9722290999
O	0.7935265598	0.1328881041	1.4677777956
N	-1.6833355619	1.2795765816	-0.7241994334

DMSO

C	-0.3443449743	1.3493837538	-0.3478966093
C	-4.042871894	-1.0180548694	0.9477677883
C	-3.6047158829	-0.0454177855	0.0433250172
C	-4.503365685	0.5054303748	-0.8781234801
C	-5.823337927	0.0846445528	-0.8917712606
C	-6.2581275759	-0.8854778614	0.0101429076
C	-5.3673592781	-1.434225679	0.9272205053
H	-3.3470702373	-1.4435410972	1.6614701878
H	-4.1537035372	1.2591737954	-1.5745503197
H	-6.51808818	0.5126221998	-1.6064203686
H	-7.292555795	-1.2125770214	-0.0032380852
H	-5.7047758371	-2.1888249113	1.6294793729
C	-0.1402831297	0.4141673022	0.7730249423
C	1.9345778148	2.4368561735	-0.6200368903
H	2.2086916293	3.4911012126	-0.6062943934
C	2.9491423223	1.5644623328	-0.4424054961
H	3.9094558095	1.9984937093	-0.1733143024
C	0.5345635792	2.221179461	-0.899367062
H	0.0856816111	2.9229503663	-1.6015577871
C	2.9784845595	0.1181668579	-0.6309470332

C	2.194215518	-0.5417818749	-1.5869485336
C	3.9064397402	-0.6468715436	0.0977947114
C	2.3040006258	-1.9083858655	-1.7890474402
H	1.5099026415	0.0371899789	-2.19729216
C	4.0188114191	-2.0182189838	-0.1023317161
C	3.215442927	-2.6517445805	-1.041292584
H	1.6927990914	-2.3937844119	-2.5424895931
H	4.7468968401	-2.5731177914	0.4802500066
H	3.3096888853	-3.7210503383	-1.1996557112
O	4.7488827807	-0.0339784888	0.9862476073
C	4.2019123648	0.1433973434	2.2919196418
H	4.9420348087	0.7009596381	2.8657972874
H	3.2625504103	0.7021860421	2.2489316346
H	4.0242165278	-0.8268010267	2.7686438129
C	-2.2232095172	0.4039242655	0.0466375087
O	-1.3858608823	-0.1601980205	0.972343244
O	0.7936759094	0.1336895285	1.4673345127
N	-1.6834164826	1.2793972622	-0.7248938625

Et_2O

C	-0.3317135852	1.3561189574	-0.3454312371
C	-4.0287975981	-1.0129259706	0.9498238161
C	-3.5891896106	-0.04159549	0.0452263088
C	-4.4850349652	0.5064584791	-0.8800708631
C	-5.8041355446	0.0838011236	-0.897525851
C	-6.2406149958	-0.8852860729	0.004285066
C	-5.3524489652	-1.4310170431	0.9252589415
H	-3.3334663447	-1.4350927318	1.6659970792
H	-4.131525916	1.2594275443	-1.5754092281
H	-6.4971379562	0.5096646533	-1.6152406891
H	-7.2745764854	-1.2139835956	-0.0121304506
H	-5.691380848	-2.1849502232	1.6276162946
C	-0.1267084677	0.4273218226	0.7828375719
C	1.9475030514	2.4390380105	-0.6216671406
H	2.2263721	3.4921473839	-0.6078164172
C	2.9567710177	1.5610755801	-0.443382022
H	3.9205366385	1.9869614783	-0.1732033006
C	0.54667661	2.2259212917	-0.9002698336
H	0.096372014	2.9240643026	-1.6053298375
C	2.9752220546	0.1147062116	-0.6321871412
C	2.1970191511	-0.5364334672	-1.5986767188
C	3.888016416	-0.6585428279	0.1062259192

C	2.2994116427	-1.9028458536	-1.8035855887
H	1.5245759385	0.0501133457	-2.2150238174
C	3.994107343	-2.0294905335	-0.0984771594
C	3.1977228515	-2.6541723003	-1.0485357733
H	1.6936359606	-2.3818735366	-2.5655020611
H	4.7128029205	-2.5900662479	0.4901453256
H	3.28684242	-3.7235399304	-1.2097592159
O	4.7220830329	-0.0548722476	1.0076076064
C	4.1548963309	0.1314929767	2.3027772384
H	4.8922424268	0.6821839676	2.8871437102
H	3.2212063221	0.6979479977	2.2437168369
H	3.9582177069	-0.8355335504	2.7787454876
C	-2.2080825071	0.4096273927	0.0515027536
O	-1.3739471422	-0.1483711784	0.9821689511
O	0.8049559494	0.1558053873	1.4809853181
N	-1.6694479668	1.2820388946	-0.7243838788

Gas phase

C	-0.3250919216	1.3788021161	-0.3770970639
C	-3.9898191578	-1.0029760487	0.9801077647
C	-3.560459518	-0.0486419913	0.053950447
C	-4.4607205431	0.4692968634	-0.8832732803
C	-5.7751508391	0.0332502906	-0.8908167582
C	-6.2021477825	-0.9187194974	0.0327076328
C	-5.3090512176	-1.4343434796	0.9653645731
H	-3.2872622513	-1.3993040158	1.7040621699
H	-4.10911109	1.2091549658	-1.5936396429
H	-6.472573538	0.4352707663	-1.6181367271
H	-7.2329255219	-1.2579363036	0.0239801031
H	-5.6410394744	-2.175315015	1.6848230967
C	-0.0986975074	0.4703608561	0.766905596
C	1.9388355695	2.4793136718	-0.6822420682
H	2.2126695247	3.534013665	-0.678447325
C	2.9492296859	1.6060386091	-0.4916406154
H	3.9124541062	2.0299398044	-0.2177310847
C	0.5382913688	2.2510279233	-0.9505946102
H	0.0713083216	2.9322083634	-1.6616016841
C	2.9600011659	0.1577487102	-0.6647254809
C	2.2025885697	-0.4951741894	-1.6458074564
C	3.8373834549	-0.6152950544	0.1130172303
C	2.2872246694	-1.8659065481	-1.8237525999
H	1.5587152872	0.0930921839	-2.2906115988

C	3.9260731474	-1.9911896934	-0.0658386131
C	3.1474828507	-2.6185444953	-1.0273932114
H	1.6981667133	-2.3481871288	-2.5966829661
H	4.6210148516	-2.5532007351	0.5493935011
H	3.2237142653	-3.6917766451	-1.1687722956
O	4.6460452519	0.001250206	1.0260503933
C	4.1287693459	0.0197004765	2.3528650199
H	4.8529647928	0.5670666708	2.9570268878
H	3.1545080826	0.5154011209	2.3855948279
H	4.024632246	-0.9989461919	2.7440628896
C	-2.1838149104	0.4173245423	0.0488005298
O	-1.3420511965	-0.1160473472	0.984164868
O	0.8398105624	0.2236184927	1.4605361287
N	-1.6629863642	1.2829480817	-0.7461525777

TMP

C	-0.3269987833	1.3582013552	-0.3468359541
C	-4.01619794	-1.0159348366	0.959081224
C	-3.5787447835	-0.0458256359	0.0526853955
C	-4.4753363013	0.4990341877	-0.873249092
C	-5.7935349043	0.0741761146	-0.8895084803
C	-6.2281678695	-0.8937523137	0.0140557175
C	-5.3390248369	-1.4361821811	0.935629199
H	-3.3185942275	-1.4344931041	1.6752086362
H	-4.1207406716	1.2510491802	-1.569100389
H	-6.487545306	0.4974861211	-1.6078510956
H	-7.2616404644	-1.2242120212	-0.0014605796
H	-5.6766949742	-2.1893520795	1.6394967061
C	-0.1154688777	0.4318147353	0.7839369742
C	1.9493240712	2.4433736066	-0.6319791971
H	2.229201431	3.4963280673	-0.6202240133
C	2.9574469148	1.5643731106	-0.4533308258
H	3.92301817	1.9870494301	-0.1843100767
C	0.5476428691	2.2289273341	-0.9056941063
H	0.0920037889	2.9241797011	-1.6102963684
C	2.9713175777	0.117768228	-0.6393703886
C	2.1970224029	-0.531105486	-1.6100929079
C	3.8764036347	-0.6572528648	0.1060177703
C	2.2961586745	-1.8977312496	-1.8131612156
H	1.530778704	0.0578953495	-2.2308638845
C	3.9796382229	-2.0283954574	-0.0977921933
C	3.1873731245	-2.6509627751	-1.0520692571

H	1.6938507067	-2.3755081461	-2.5786170555
H	4.6941091972	-2.5897198428	0.4951124685
H	3.2742930845	-3.7206917368	-1.2123118106
O	4.7051588903	-0.0555981136	1.0124103753
C	4.1300459106	0.133134741	2.3028881869
H	4.8671332644	0.6797278672	2.8917108027
H	3.1987064165	0.7027916746	2.2390933362
H	3.9249221499	-0.832789725	2.7778960931
C	-2.1983076331	0.4079462664	0.057101505
O	-1.3627518018	-0.1466429013	0.9871231849
O	0.8180596015	0.1658648458	1.4794510982
N	-1.664878433	1.2803525542	-0.7223237821

MCH

C	-0.328967894	1.3578009996	-0.3448980379
C	-4.016103353	-1.0214941065	0.9577036903
C	-3.5796139604	-0.0497820357	0.0525117291
C	-4.4768081681	0.4954057332	-0.8727005196
C	-5.7946182242	0.0693106637	-0.8894285456
C	-6.2282685946	-0.9002044078	0.0129437453
C	-5.3385491979	-1.4429747664	0.9337926054
H	-3.3181559591	-1.440372561	1.6732994181
H	-4.1231358916	1.2486701667	-1.5676634636
H	-6.4890766051	0.4928706702	-1.6071811791
H	-7.2614218816	-1.2316280898	-0.0029333711
H	-5.6754682344	-2.1973584395	1.6367106223
C	-0.116823752	0.4298868764	0.784425939
C	1.9462815178	2.4458148521	-0.6282503056
H	2.2249445554	3.4990598282	-0.6150625312
C	2.9554729039	1.5677772472	-0.4507193199
H	3.9204574387	1.9914345241	-0.1811591857
C	0.5448581962	2.2302413112	-0.9023778588
H	0.0886651204	2.9261556401	-1.6059546555
C	2.9714110749	0.1214292374	-0.6387557792
C	2.1975849235	-0.5273661603	-1.6099432743
C	3.8781009262	-0.6532549031	0.1050999977
C	2.2987394487	-1.8935821665	-1.8149160637
H	1.5300776087	0.061387736	-2.2295787713
C	3.9833124215	-2.0239862751	-0.1005421717
C	3.1915067255	-2.6464965506	-1.0552787928
H	1.696753844	-2.3712587667	-2.580685487

H	4.6988378046	-2.5850780587	0.491318985
H	3.2799505879	-3.7158787222	-1.2169637493
O	4.7064715363	-0.0516147641	1.0119395775
C	4.1312342606	0.1357309689	2.302634627
H	4.8675754077	0.6830393448	2.8916932321
H	3.199185981	0.7042534532	2.2390612113
H	3.9274822829	-0.8307308885	2.7771082151
C	-2.1996221366	0.4053132294	0.0574921454
O	-1.3634320509	-0.1499568325	0.9866948247
O	0.8169361722	0.1635203104	1.4796339546
N	-1.666793835	1.279239702	-0.7206154571

MeAc

C	-0.3377888546	1.3765817503	-0.3682380523
C	-4.0283003426	-0.9907529151	0.9504891432
C	-3.5913604777	-0.0312787426	0.0315991932
C	-4.4880147985	0.4968173657	-0.904929773
C	-5.8049018739	0.066533368	-0.9191818828
C	-6.2385191947	-0.8904639716	-0.0028608271
C	-5.3497144665	-1.4165703819	0.9292414079
H	-3.3337414694	-1.3984551195	1.6757195426
H	-4.1388721287	1.2404227509	-1.6124651128
H	-6.4981600516	0.4767299346	-1.645660078
H	-7.2705595199	-1.2251115514	-0.0167812149
H	-5.6862784426	-2.1609991824	1.6427377689
C	-0.1329650722	0.4617343523	0.7696377519
C	1.9381647842	2.4639932987	-0.6495073369
H	2.2114191504	3.5185927334	-0.6409018722
C	2.9504204663	1.5914062965	-0.4623933485
H	3.9104390621	2.02267003	-0.1893089636
C	0.5391687905	2.2427609678	-0.931241946
H	0.0906266259	2.931424714	-1.6466019464
C	2.9693859936	0.1433881529	-0.6413359316
C	2.1927386544	-0.5139628511	-1.6055932532
C	3.8712435841	-0.6274596424	0.1119570915
C	2.2801394208	-1.8848603594	-1.7871068138
H	1.5300797593	0.0695184682	-2.2354550577
C	3.9605946798	-2.0038822213	-0.0666564271
C	3.1615312973	-2.6351094356	-1.0105564786
H	1.6750714828	-2.3691158868	-2.5462653091
H	4.6688089506	-2.5660223622	0.5335066868
H	3.2384444928	-3.7080780495	-1.1529144423

O	4.7050932106	-0.0079316722	1.0037795506
C	4.2201562939	0.0162978215	2.3449387313
H	4.9575264121	0.5637653523	2.9320373867
H	3.250737865	0.5205484831	2.3963957962
H	4.1218030442	-1.0005274511	2.7403252703
C	-2.212784645	0.4273838414	0.0347665901
O	-1.3767402974	-0.1160007548	0.9736104225
O	0.7988860949	0.198886159	1.473271999
N	-1.6747974801	1.2924507106	-0.7496022649

THF

C	-0.3361882533	1.3544602219	-0.3468274411
C	-4.0322819436	-1.0152724276	0.950384393
C	-3.5941432293	-0.0433119103	0.0455100299
C	-4.4917386489	0.5051052399	-0.8781328919
C	-5.8109127715	0.0823125101	-0.8936374262
C	-6.245805451	-0.887338607	0.0085207188
C	-5.3559923722	-1.4335053156	0.927836693
H	-3.3363689117	-1.4382128222	1.6655079568
H	-4.1405814592	1.2585143556	-1.5741662337
H	-6.505097282	0.5084317124	-1.6099934223
H	-7.2797227465	-1.2161053778	-0.0063555086
H	-5.6936243297	-2.1877949904	1.6303740088
C	-0.131203344	0.4245778	0.7796684025
C	1.9426775585	2.4389795135	-0.6226527607
H	2.2197250345	3.4925042142	-0.6091504075
C	2.9538973178	1.5630523069	-0.4447715855
H	3.9164859997	1.9920607161	-0.175561463
C	0.5421592578	2.2246214123	-0.9013185357
H	0.0924346344	2.9234542499	-1.605983115
C	2.9760750213	0.1165509953	-0.6330867478
C	2.1953740345	-0.537954467	-1.5954842802
C	3.8941333227	-0.6537897547	0.1021346035
C	2.2999439779	-1.9046037366	-1.7987293426
H	1.5188254095	0.045790172	-2.2099614285
C	4.0018725038	-2.0250793607	-0.1000913088
C	3.2027093887	-2.6530926921	-1.0459165432
H	1.6920023908	-2.3860665943	-2.5573437631
H	4.723550218	-2.5837826322	0.4867301437
H	3.2931703091	-3.7225640861	-1.2054943318
O	4.7313382409	-0.0465365022	0.9987623612
C	4.1717430837	0.1357716453	2.2981149033

H	4.9098491757	0.6892541637	2.8786685227
H	3.2356241763	0.6989440767	2.2451725652
H	3.9827584435	-0.8326921169	2.7741651258
C	-2.2131557909	0.4079486979	0.0501475642
O	-1.3774568119	-0.1510560133	0.9794391182
O	0.801356959	0.1506769343	1.4768540374
N	-1.6744521124	1.2810724689	-0.7248766108

Water

C	-0.3442615765	1.3484756599	-0.3474046828
C	-4.0468080919	-1.0159167379	0.9426825379
C	-3.6069233982	-0.0424319983	0.0399535343
C	-4.5045339987	0.5112649986	-0.8808412729
C	-5.8251549863	0.0924923954	-0.8955286864
C	-6.2616507157	-0.8784496414	0.0047071515
C	-5.3719302628	-1.4300591187	0.9211202322
H	-3.3519729769	-1.4437091615	1.6559372348
H	-4.1537778849	1.2656296759	-1.5760362534
H	-6.5190601531	0.5226863547	-1.6096517156
H	-7.2965617434	-1.203945584	-0.009461019
H	-5.7106587198	-2.1852847359	1.6220554354
C	-0.1421991378	0.4111853751	0.7719313641
C	1.9359450457	2.4345590498	-0.6162209618
H	2.210477472	3.4886641117	-0.6014516872
C	2.9502093963	1.5617996544	-0.4385685994
H	3.910312451	1.9957820279	-0.1686897544
C	0.5360652108	2.2200785052	-0.8969844318
H	0.0885315482	2.92325096	-1.5986113347
C	2.9797917116	0.1156193924	-0.6279291827
C	2.1950205271	-0.5442292577	-1.5835999227
C	3.9085164844	-0.6494902199	0.0998777159
C	2.3050189567	-1.9107771268	-1.7862435647
H	1.5100883972	0.0346700904	-2.1932987328
C	4.0210085868	-2.0207778839	-0.1006566379
C	3.2171138528	-2.654203636	-1.0393051418
H	1.6934042097	-2.3960550157	-2.5394151553
H	4.7494730679	-2.5758299936	0.4813069101
H	3.3114487237	-3.7234441952	-1.1979970319
O	4.751606965	-0.0367377986	0.9879070288
C	4.2055030245	0.1405954112	2.2940038495
H	4.9458003856	0.6983379819	2.8674365842

H	3.2661329856	0.6994448538	2.2515720563
H	4.0284009936	-0.8296424066	2.770837039
C	-2.2247804314	0.4049087619	0.0444792258
O	-1.3885090564	-0.1617772685	0.9697083995
O	0.7911407642	0.1278235599	1.4661526342
N	-1.6832466265	1.2808169603	-0.7253171642

2. Charge-transfer state

Bu_2O

C	-0.2350834244	1.6202005209	0.2120808413
C	-3.9202904961	-0.9644500542	0.9834989986
C	-3.4577202186	0.1314906811	0.2523321744
C	-4.3229442277	0.7917417676	-0.6265152984
C	-5.6275590832	0.3593546535	-0.7673648672
C	-6.0843698515	-0.7334287772	-0.0367763423
C	-5.2296509008	-1.3921900844	0.8363310349
H	-3.2605681606	-1.4741117571	1.6597792354
H	-3.9644804024	1.6339552172	-1.1874708892
H	-6.2894381221	0.8698693843	-1.4433091214
H	-7.1003659725	-1.0672187978	-0.1486072978
H	-5.5812214709	-2.2360859314	1.4018073736
C	-0.0641982955	0.5832028725	1.2205933129
C	2.049463023	2.5653012022	-0.1940087679
H	2.5681263402	3.5084960971	-0.2558636569
C	2.8884195955	1.4205663211	-0.1326736028
H	3.8616708499	1.5759725358	0.2934995282
C	0.6748780558	2.6389255029	-0.1639609661
H	0.2227057143	3.5777632233	-0.4360333617
C	2.6147447296	0.1341753176	-0.6284074987
C	1.5311678786	-0.1532435966	-1.5007657143
C	3.4811609839	-0.9583997415	-0.2744383883
C	1.2829235942	-1.4371895425	-1.9480685936
H	0.9378714477	0.6564313028	-1.873085385
C	3.2155188926	-2.2325833831	-0.720289733
C	2.1120587608	-2.4714818022	-1.5477119102
H	0.46115431	-1.6253228454	-2.6145937211
H	3.8428037166	-3.0552503784	-0.4394068425
H	1.9261742917	-3.4744455713	-1.8881457914
C	-2.0879180975	0.5942707333	0.3949246237
O	-1.2781798908	-0.0566604376	1.2448517962
O	0.8135166541	0.2890274044	1.9718454094

N	-1.5295336214	1.5819348814	-0.2121977996
O	4.5119298861	-0.6425013409	0.5117727659
C	5.3921410658	-1.6400873414	0.974599827
H	6.1135848919	-1.131619296	1.5942864839
H	4.8621567139	-2.3769071285	1.5653198018
H	5.8998468398	-2.1224358114	0.1481683424

DMF

C	-0.22372000	1.66220600	0.04125000
C	-3.77857800	-1.08350100	0.96354500
C	-3.37733400	0.00207600	0.17398700
C	-4.28852800	0.56018200	-0.73428800
C	-5.56868800	0.04102100	-0.84732200
C	-5.96266200	-1.04050600	-0.06031200
C	-5.06328800	-1.59767400	0.84351600
H	-3.08015500	-1.52034300	1.66826300
H	-3.97653000	1.40035300	-1.34475700
H	-6.26576400	0.48047900	-1.55350200
H	-6.96519900	-1.44495300	-0.15215300
H	-5.36316900	-2.43921500	1.45952000
C	0.01695400	0.69431500	1.07470800
C	2.01321600	2.68592800	-0.51679900
H	2.46730700	3.66699900	-0.66193400
C	2.91564500	1.63925100	-0.37482000
H	3.90096500	1.88151400	0.00947900
C	0.61926800	2.69424700	-0.42068100
H	0.11747700	3.62875300	-0.66361100
C	2.71958100	0.25006600	-0.73367400
C	1.69067200	-0.21975500	-1.53122700
C	3.61023700	-0.74697800	-0.27001500
C	1.50071300	-1.60869200	-1.80641500
H	1.04025900	0.48993500	-2.02672900
C	3.43354100	-2.13635500	-0.54916300
C	2.36770500	-2.57440900	-1.30743800
H	0.66622400	-1.89772900	-2.43560400
H	4.16448400	-2.83418000	-0.15583400
H	2.22732600	-3.62493600	-1.52500000
O	4.66934400	-0.36742600	0.43892400
C	4.76697300	-0.80484500	1.80662200
H	5.66607800	-0.33872700	2.20107800
H	3.88026100	-0.46034300	2.34166200
H	4.84922200	-1.89327400	1.86829500

C	-2.03939300	0.54946700	0.28122000
O	-1.18532000	-0.01490400	1.17507600
O	0.92625900	0.42794900	1.82016700
N	-1.53213400	1.53492100	-0.38552300

DMSO

C	-0.22372000	1.66220600	0.04125000
C	-3.77857800	-1.08350100	0.96354500
C	-3.37733400	0.00207600	0.17398700
C	-4.28852800	0.56018200	-0.73428800
C	-5.56868800	0.04102100	-0.84732200
C	-5.96266200	-1.04050600	-0.06031200
C	-5.06328800	-1.59767400	0.84351600
H	-3.08015500	-1.52034300	1.66826300
H	-3.97653000	1.40035300	-1.34475700
H	-6.26576400	0.48047900	-1.55350200
H	-6.96519900	-1.44495300	-0.15215300
H	-5.36316900	-2.43921500	1.45952000
C	0.01695400	0.69431500	1.07470800
C	2.01321700	2.68592800	-0.51679900
H	2.46730700	3.66699900	-0.66193400
C	2.91564500	1.63925100	-0.37482000
H	3.90096500	1.88151400	0.00947900
C	0.61926800	2.69424800	-0.42068100
H	0.11747700	3.62875400	-0.66361100
C	2.71958100	0.25006600	-0.73367400
C	1.69067200	-0.21975500	-1.53122700
C	3.61023700	-0.74697800	-0.27001500
C	1.50071300	-1.60869200	-1.80641500
H	1.04025900	0.48993600	-2.02672900
C	3.43354000	-2.13635500	-0.54916300
C	2.36770500	-2.57440900	-1.30743900
H	0.66622400	-1.89772900	-2.43560400
H	4.16448300	-2.83418000	-0.15583400
H	2.22732500	-3.62493600	-1.52500000
O	4.66934400	-0.36742600	0.43892400
C	4.76697300	-0.80484500	1.80662200
H	5.66607800	-0.33872700	2.20107800
H	3.88026100	-0.46034300	2.34166200
H	4.84922200	-1.89327400	1.86829500
C	-2.03939300	0.54946700	0.28122000
O	-1.18532000	-0.01490400	1.17507600

O	0.92625900	0.42794900	1.82016700
N	-1.53213400	1.53492100	-0.38552300

Et_2O

C	-0.32989000	1.44629600	-0.14719400
C	-3.94420300	-1.24861400	0.66152600
C	-3.57871300	-0.00344100	0.13955300
C	-4.56098800	0.85412200	-0.37058400
C	-5.89108800	0.46634700	-0.35761000
C	-6.25329100	-0.77541400	0.16253500
C	-5.27944100	-1.62943400	0.67053100
H	-3.18395800	-1.91195500	1.05718000
H	-4.26763800	1.81735600	-0.77246800
H	-6.65045500	1.13222700	-0.75327500
H	-7.29584600	-1.07577500	0.17125900
H	-5.56021200	-2.59560300	1.07567800
C	-0.03030600	0.15574100	0.49742700
C	1.94280200	2.58810400	-0.31368400
H	2.27171400	3.61964300	-0.19380900
C	2.93047900	1.66701200	-0.37624000
H	3.94473800	2.05662300	-0.32808100
C	0.50936600	2.47141000	-0.44777500
H	-0.00053700	3.38207200	-0.76004100
C	2.85848000	0.22380900	-0.57745400
C	1.85414100	-0.39269200	-1.33701100
C	3.89496100	-0.58652400	-0.08293100
C	1.86181300	-1.76106600	-1.56887000
H	1.07337900	0.22018400	-1.77351000
C	3.90533200	-1.95950300	-0.31320900
C	2.88730200	-2.54910300	-1.05141600
H	1.07821900	-2.21150900	-2.16873100
H	4.72341000	-2.54976500	0.08645900
H	2.90151100	-3.61844300	-1.23440200
O	4.93915200	-0.02041300	0.59674600
C	4.70798000	0.15667500	1.99366600
H	5.57999700	0.67702100	2.38951000
H	3.80638900	0.75116200	2.16753700
H	4.60364100	-0.81358800	2.49141400
C	-2.18778600	0.41491100	0.11942300
O	-1.26632300	-0.45557500	0.63686000
O	0.97139700	-0.36782400	0.89913800
N	-1.70855100	1.51986300	-0.33154000

Gas phase

C	0.19765500	1.79578200	0.20846700
C	-3.29143700	-1.03176100	0.99690300
C	-2.90206400	0.08591100	0.24877700
C	-3.82037800	0.67495100	-0.63094400
C	-5.09693500	0.15269800	-0.75691900
C	-5.48040000	-0.96057400	-0.01159500
C	-4.57315800	-1.54715500	0.86445800
H	-2.58251600	-1.48738900	1.67885200
H	-3.51132300	1.54221700	-1.20404100
H	-5.80144900	0.61591400	-1.44036400
H	-6.48156300	-1.36660900	-0.11306400
H	-4.86580300	-2.41335500	1.44946000
C	0.44085500	0.84108700	1.22712800
C	2.47623400	2.62771100	-0.16947300
H	3.16992900	3.46301300	-0.19809300
C	3.02564600	1.27214700	-0.06184000
H	3.87916800	1.08942400	0.58310200
C	1.16461200	2.86712200	-0.06403500
H	0.81222200	3.89612300	-0.05966200
C	2.49408000	0.22345300	-0.76206000
C	1.34733100	0.43000500	-1.60958700
C	2.90201200	-1.12419400	-0.47683400
C	0.61655400	-0.66125100	-2.07863900
H	1.17951200	1.41282800	-2.02437000
C	2.18060400	-2.17936000	-0.97417400
C	1.02911500	-1.93842000	-1.75721900
H	-0.24266000	-0.50778000	-2.72162700
H	2.48615500	-3.19626200	-0.75812000
H	0.47617200	-2.78834300	-2.14516900
O	3.98336800	-1.26256300	0.31731900
C	3.84691900	-2.17919900	1.40926700
H	4.76756200	-2.09077400	1.98451400
H	2.98875500	-1.89167700	2.02337700
H	3.74380200	-3.21125300	1.06185000
C	-1.56920600	0.63730300	0.36980600
O	-0.70501700	0.05054100	1.24042000
O	1.42183900	0.62638500	1.92372800
N	-1.07824400	1.67164100	-0.25675600

TMP

C	-0.20188600	1.53443100	0.22644000

C	-3.78192300	-1.30129100	0.68331500
C	-3.38782400	-0.05331300	0.17851900
C	-4.33780200	0.75565900	-0.46505500
C	-5.64578900	0.32142600	-0.59925800
C	-6.03224500	-0.92104000	-0.09752800
C	-5.09488800	-1.72681500	0.54304900
H	-3.05222600	-1.92647500	1.18519300
H	-4.02627700	1.72016500	-0.85005300
H	-6.37312700	0.95452300	-1.09747000
H	-7.05835900	-1.25724600	-0.20444400
H	-5.39019500	-2.69368500	0.93794400
C	0.08539400	0.31030600	0.94649300
C	2.02514600	2.78162800	0.06621300
H	2.38545200	3.79225500	0.25732900
C	3.00705200	1.84512600	-0.13221500
H	4.03660100	2.12471200	0.06626000
C	0.61579400	2.66178600	0.02524800
H	0.05927900	3.57991600	-0.14678200
C	2.78298600	0.50149000	-0.67515100
C	1.98261000	0.28859200	-1.77165800
C	3.42429300	-0.63387300	-0.12749300
C	1.73642300	-1.01822600	-2.29467400
H	1.51403100	1.14272300	-2.24648500
C	3.18912000	-1.93336100	-0.64023700
C	2.34797600	-2.12147300	-1.73118100
H	1.04502500	-1.13146700	-3.12201800
H	3.63570600	-2.79254400	-0.15500000
H	2.15981800	-3.12031200	-2.10487500
O	4.26124600	-0.38996200	0.86872300
C	4.85134700	-1.46292200	1.59185600
H	5.40476700	-0.99588200	2.40336700
H	4.08108700	-2.11914300	2.00388900
H	5.53978700	-2.02875600	0.95761300
C	-2.02740200	0.41036300	0.31135600
O	-1.12882500	-0.38685600	0.94149000
O	1.06099200	-0.15319000	1.52551700
N	-1.53413900	1.53718600	-0.11407900

MCH

C	-0.16023300	1.55163300	0.23990200
C	-3.72713600	-1.30028000	0.68125400
C	-3.33879800	-0.04528400	0.18831000

C	-4.29314100	0.76900500	-0.44340600
C	-5.59973700	0.33288700	-0.57757700
C	-5.98050400	-0.91638400	-0.08774600
C	-5.03845900	-1.72737300	0.54109800
H	-2.99392800	-1.92896100	1.17355600
H	-3.98570800	1.73853500	-0.81907500
H	-6.33059000	0.96921100	-1.06623200
H	-7.00603400	-1.25423200	-0.19468700
H	-5.32973600	-2.69923200	0.92635800
C	0.13003900	0.32054500	0.94460400
C	2.06517800	2.78180500	0.08945300
H	2.44474600	3.78390500	0.28843800
C	3.02178100	1.82095100	-0.11969500
H	4.06062800	2.06751900	0.07636200
C	0.66064400	2.68081600	0.04840600
H	0.11144200	3.60526400	-0.11389700
C	2.74827100	0.49512400	-0.67007300
C	1.92168300	0.32102000	-1.76589600
C	3.35878300	-0.66038200	-0.14105500
C	1.62522700	-0.96739800	-2.29898000
H	1.47661500	1.19587300	-2.22667500
C	3.07238400	-1.93891400	-0.66497200
C	2.20613500	-2.08840100	-1.75390700
H	0.92090100	-1.05005100	-3.11899000
H	3.49603300	-2.81784700	-0.19488300
H	1.98059200	-3.07679700	-2.13517000
O	4.22052100	-0.45334100	0.85376700
C	4.78982600	-1.55342700	1.54994200
H	5.37102400	-1.11570600	2.35856400
H	4.00733800	-2.19305700	1.96589100
H	5.44981600	-2.13016500	0.89516300
C	-1.98050700	0.42006200	0.32100300
O	-1.08018500	-0.38080100	0.93782700
O	1.09322300	-0.13735600	1.50496100
N	-1.49003100	1.55738600	-0.09588100

MeAc

C	-0.58283800	1.08034100	-0.21379500
C	-4.52359300	-0.98083300	0.96542000
C	-3.96495300	-0.05534400	0.07261300
C	-4.77724500	0.55603300	-0.89505600
C	-6.12428000	0.24296200	-0.96446600

C	-6.67686200	-0.67888100	-0.07483000
C	-5.87421500	-1.28771200	0.88721200
H	-3.89811500	-1.45326300	1.71375300
H	-4.33774200	1.27097600	-1.58135400
H	-6.74966700	0.71724900	-1.71324500
H	-7.73288300	-0.92138300	-0.13249300
H	-6.30323600	-2.00427500	1.57937100
C	-0.51202100	0.15127900	0.93888400
C	1.73280600	2.23382800	-0.73138800
H	1.90200100	3.30203200	-0.85745500
C	2.91390800	1.53939500	-0.63131000
H	3.79829600	2.17157200	-0.61599300
C	0.32013600	1.95198800	-0.80537200
H	-0.20792400	2.68908100	-1.40744700
C	3.26501500	0.13711900	-0.66862100
C	2.57480800	-0.81134200	-1.45544100
C	4.44179200	-0.29191900	-0.00623000
C	3.00484200	-2.12190700	-1.54073500
H	1.71254600	-0.49261400	-2.02891200
C	4.86885800	-1.60861500	-0.08895800
C	4.14992300	-2.52688300	-0.84934100
H	2.46305400	-2.82879300	-2.15963500
H	5.77156400	-1.89914500	0.43782000
H	4.49317800	-3.55380100	-0.91841900
O	5.19437200	0.60733000	0.69202400
C	4.77226900	0.81794600	2.04010400
H	5.40962100	1.60309900	2.44589300
H	3.72481700	1.13174000	2.07638000
H	4.89839900	-0.09709800	2.62850000
C	-2.55927800	0.28067000	0.13412600
O	-1.80722900	-0.32460500	1.09382900
O	0.32885600	-0.17662900	1.73674600
N	-1.90947600	1.10672800	-0.61778100

THF

C	-0.58775600	1.06996700	-0.21850100
C	-4.53603800	-0.96971600	0.97233100
C	-3.97299800	-0.05552600	0.07103600
C	-4.78079900	0.54664200	-0.90566600
C	-6.12828500	0.23560500	-0.97564200
C	-6.68547200	-0.67504700	-0.07754000
C	-5.88709600	-1.27469100	0.89352000

H	-3.91360000	-1.43490900	1.72771100
H	-4.33744700	1.25294500	-1.59841200
H	-6.75041300	0.70283700	-1.73155300
H	-7.74184800	-0.91597000	-0.13568700
H	-6.31967600	-1.98259300	1.59236400
C	-0.52280300	0.15243300	0.94565300
C	1.72990500	2.21929800	-0.74814200
H	1.89579000	3.28676900	-0.88368700
C	2.91430900	1.53095200	-0.64321600
H	3.79540300	2.16775800	-0.63294300
C	0.31623300	1.93433300	-0.81825500
H	-0.21229400	2.66474400	-1.42785100
C	3.27470500	0.13093900	-0.67039000
C	2.59149900	-0.82853400	-1.45265600
C	4.45312100	-0.28419400	-0.00333500
C	3.03028200	-2.13494300	-1.52806400
H	1.72806200	-0.51977200	-2.02973100
C	4.88879400	-1.59682500	-0.07624900
C	4.17677200	-2.52593300	-0.83190800
H	2.49468800	-2.85014700	-2.14281200
H	5.79273000	-1.87731900	0.45388100
H	4.52758100	-3.55089000	-0.89315100
O	5.19829000	0.62629000	0.69032700
C	4.77158000	0.84180400	2.03561300
H	5.40180100	1.63461700	2.43787700
H	3.72149900	1.14723200	2.06771000
H	4.90354900	-0.06848500	2.63023800
C	-2.56667400	0.27838000	0.13293400
O	-1.81930600	-0.31822200	1.10190900
O	0.31271000	-0.16894800	1.75009200
N	-1.91357900	1.09436600	-0.62596000

Water

C	-0.2694536672	1.6098926007	0.2334286082
C	-3.9863061098	-0.9390568206	0.9825523739
C	-3.5087063456	0.1551464708	0.2565801544
C	-4.3630448466	0.8260927327	-0.6262366122
C	-5.670760197	0.4060982941	-0.7762641022
C	-6.1418432464	-0.6848544165	-0.0510703914
C	-5.2985257659	-1.3543208293	0.8260181228
H	-3.3372691985	-1.4587143144	1.6614611675
H	-3.9960246189	1.6663497397	-1.1844487641

H	-6.3235218845	0.923979548	-1.4551150002
H	-7.1597383742	-1.0093190614	-0.1705993477
H	-5.6612904997	-2.1966949066	1.386247232
C	-0.1200721569	0.5704293157	1.2432189987
C	2.0308914848	2.5322542225	-0.1755311859
H	2.5466815165	3.4765177811	-0.2413399218
C	2.8816352732	1.3964383898	-0.124679848
H	3.8620813133	1.5725488508	0.2767597255
C	0.6557941107	2.6134823869	-0.1451166415
H	0.2148306283	3.5561663037	-0.4218328869
C	2.6231535428	0.0998002827	-0.6068617466
C	1.5434604072	-0.2139295376	-1.4754703067
C	3.5198960779	-0.9713323395	-0.2625493812
C	1.3310213115	-1.4983867449	-1.9324672763
H	0.9190273153	0.5795631412	-1.8319452243
C	3.2910476696	-2.2506248555	-0.7201952536
C	2.195232112	-2.5137943946	-1.5469477725
H	0.5127085923	-1.7051304888	-2.597518587
H	3.9441350763	-3.0563304541	-0.4494997946
H	2.0382536749	-3.5179040389	-1.8977005397
C	-2.1373566304	0.6037993589	0.407563382
O	-1.3370350309	-0.0540580937	1.2635193327
O	0.7504435355	0.2649445538	2.0036993576
N	-1.5596155204	1.5843499364	-0.197419465
O	4.5381187468	-0.6357320426	0.5317503719
C	5.4890376989	-1.6020900954	0.9281585573
H	6.2022366223	-1.0766056506	1.5426346007
H	5.0203008676	-2.3880026576	1.5063096566
H	5.9910725151	-2.0239061668	0.0669044078

Isomer *E (trans)*

1. Ground state

Bu_2O

C	-0.9461425853	-0.8637220664	0.0360815518
C	-5.011184868	1.2486529732	0.0094072522
C	-4.412449221	-0.0148882045	0.0233120804
C	-5.2118588147	-1.1638236461	0.0319428249
C	-6.5919801416	-1.0457454806	0.0266466359
C	-7.1871850154	0.2145438804	0.0126827404
C	-6.395224743	1.3579606418	0.0041393231

H	-4.3902551288	2.1370210561	0.0026020788
H	-4.7346273885	-2.1372468811	0.0425115444
H	-7.2091594953	-1.9379495885	0.0332417434
H	-8.2686205933	0.3034784577	0.0084404194
H	-6.8567786636	2.3396205035	-0.0067772496
C	-0.8968414239	0.6034132936	0.0227514108
C	1.4781356785	-1.7219981518	0.0378004611
H	1.9561431587	-2.6978265657	0.0493119899
C	2.2622834929	-0.6111456154	0.0204153508
H	1.784397445	0.3616954709	0.0164985891
C	0.0527494282	-1.797604529	0.0438781131
H	-0.3420006301	-2.8116970195	0.0556475597
C	3.7106862808	-0.6028639416	0.0077369078
C	4.4929965215	-1.7666066115	-0.0271506332
C	4.3854402727	0.6449079457	0.0280670679
C	5.8757330922	-1.7188311807	-0.0387673455
H	4.001512908	-2.7330820911	-0.0496102896
C	5.7795763515	0.6941706679	0.0165718603
C	6.5159357317	-0.4826925565	-0.0160463069
H	6.4532619074	-2.6359831206	-0.0669108265
H	6.2954846793	1.6457593576	0.033060492
H	7.5997861299	-0.4293531583	-0.0250005196
O	3.6060153275	1.7414196186	0.0595439447
C	4.2176264262	3.0172094512	0.0776773937
H	3.4005032197	3.7362469985	0.0985544756
H	4.838924965	3.1467973839	0.970041805
H	4.8226700291	3.1802324879	-0.820343179
C	-2.9665774688	-0.1539818765	0.0279422574
O	-2.2254178287	0.9961114311	0.0175264514
O	-0.0122677495	1.4159395511	0.0172664636
N	-2.2853342862	-1.2441748852	0.0396155616

DMF

C	-0.9460522504	-0.8636065946	0.0374616446
C	-5.0136983032	1.2466189263	0.0047254248
C	-4.41560613	-0.0177085436	0.0222928536
C	-5.2166125073	-1.1661234267	0.034646531
C	-6.5969394512	-1.0468956135	0.0294141323
C	-7.1911000497	0.2142290436	0.0118891225
C	-6.3978884153	1.3572418035	-0.0003749125
H	-4.394148856	2.1358868609	-0.0049654813
H	-4.7439054245	-2.1417297668	0.0479617975

H	-7.2147031378	-1.9385330744	0.0388309471
H	-8.2723527809	0.3040457038	0.0077377493
H	-6.8583391542	2.3392749964	-0.0140749802
C	-0.9009369204	0.6008065884	0.0178269581
C	1.4790334965	-1.7228753634	0.04153461
H	1.9559705916	-2.6988310242	0.0557114268
C	2.263178192	-0.6108529734	0.0207847159
H	1.7831569729	0.3606701983	0.0151272066
C	0.0544232265	-1.797428447	0.0485882354
H	-0.3376294898	-2.812447543	0.0644456752
C	3.710603104	-0.6003209723	0.0067816276
C	4.4915297043	-1.7663884978	-0.0313182155
C	4.3863635432	0.6476197557	0.0287727113
C	5.8739650666	-1.7193248477	-0.0443878887
H	3.9996612202	-2.7325421646	-0.0555147966
C	5.7808201668	0.6953125435	0.0158923603
C	6.5152247637	-0.4827468084	-0.0198486768
H	6.4509144755	-2.636705282	-0.0752169879
H	6.2990162788	1.6455135015	0.0337107181
H	7.5990267136	-0.4300736041	-0.0298818869
O	3.609671443	1.7469346047	0.063577499
C	4.2297672056	3.0226720177	0.0850594973
H	3.4169633979	3.7464086555	0.109015288
H	4.8520049242	3.1447362349	0.9769049996
H	4.8341814847	3.1838043391	-0.8128061512
C	-2.9699834519	-0.1575553073	0.0269382096
O	-2.2269759969	0.9926649122	0.0112167356
O	-0.0170501603	1.4180044348	0.0087328764
N	-2.285596492	-1.2457912661	0.0431184241

DMSO

C	-0.9460522503	-0.8636065949	0.0374616441
C	-5.0136983028	1.2466189264	0.0047254253
C	-4.4156061298	-0.0177085436	0.0222928536
C	-5.2166125073	-1.1661234266	0.0346465307
C	-6.5969394511	-1.0468956132	0.0294141322
C	-7.1911000495	0.214229044	0.0118891228
C	-6.397888415	1.3572418038	-0.000374912
H	-4.3941488556	2.135886861	-0.0049654806
H	-4.7439054246	-2.1417297668	0.0479617969
H	-7.2147031379	-1.938533074	0.0388309467
H	-8.2723527806	0.3040457043	0.0077377497

H	-6.8583391537	2.3392749968	-0.0140749793
C	-0.9009369202	0.6008065881	0.017826958
C	1.4790334965	-1.7228753638	0.0415346097
H	1.9559705916	-2.6988310246	0.0557114264
C	2.263178192	-0.6108529737	0.0207847157
H	1.7831569729	0.3606701979	0.0151272064
C	0.0544232266	-1.7974284473	0.0485882349
H	-0.3376294898	-2.8124475433	0.0644456745
C	3.7106031039	-0.6003209724	0.0067816277
C	4.4915297045	-1.7663884979	-0.0313182152
C	4.386363543	0.6476197557	0.0287727114
C	5.8739650668	-1.7193248475	-0.0443878882
H	3.9996612205	-2.7325421647	-0.0555147961
C	5.7808201666	0.6953125437	0.0158923605
C	6.5152247636	-0.4827468082	-0.0198486764
H	6.4509144758	-2.6367052817	-0.0752169872
H	6.2990162784	1.6455135017	0.0337107181
H	7.5990267136	-0.4300736037	-0.0298818864
O	3.6096714426	1.7469346046	0.0635774987
C	4.229767205	3.0226720177	0.0850594969
H	3.4169633971	3.7464086553	0.1090152874
H	4.8520049234	3.1447362352	0.9769049993
H	4.8341814841	3.1838043391	-0.8128061515
C	-2.9699834518	-0.1575553074	0.0269382094
O	-2.2269759967	0.992664912	0.0112167357
O	-0.01705016	1.4180044345	0.0087328766
N	-2.2855964919	-1.2457912663	0.0431184235

Et_2O

C	-0.9461547261	-0.8636433614	0.0381612053
C	-5.0119074465	1.2480160838	0.0077902608
C	-4.4133693954	-0.0157392129	0.0233018371
C	-5.2132250872	-1.164512316	0.0326561789
C	-6.5933941371	-1.0461130111	0.0264790862
C	-7.1882901105	0.2144007066	0.0109108525
C	-6.3959785136	1.3576927829	0.0016488976
H	-4.3913328263	2.1366175178	0.0004045448
H	-4.7371797367	-2.1384971909	0.0444515695
H	-7.2107447636	-1.9381624785	0.0336136349
H	-8.2696766373	0.3035844793	0.0059658091
H	-6.8572114138	2.3394616745	-0.0105208438
C	-0.8980089002	0.602798483	0.0232803615

C	1.4783987708	-1.7219594994	0.0404698112
H	1.9561972664	-2.6977716591	0.0532217807
C	2.2625521078	-0.6108246661	0.0211467577
H	1.7842151627	0.3617313958	0.016480745
C	0.0532285507	-1.7974062893	0.0470052008
H	-0.340719306	-2.8117816745	0.0600202026
C	3.7107069569	-0.6021179582	0.0073340538
C	4.492556221	-1.7665009361	-0.0292547723
C	4.3857709583	0.6456579677	0.0279977765
C	5.8752170077	-1.7190042202	-0.0420520827
H	4.0008870964	-2.7328497495	-0.0523198756
C	5.7800129595	0.6943980731	0.0153521236
C	6.515778997	-0.4827888032	-0.0188471604
H	6.4525276252	-2.6362386038	-0.0715680551
H	6.2965455261	1.6456075332	0.0321185627
H	7.5996202268	-0.4297020139	-0.0287190202
O	3.6071457276	1.7428321039	0.061035118
C	4.2210100348	3.0185880421	0.0803611585
H	3.4050673844	3.7388506332	0.1027828451
H	4.8429642449	3.1457137108	0.9723824916
H	4.825436318	3.1815101931	-0.8178552347
C	-2.9675569386	-0.155000518	0.0287467547
O	-2.2259908441	0.9951575855	0.0172298352
O	-0.0137172114	1.4166217226	0.0172711388
N	-2.2854241484	-1.2446625268	0.0418264504

Gas phase

C	-0.9461554446	-0.8637742301	0.0152506921
C	-5.007954991	1.2519073532	0.0358844897
C	-4.4075816434	-0.0102152464	0.0248639059
C	-5.2041729772	-1.1604585928	0.015705606
C	-6.5841261368	-1.0446428827	0.0175920312
C	-7.1815312225	0.2141982284	0.0285788701
C	-6.3919128133	1.3586736335	0.0376920492
H	-4.3856096436	2.1393409839	0.042947253
H	-4.7209174024	-2.1310383024	0.0072113278
H	-7.2000516596	-1.9378286422	0.0104841434
H	-8.2632184608	0.3012355629	0.0300259353
H	-6.8556211453	2.3394471971	0.046248781
C	-0.8907288957	0.6065571578	0.0277138815
C	1.4763921494	-1.7223083838	0.0059590937
H	1.9555668685	-2.6981044076	-0.0028088879

C	2.2600715957	-0.6125893089	0.0148733399
H	1.7835331007	0.3611373703	0.0236502369
C	0.0499506404	-1.7986779007	0.0064438294
H	-0.3487925488	-2.8113158233	-0.0019337177
C	3.7097581068	-0.6060476731	0.0137384422
C	4.4939504084	-1.7672888555	0.0032324388
C	4.3836167303	0.6413110685	0.0237807961
C	5.8770389453	-1.7184627102	0.0025217333
H	4.0030558589	-2.7343266722	-0.0046038124
C	5.7770290777	0.6930023396	0.0230457175
C	6.5159237886	-0.4828387634	0.0124804102
H	6.455391655	-2.6355326892	-0.0057181646
H	6.290580836	1.6461519423	0.0306354103
H	7.599774018	-0.4285444859	0.0120284919
O	3.6003134688	1.7360105307	0.0336725286
C	4.2025182595	3.0112145431	0.0446671466
H	3.3805048976	3.7255217188	0.05177904
H	4.8167892292	3.1553268087	0.9408007641
H	4.8152936126	3.1714880444	-0.849749709
C	-2.9614380353	-0.1478494533	0.0227313157
O	-2.2219866707	1.0015684701	0.0319492346
O	-0.0043566654	1.4125084428	0.0339570339
N	-2.284939891	-1.2407923725	0.0129783218

TMP

C	-0.9462140533	-0.8637264436	0.0280914907
C	-5.0101615545	1.2498013161	0.0182213126
C	-4.41088734	-0.0133135277	0.0234418269
C	-5.2093470686	-1.1626909009	0.0265028945
C	-6.5894063609	-1.0453516025	0.0243502351
C	-7.185344918	0.2144958268	0.0191302589
C	-6.3941681614	1.358278258	0.0161057658
H	-4.3887284596	2.1378611681	0.0157515444
H	-4.7299703689	-2.1351319915	0.0304213728
H	-7.2061616732	-1.9379214261	0.0266418853
H	-8.266876398	0.3028292779	0.017394782
H	-6.8564397874	2.3396960495	0.0120186002
C	-0.8948497386	0.6046006764	0.0221453156
C	1.4775557805	-1.7219300001	0.0272540451
H	1.9559256785	-2.6978058148	0.0331460097
C	2.261686487	-0.6115239111	0.0178618137
H	1.784497072	0.3617676308	0.017221493

C	0.0517947848	-1.7978715986	0.0312267107
H	-0.3443097738	-2.8115114132	0.0371464977
C	3.7105790677	-0.6040367965	0.0095056048
C	4.4935898193	-1.7668928573	-0.0173115305
C	4.3849567025	0.6436335097	0.0269022126
C	5.876475395	-1.7187662871	-0.0248069424
H	4.0024096765	-2.7336339765	-0.0354264953
C	5.7788847973	0.6937057002	0.0194150557
C	6.5161754389	-0.4827890872	-0.005860481
H	6.4543225346	-2.6359160964	-0.0463863217
H	6.2938547653	1.6459068688	0.0333164999
H	7.6000471529	-0.4291380568	-0.0116429818
O	3.6041593397	1.7393365475	0.0512300135
C	4.2121964651	3.0150487832	0.067184691
H	3.3931876269	3.7322281445	0.0834537356
H	4.831004813	3.1495424905	0.9609587502
H	4.8198273523	3.1773908232	-0.8296221726
C	-2.964909684	-0.1519529518	0.0252194723
O	-2.2244257435	0.9980002596	0.0202506284
O	-0.0097202679	1.4148649226	0.019559001
N	-2.2852523983	-1.2431195134	0.0302974058

MCH

C	-0.9462033672	-0.863737526	0.0296215756
C	-5.0102559842	1.2496633719	0.0162838555
C	-4.411061431	-0.0135098426	0.0233118322
C	-5.2096486213	-1.1628234172	0.0276626841
C	-6.5897110513	-1.045375764	0.0249883899
C	-7.1855469695	0.2145298689	0.0179563904
C	-6.3942652733	1.3582568845	0.0136528517
H	-4.388860159	2.1377463447	0.0128125427
H	-4.7305195888	-2.1353795182	0.0329689015
H	-7.206532403	-1.937895071	0.0282781743
H	-8.2670699623	0.3029441259	0.0158078935
H	-6.856439692	2.3397116839	0.0081555641
C	-0.8950983735	0.6044606401	0.0218630134
C	1.4776270997	-1.7219384239	0.0297020724
H	1.9559578488	-2.6978055915	0.0371546933
C	2.2617576894	-0.6114874569	0.0183200002
H	1.784512257	0.3617651583	0.0167165224
C	0.051906475	-1.7978429654	0.0340462335
H	-0.3440410715	-2.8115351586	0.0414537636

C	3.7105989704	-0.6039135347	0.0090514184
C	4.4935385837	-1.7668456022	-0.0194724179
C	4.3850145756	0.6437691861	0.027038496
C	5.8764042009	-1.7187438348	-0.0278479449
H	4.0023300914	-2.7335533125	-0.0385301556
C	5.7789684065	0.6937657596	0.0186394384
C	6.5161603892	-0.4827533221	-0.0081510165
H	6.454216537	-2.6358840818	-0.0508088924
H	6.2940306628	1.6459044481	0.0330180924
H	7.6000302709	-0.4291362511	-0.0146526732
O	3.6043664441	1.7395440577	0.0528497026
C	4.2127743105	3.0152688667	0.0697130811
H	3.3939551362	3.7326138808	0.0872707663
H	4.8321709881	3.1486029503	0.963210355
H	4.8198027522	3.1783264376	-0.8273321071
C	-2.9651009785	-0.1522168058	0.0255911026
O	-2.2245374675	0.9977663978	0.0192674455
O	-0.0100170027	1.4149650537	0.0184823631
N	-2.2852572931	-1.2432636363	0.0322159917

MeAc

C	-0.9460435335	-0.863657249	0.0383189013
C	-5.0134138657	1.2467999699	0.0051663715
C	-4.4153096218	-0.017476885	0.0225808152
C	-5.21621356	-1.1659095094	0.0342075947
C	-6.5965160021	-1.0467423216	0.0284045622
C	-7.1907226984	0.2143263883	0.0110015696
C	-6.3975838267	1.3573500965	-0.0005324453
H	-4.3937157734	2.1359706296	-0.003969002
H	-4.7431278934	-2.1413275991	0.0474198722
H	-7.2142560629	-1.9384131765	0.0372657525
H	-8.2719861957	0.3040988935	0.0063791136
H	-6.8580927878	2.3393656888	-0.0141330098
C	-0.90053697	0.6010130454	0.0196673032
C	1.4789750516	-1.722799406	0.0420788372
H	1.9560304393	-2.6987231451	0.056242859
C	2.2631195027	-0.6108884074	0.0211251908
H	1.7833159252	0.3607665753	0.0154939759
C	0.0543009707	-1.7974686456	0.0491053186
H	-0.3379784499	-2.812413566	0.06448996
C	3.7106300452	-0.6005783107	0.0068383212
C	4.4917078102	-1.7664067353	-0.0312959045

C	4.3862473768	0.6473681887	0.0284989346
C	5.8741683673	-1.7192409659	-0.0446357008
H	3.9998837699	-2.7325940219	-0.0553266213
C	5.7806806152	0.6952347399	0.0153665464
C	6.5152964855	-0.4826772011	-0.0203566735
H	6.4511956605	-2.6365741617	-0.0754829256
H	6.2986244172	1.6455845149	0.0329487924
H	7.5990990217	-0.4299123498	-0.0305997866
O	3.6092759151	1.7463442354	0.0632008477
C	4.2285533244	3.0221084822	0.0844142367
H	3.4153422144	3.7453700804	0.1083699585
H	4.850889188	3.1447353189	0.9761963337
H	4.8328053206	3.1835491306	-0.8135869401
C	-2.969665198	-0.1572714692	0.0277255514
O	-2.2268334391	0.992933034	0.0129360895
O	-0.0166269574	1.4178202798	0.0113860695
N	-2.2855615856	-1.2457001657	0.0433993306

THF

C	-0.9461007835	-0.863577224	0.0394885209
C	-5.0127143168	1.2473370493	0.0060711593
C	-4.4144079119	-0.0166846139	0.0231986265
C	-5.2148080781	-1.165271899	0.033710133
C	-6.5950418502	-1.0464766039	0.0270654639
C	-7.1895719258	0.2143160106	0.0098832743
C	-6.3968320138	1.3574638613	-0.000526569
H	-4.392577919	2.1362235627	-0.0022100451
H	-4.7402605277	-2.1399744556	0.0467391636
H	-7.2126001341	-1.9383311306	0.0350866578
H	-8.2708966562	0.3037983402	0.004571596
H	-6.85768176	2.3393607415	-0.0139577011
C	-0.8993568168	0.6019783164	0.0230153465
C	1.4787117193	-1.7222568023	0.0422219257
H	1.9561507209	-2.6981114663	0.0556826161
C	2.2628714637	-0.6107414735	0.0217267119
H	1.7838502003	0.3613916463	0.0166493806
C	0.0537964408	-1.7973263954	0.0491774952
H	-0.3392840441	-2.8120065961	0.0632830905
C	3.7106994683	-0.6013082218	0.0071451627
C	4.4921087014	-1.7664479321	-0.0306073338
C	4.3860611014	0.6465423612	0.0280722422
C	5.8746622059	-1.7191695879	-0.0442166102

H	4.0002883383	-2.7326854985	-0.0540793156
C	5.7804178046	0.694771625	0.0146438883
C	6.5155552902	-0.4827846174	-0.0206555243
H	6.4518053916	-2.6364614107	-0.0746897696
H	6.2976725067	1.6455455324	0.0316254064
H	7.5993792826	-0.4299103927	-0.0311330243
O	3.6083346538	1.7445904736	0.0622382111
C	4.2249539319	3.0203560496	0.0822618868
H	3.4104173577	3.7421287231	0.1057131029
H	4.8473969879	3.1451962894	0.9739617745
H	4.8289746851	3.1824706794	-0.8160702846
C	-2.9686759297	-0.1561898036	0.0290691501
O	-2.2265209249	0.9940370332	0.016297736
O	-0.0153256882	1.4173724473	0.0163434982
N	-2.2854939717	-1.2452006174	0.0435129568

Water

C	-0.9460463299	-0.8635959101	0.0365705188
C	-5.0138563502	1.246517094	0.0043833389
C	-4.4157840514	-0.0178494359	0.0220386568
C	-5.2168676844	-1.1662439551	0.0350230501
C	-6.597209187	-1.0469595575	0.0303379494
C	-7.1913289625	0.2142076579	0.012746481
C	-6.3980594194	1.3572039601	-0.0001530006
H	-4.3943972283	2.1358377623	-0.0057848112
H	-4.7444218534	-2.1419767173	0.0484018274
H	-7.2149935365	-1.9385653736	0.0402467152
H	-8.2725701316	0.3040646943	0.0090388725
H	-6.8584610393	2.3392502673	-0.0139038562
C	-0.9011883451	0.600644146	0.0162240072
C	1.4790805517	-1.722967675	0.0409624255
H	1.9559454592	-2.6989371551	0.0551547182
C	2.2632252626	-0.6108678706	0.0204193466
H	1.7830344613	0.3605547358	0.0147609217
C	0.0545112125	-1.7974280162	0.0479856534
H	-0.3374108298	-2.8124865039	0.0641825326
C	3.7105944314	-0.600173826	0.0067294218
C	4.491458416	-1.7663824203	-0.0312375179
C	4.3864205084	0.6477661691	0.0289686038
C	5.873877849	-1.7193550327	-0.0440006989
H	3.9995706716	-2.7325155316	-0.055562113
C	5.7808873933	0.6953859755	0.0163828868

C	6.5151923729	-0.4827476007	-0.0192692827
H	6.4508018402	-2.6367485434	-0.07473896
H	6.2992102591	1.6455099161	0.0343838546
H	7.5989897061	-0.4301038622	-0.0290555582
O	3.6098731183	1.7472717372	0.063739163
C	4.2304736007	3.0230039581	0.0855333133
H	3.4179115556	3.7470221036	0.1094725693
H	4.8525753787	3.1446444505	0.9774728311
H	4.8350638246	3.1840017334	-0.8121804659
C	-2.9701809655	-0.1577418786	0.0262149211
O	-2.2270421718	0.992486389	0.0097365773
O	-0.0173066691	1.4180965197	0.0064034945
N	-2.285616118	-1.245858404	0.0426816127

2. Charge-transfer state

Bu_2O

C	-0.887987401	-1.0769641764	-0.3376421319
C	-4.8199035384	1.274161275	-0.3830837265
C	-4.2350878341	0.0400027944	-0.0453812009
C	-5.0374466558	-0.9768540239	0.5067861615
C	-6.3869460547	-0.7580951748	0.7132868849
C	-6.9614984864	0.4683102642	0.3758929077
C	-6.1725339205	1.4793769973	-0.171768081
H	-4.2039724181	2.0580919449	-0.808605353
H	-4.5787487757	-1.9244210805	0.7652204889
H	-7.0002892134	-1.5449698763	1.1397408188
H	-8.0210364995	0.6350560202	0.5397698278
H	-6.6185909164	2.4329449369	-0.4342078708
C	-0.7832546771	0.2582632864	-0.9045247039
C	1.4789726238	-1.954171605	-0.1569248591
H	2.0360428777	-2.8865422243	-0.1451326253
C	2.1909525636	-0.749706991	-0.0296046404
H	1.6320135748	0.1730158597	0.0527799411
C	0.1041933856	-2.0889587729	-0.221259145
H	-0.3022216094	-3.0943578861	-0.1461539897
C	3.5985774812	-0.6377409656	0.0018856992
C	4.4845113695	-1.7299779565	-0.1886303881
C	4.1930530485	0.6526711902	0.2262241184
C	5.851413218	-1.5676867477	-0.1591275457
H	4.0723168621	-2.715263201	-0.3732588298
C	5.5760009126	0.8054457222	0.2500570673
C	6.3994275325	-0.298382162	0.0605439385

H	6.5036324446	-2.4209316934	-0.3101542506
H	6.0175229631	1.7794854245	0.4187568118
H	7.4763665792	-0.1678188303	0.0835649006
O	3.3316639673	1.6584650716	0.4094300897
C	3.8306752932	2.9719635648	0.5981670711
H	2.9538579698	3.6090901807	0.6966329
H	4.435066083	3.0345616658	1.5086182116
H	4.4222853398	3.2931914867	-0.2645255408
C	-2.8380414982	-0.1966796674	-0.252791733
O	-2.0760437836	0.7811695792	-0.7862408123
O	0.0978002216	0.9010638039	-1.4264266357
N	-2.176786029	-1.3028440334	0.0183962244

DMF

C	-0.8936394894	-1.0650743792	-0.3713873279
C	-4.8362254891	1.2688687446	-0.3736486255
C	-4.2442722362	0.0350446846	-0.0432081619
C	-5.0394540065	-0.9839565289	0.5176428651
C	-6.3871234637	-0.7680404396	0.738919636
C	-6.9678956905	0.4578537479	0.4084124697
C	-6.1869442112	1.4712266392	-0.1473446898
H	-4.2287109158	2.05605222	-0.8051245178
H	-4.5797500332	-1.932172795	0.7716572582
H	-6.9941300161	-1.5563172299	1.1714722807
H	-8.0257706071	0.6222708066	0.5840316581
H	-6.6376988364	2.4239794545	-0.4040774613
C	-0.8059100242	0.2698496111	-0.9388004688
C	1.4788956165	-1.939840052	-0.1986445892
H	2.0332655182	-2.8737042559	-0.1893687388
C	2.1971531099	-0.7366333518	-0.0622479883
H	1.6393958746	0.1872190567	0.0158504705
C	0.1066484488	-2.0748250392	-0.2672733497
H	-0.2946485985	-3.0828953155	-0.2019778333
C	3.6020229065	-0.6329666062	-0.0081074738
C	4.4850556236	-1.7355119837	-0.1675526459
C	4.202941265	0.6578553457	0.2151854075
C	5.8509110036	-1.5822356805	-0.1079978488
H	4.070796271	-2.7205444267	-0.3476367861
C	5.5860389814	0.7993234649	0.2719246048
C	6.4037622205	-0.313223756	0.1130515412
H	6.5004118239	-2.4414393652	-0.2340708317
H	6.0322914495	1.7708558	0.4417336339

H	7.4807591855	-0.1909851941	0.1609970859
O	3.3470988886	1.6729045529	0.3626137128
C	3.8557954406	2.9830881742	0.5766894862
H	2.9833107489	3.6282971369	0.656139285
H	4.4339337114	3.0294240756	1.5039339979
H	4.4751414751	3.3028487706	-0.2660289805
C	-2.8506250745	-0.1975575252	-0.2673197083
O	-2.0966747486	0.784017113	-0.8110725333
O	0.0665133121	0.9198938842	-1.4726374664
N	-2.1767124339	-1.2989853584	-0.0044173664

DMSO

C	-0.8936394894	-1.0650743792	-0.3713873279
C	-4.8362254891	1.2688687446	-0.3736486255
C	-4.2442722362	0.0350446846	-0.0432081619
C	-5.0394540065	-0.9839565289	0.5176428651
C	-6.3871234637	-0.7680404396	0.738919636
C	-6.9678956905	0.4578537479	0.4084124697
C	-6.1869442112	1.4712266392	-0.1473446898
H	-4.2287109158	2.05605222	-0.8051245178
H	-4.5797500332	-1.932172795	0.7716572582
H	-6.9941300161	-1.5563172299	1.1714722807
H	-8.0257706071	0.6222708066	0.5840316581
H	-6.6376988364	2.4239794545	-0.4040774613
C	-0.8059100242	0.2698496111	-0.9388004688
C	1.4788956165	-1.939840052	-0.1986445892
H	2.0332655182	-2.8737042559	-0.1893687388
C	2.1971531099	-0.7366333518	-0.0622479883
H	1.6393958746	0.1872190567	0.0158504705
C	0.1066484488	-2.0748250392	-0.2672733497
H	-0.2946485985	-3.0828953155	-0.2019778333
C	3.6020229065	-0.6329666062	-0.0081074738
C	4.4850556236	-1.7355119837	-0.1675526459
C	4.202941265	0.6578553457	0.2151854075
C	5.8509110036	-1.5822356805	-0.1079978488
H	4.070796271	-2.7205444267	-0.3476367861
C	5.5860389814	0.7993234649	0.2719246048
C	6.4037622205	-0.313223756	0.1130515412
H	6.5004118239	-2.4414393652	-0.2340708317
H	6.0322914495	1.7708558	0.4417336339
H	7.4807591855	-0.1909851941	0.1609970859
O	3.3470988886	1.6729045529	0.3626137128

C	3.8557954406	2.9830881742	0.5766894862
H	2.9833107489	3.6282971369	0.656139285
H	4.4339337114	3.0294240756	1.5039339979
H	4.4751414751	3.3028487706	-0.2660289805
C	-2.8506250745	-0.1975575252	-0.2673197083
O	-2.0966747486	0.784017113	-0.8110725333
O	0.0665133121	0.9198938842	-1.4726374664
N	-2.1767124339	-1.2989853584	-0.0044173664

Et_2O

C	-0.8904692684	-1.0717881854	-0.348534604
C	-4.8265909499	1.2725759729	-0.3783514292
C	-4.2391095426	0.0382200309	-0.0448380824
C	-5.0388731804	-0.980468057	0.5083934313
C	-6.3878975741	-0.7634542375	0.7197638392
C	-6.9648492532	0.4631010579	0.3864497485
C	-6.1787110931	1.476048081	-0.162072416
H	-4.2134724467	2.0583701136	-0.8044603572
H	-4.5793198833	-1.9283870644	0.763912175
H	-6.999001243	-1.551692935	1.1468291111
H	-8.023964844	0.6284577598	0.5541941726
H	-6.6265744416	2.4296292773	-0.4212216765
C	-0.7911795471	0.2640018317	-0.9143953084
C	1.4786747961	-1.94756905	-0.1705835072
H	2.0346342561	-2.8805415364	-0.1586116822
C	2.1931712189	-0.7438966812	-0.041717958
H	1.6355813652	0.180072335	0.0361741702
C	0.1045264936	-2.0825298659	-0.2357958496
H	-0.3000926151	-3.0887732429	-0.1626286996
C	3.6001643425	-0.6358119908	-0.00152164
C	4.4845865254	-1.7321114896	-0.1801674799
C	4.1973873864	0.6543784143	0.2219267749
C	5.8513836134	-1.5736676211	-0.1405321625
H	4.0712443321	-2.7172717422	-0.3626008772
C	5.5806016779	0.8026420522	0.2566407118
C	6.4017060881	-0.304677202	0.0783507337
H	6.5022668239	-2.4294732739	-0.2823866278
H	6.0241179618	1.7758098062	0.4249025275
H	7.4788227211	-0.1774754868	0.1095286019
O	3.3382506797	1.6637458523	0.3930823431
C	3.8411686269	2.9757202299	0.5896506291
H	2.9660838616	3.6160646242	0.6814230824

H	4.4369742399	3.0330303496	1.5058147137
H	4.442226521	3.2959381932	-0.266629513
C	-2.8429868324	-0.1963364023	-0.2576361141
O	-2.0839151636	0.7835366982	-0.7930504522
O	0.0869605877	0.9096894967	-1.4390799086
N	-2.1775692404	-1.3011421124	0.0100895794

Gas phase

C	0.95610200	-0.83750000	0.00548900
C	5.04885700	1.23487800	0.17939500
C	4.41482000	-0.00235300	-0.00697800
C	5.19449500	-1.14751400	-0.23780200
C	6.57407700	-1.05078200	-0.28079000
C	7.20034600	0.18144500	-0.09524400
C	6.43162400	1.32003900	0.13430000
H	4.44587900	2.11779700	0.35829100
H	4.69163600	-2.09741600	-0.38079900
H	7.17010100	-1.93989400	-0.46008400
H	8.28258500	0.25304200	-0.12957500
H	6.91539600	2.28064200	0.27943600
C	0.89802800	0.59912200	0.24420400
C	-1.45199300	-1.71838100	-0.04992500
H	-1.94639500	-2.68864100	-0.07845900
C	-2.23946300	-0.59080300	-0.04102100
H	-1.76728000	0.38224600	-0.03102400
C	-0.04815600	-1.81526800	-0.05452200
H	0.35862800	-2.81940000	-0.13317500
C	-3.69105100	-0.61384100	-0.00252600
C	-4.47234100	-1.74039800	0.17578600
C	-4.42217000	0.60394900	-0.12214300
C	-5.90437100	-1.70985800	0.19873700
H	-3.98933700	-2.70082000	0.31678200
C	-5.83126600	0.64703100	-0.09131000
C	-6.58628400	-0.52256200	0.06931800
H	-6.44043800	-2.64389800	0.32731700
H	-6.33673100	1.59910200	-0.20294600
H	-7.66771900	-0.48056600	0.08861200
O	-3.67082200	1.69273500	-0.26528900
C	-4.24577900	2.98641900	-0.30530800
H	-3.40478500	3.67393400	-0.36269700
H	-4.88032000	3.10568900	-1.18941200
H	-4.82306500	3.18453900	0.60280900

C	2.97791500	-0.11684500	0.03603700
O	2.24364100	1.00019200	0.24839900
O	0.00712800	1.42791600	0.42127000
N	2.27598900	-1.20482400	-0.11061300

TMP

C	-0.8801269226	-1.1000265151	-0.3111782464
C	-4.7956662353	1.2760316542	-0.4062994203
C	-4.2200587379	0.0440555755	-0.047864523
C	-5.0305077459	-0.9606213902	0.5133548654
C	-6.3800226898	-0.731796988	0.708888984
C	-6.9459251547	0.4925032542	0.3509660173
C	-6.1484477084	1.4913661672	-0.2061006868
H	-4.1720788242	2.049835643	-0.8392216055
H	-4.5768568517	-1.9063251814	0.7872893888
H	-7.0001039082	-1.5093265348	1.1428018853
H	-8.0055477662	0.6671763916	0.5061586658
H	-6.5879095597	2.4434568524	-0.4847537242
C	-0.7614666953	0.2280343252	-0.8921068833
C	1.4809282827	-1.9780244471	-0.1217627565
H	2.0434376727	-2.9073113125	-0.1121617475
C	2.1835665257	-0.7690119076	0.0034402027
H	1.6182756528	0.1475938064	0.1060654081
C	0.1054963975	-2.1153374352	-0.1836391236
H	-0.3034811175	-3.119373684	-0.1039336494
C	3.5914760836	-0.6423542711	0.0087426592
C	4.4833681498	-1.7211329844	-0.2183798141
C	4.1762418648	0.6497918566	0.2386632016
C	5.8494321338	-1.5451492405	-0.2162372754
H	4.0761740488	-2.7069337781	-0.4115680038
C	5.557544061	0.8176353878	0.233705819
C	6.3886139953	-0.274230719	0.0100397961
H	6.5067122515	-2.3891227376	-0.3950387919
H	5.9920874856	1.7941670221	0.4064973671
H	7.4643694201	-0.132184254	0.0113869857
O	3.3076373035	1.643526191	0.4564548698
C	3.7942497796	2.9610254553	0.6356602597
H	2.9123587503	3.5871630722	0.7583970899
H	4.4215409105	3.030578425	1.5304613324
H	4.3592259638	3.2928471924	-0.2411828377
C	-2.8221900031	-0.2034856108	-0.2433125273
O	-2.0516836516	0.7630263974	-0.782890383

O	0.1275038727	0.8575005231	-1.4151731608
N	-2.1722100339	-1.3116022012	0.0441403624

MCH

C	-0.8813665306	-1.0959325869	-0.3141595101
C	-4.7998341759	1.2756799753	-0.4028879068
C	-4.2226811953	0.0434768113	-0.04745262
C	-5.0318628048	-0.9633078421	0.5119798522
C	-6.3815188907	-0.7363543955	0.7086528898
C	-6.9488986338	0.4881631031	0.3536986962
C	-6.1527371854	1.489127489	-0.2015472313
H	-4.1773798792	2.0511841445	-0.8343856363
H	-4.5772446476	-1.9091913275	0.7836516316
H	-7.0005643386	-1.5154986721	1.1411327287
H	-8.0086263039	0.6613629887	0.5097853314
H	-6.5933385742	2.4413659182	-0.4778631213
C	-0.7647242614	0.2334637855	-0.892550835
C	1.4805592492	-1.9740184459	-0.1260601487
H	2.0420933864	-2.903869612	-0.1158955049
C	2.1847767968	-0.7658307903	-0.0009562991
H	1.6206109696	0.1518578512	0.0984379121
C	0.1051332143	-2.1107424634	-0.1881257613
H	-0.3036359159	-3.1148876494	-0.1087801317
C	3.5927675974	-0.6416231394	0.0078201277
C	4.4835633409	-1.7225115527	-0.2143504859
C	4.1792321598	0.6501098601	0.2368616399
C	5.8498506134	-1.5488210816	-0.2085948871
H	4.0753947521	-2.7081220785	-0.4064048556
C	5.5608542633	0.8154918187	0.2356962613
C	6.3906177889	-0.2782815067	0.0166807427
H	6.5062171588	-2.3942864637	-0.383655016
H	5.9966181678	1.791581221	0.4078580775
H	7.4666119849	-0.1381294327	0.0208873679
O	3.311842492	1.6457676074	0.4500122579
C	3.8005060122	2.9626075061	0.6300539993
H	2.9194359865	3.5905294168	0.7493258268
H	4.4247860715	3.0313363599	1.5269695677
H	4.3691869429	3.292603766	-0.2450369035
C	-2.8247635149	-0.2020848118	-0.2441749184
O	-2.0555409497	0.7664211067	-0.7822247441
O	0.1231051048	0.8652496415	-1.4148695293
N	-2.1730892513	-1.3099225187	0.0407811357

MeAc

C	-0.8933784927	-1.0654629766	-0.3708665768
C	-4.8354870159	1.2691943015	-0.3719210784
C	-4.2438372012	0.0348802733	-0.0431147504
C	-5.0392921952	-0.9849619927	0.5156382778
C	-6.3870661138	-0.7693582124	0.7365220367
C	-6.9676113746	0.4570479995	0.4076629669
C	-6.1863147018	1.4712409762	-0.1460316742
H	-4.227540189	2.0569034727	-0.8018274352
H	-4.579436151	-1.9334673341	0.7682965762
H	-6.9943369646	-1.5583180172	1.1674824756
H	-8.0255877287	0.621237057	0.5829548788
H	-6.6368862046	2.4244319809	-0.4015036675
C	-0.804675937	0.2702832911	-0.9364388
C	1.4790246034	-1.9398078488	-0.1988733344
H	2.033829032	-2.8734220653	-0.1903508807
C	2.196714501	-0.7366119442	-0.0619723549
H	1.6390795521	0.1872610679	0.016782725
C	0.1066020343	-2.0751779984	-0.2671660963
H	-0.2946797355	-3.0832672705	-0.2021272127
C	3.601841419	-0.6326292986	-0.0087890368
C	4.4849986276	-1.7343420166	-0.171496225
C	4.2023667658	0.6577944019	0.2164231189
C	5.8509761461	-1.5806827527	-0.1136228204
H	4.0708102146	-2.719125769	-0.3532384288
C	5.5855081103	0.7998104317	0.2712515818
C	6.4035767196	-0.3120042222	0.1089524657
H	6.500620212	-2.439388365	-0.2424042927
H	6.0314889368	1.7712726702	0.442255905
H	7.480602746	-0.1893647342	0.1554345564
O	3.3462172763	1.6720450564	0.3678076117
C	3.854364522	2.9820291063	0.582249698
H	2.981629049	3.6266508807	0.663859134
H	4.4342187387	3.0279587033	1.50853181
H	4.4719544934	3.3031665362	-0.2612928317
C	-2.8499599604	-0.1974997408	-0.2666936118
O	-2.0957487286	0.784681205	-0.8085269475
O	0.0681696719	0.92054514	-1.468632254
N	-2.1767976773	-1.2995779924	-0.0049055084

THF

C	-0.8919573398	-1.0689588457	-0.3609926625

C	-4.8313310449	1.2708199132	-0.3759921914
C	-4.2415538763	0.0367893028	-0.0439047192
C	-5.0388154781	-0.9818732149	0.5138333382
C	-6.387055824	-0.765292682	0.7306662963
C	-6.9659859348	0.4608659666	0.3986373204
C	-6.1826374815	1.4738360406	-0.1541597545
H	-4.221049465	2.0570450351	-0.8053392198
H	-4.5788331046	-1.9297677491	0.7686012432
H	-6.9959589771	-1.5534210827	1.1609736622
H	-8.0244056412	0.6258588116	0.5707207315
H	-6.6320008562	2.4269655176	-0.4122015681
C	-0.7984921186	0.2665525601	-0.9277168957
C	1.4789435461	-1.9438668697	-0.1867612674
H	2.0343885949	-2.8770968216	-0.1758985207
C	2.1948602322	-0.7403318635	-0.0546275629
H	1.6375073936	0.1839139558	0.0214572275
C	0.1056052349	-2.0792035685	-0.2531744942
H	-0.2972548348	-3.0864026032	-0.1836259409
C	3.6009953275	-0.6344217295	-0.0054678413
C	4.4848145931	-1.7338915749	-0.1726217954
C	4.1996687398	0.6561647254	0.2180660029
C	5.8512144405	-1.5779767757	-0.1220954199
H	4.0712185847	-2.7192026267	-0.3533397163
C	5.5828568734	0.8012060189	0.2643346614
C	6.4025355049	-0.3087915613	0.0970557786
H	6.5014818596	-2.4356624404	-0.2548154551
H	6.0274336828	1.773843815	0.4326461334
H	7.4795924603	-0.1838618277	0.136981109
O	3.3422145244	1.6685627503	0.3767089912
C	3.8479247843	2.9791956414	0.5836543471
H	2.9743047736	3.6220926211	0.6704679846
H	4.4354174824	3.0298970326	1.5052038214
H	4.4578066391	3.3006954379	-0.2656388549
C	-2.8466796926	-0.1970559291	-0.2628438584
O	-2.0904124313	0.783341995	-0.8033124234
O	0.0766163749	0.9134926306	-1.4578209664
N	-2.1770205462	-1.3000960052	0.0026524795

Water

C	-0.8939689766	-1.065076439	-0.372737202
C	-4.8367023595	1.268630636	-0.368972788
C	-4.2442453217	0.0348303115	-0.0392082137

C	-5.0386228803	-0.9840073625	0.5231958763
C	-6.385913867	-0.7679430429	0.7466600637
C	-6.9671464586	0.4579452558	0.4168190489
C	-6.1870480422	1.4711435104	-0.1404862296
H	-4.2299961847	2.0557819055	-0.8016339753
H	-4.5788164849	-1.9322939661	0.7767488663
H	-6.9922550972	-1.5560677958	1.180391755
H	-8.0247012408	0.6224809041	0.5941649271
H	-6.6381544683	2.423853853	-0.3966998297
C	-0.8075115224	0.2697327538	-0.9403202025
C	1.4787854159	-1.9398766286	-0.2023372189
H	2.0332847283	-2.8736662287	-0.194298812
C	2.1971602478	-0.7365668153	-0.0652220853
H	1.6391635609	0.1869954193	0.0143430629
C	0.1067071402	-2.0750078292	-0.270481775
H	-0.2941998703	-3.0832866478	-0.2063102903
C	3.6018750358	-0.6329008768	-0.0112705238
C	4.4848956829	-1.7357876146	-0.169593239
C	4.2029662345	0.6582150977	0.2110890187
C	5.8506620715	-1.5826049737	-0.1093566738
H	4.0706076672	-2.7209420644	-0.3488516874
C	5.5860333445	0.7994413776	0.2687204385
C	6.4035719424	-0.3134124248	0.1112286801
H	6.5001714036	-2.4419481741	-0.2343041657
H	6.0324330076	1.7709907669	0.4379960745
H	7.4805497005	-0.1913609056	0.1598511876
O	3.3473049671	1.6736313265	0.3565247796
C	3.8563183348	2.9838506923	0.5715085334
H	2.9839490503	3.6292316028	0.6506091155
H	4.4337542279	3.0295290951	1.4991427045
H	4.4763600081	3.3036189933	-0.2706452847
C	-2.8510434421	-0.1978580026	-0.2655986616
O	-2.0979030665	0.7836800272	-0.8108695389
O	0.0640198763	0.9201037325	-1.475700047
N	-2.1763873649	-1.2991154686	-0.0037856882

Table S13. The number of imaginary frequencies for structures in the ground state.

<table>
<tr><th></th><th>Z (cis)</th><th>E (trans)</th></tr>
<tr><td>Gas phase</td><td rowspan="11" colspan="2">no imaginary frequency, structure at an energy minimum on the surface of potential energy</td></tr>
<tr><td>Izooctane</td></tr>
<tr><td>MethylCycloHexane</td></tr>
<tr><td>Bu2O</td></tr>
<tr><td>Et2O</td></tr>
<tr><td>THF</td></tr>
<tr><td>MeAc</td></tr>
<tr><td>MeCN</td></tr>
<tr><td>DMF</td></tr>
<tr><td>DMSO</td></tr>
<tr><td>Water</td></tr>
</table>

Table S14. Computed total energies of optimized structures in the ground state. Values are given in a.u.

	Z (*cis)*	*E* (*trans)*
Gas phase	-1012.5316858	-1012.5320619
Izooctane	-1012.5355999	-1012.5368112
MethylCycloHexane	-1012.5358364	-1012.5370935
Bu2O	-1012.5377965	-1012.5394440
Et2O	-1012.5390936	-1012.5409916
THF	-1012.5407368	-1012.5429524
MeAc	-1012.5423692	-1012.5449129
MeCN	-1012.5428079	-1012.5454048
DMF	-1012.5428329	-1012.5453023
DMSO	-1012.5428328	-1012.5455748
Water	-1012.5431411	-1012.5457970

Table S15. The number of imaginary frequencies for the transition state structure.

Gas phase	one imaginary frequency: -8.41 cm^{-1}
Water	one imaginary frequency: -9.73 cm^{-1}

Table S16. Computed total energies of the transition state. Values are given in a.u.

Gas phase	-1012.5316858
Water	-1012.5431411

List of references:

1. Bilot, L.; Kawski, A., Zur Theorie Des Einflusses Von Lösungsmitteln Auf Die Elektronenspektren Der Moleküle. *Z. Naturforsch.* **1962**, *17A*, 621-627.
2. Bilot, L.; Kawski, A., Der Einfluß Des Lösungsmittels Auf Die Elektronenspektre Lumineszierender Moleküle. *Z. Naturforsch.* **1963**, *18A* 10-15.
3. Bilot, L.; Kawski, A., Dipolmomente Einiger Phthalimid-Derivate Im Ersten Angeregten Singulettzustand. *Z. Naturforsch.* **1963**, *18A*, 256.
4. Kawski, A., Solvent-Shift Effect on Electronic Spectra and Excited State Dipole Moments. In *Progress in Photochemistry and Photophysics*, Rabek, J. F., Ed. CRC Press Boca Raton, Ann Arbor Boston, 1992; Vol. V, pp 1–47.

5. Nadaf, Y. F.; Mulimani, B. G.; Gopal, M.; Inamdar, S. R., Ground and Excited State Dipole Moments of Some Exalite Uv Laser Dyes from Solvatochromic Method Using Solvent Polarity Parameters. *Journal of Molecular Structure: THEOCHEM* **2004**, *678*, 177-181.

6. Krawczyk, P.; Czeleń, P.; Cysewski, P., Reactive Group Effects on the Photophysical and Biological Properties of 2-Phenyl-1h-Phenanthro[9,10-D]Imidazole Derivatives as Fluorescent Markers. *Org. Bio. Chem.* **2018**, *16*, 3788-3800.

7. Krawczyk, P.; Czeleń, P.; Jeliński, T.; Cysewski, P., The Influence of Donor Substituents on Spectral Properties and Biological Activities of Fluorescent Markers Conjugated with Protein. *J. Photochem. Photobiol. A* **2018**, *365*, 157-168.
